# Oxygen-dependent regulation of E3(SCF)ubiquitin ligases and a Skp1-associated JmjD6 homolog in development of the social amoeba *Dictyostelium*

**DOI:** 10.1016/j.jbc.2022.102305

**Published:** 2022-08-04

**Authors:** Andrew W. Boland, Elisabet Gas-Pascual, Braxton L. Nottingham, Hanke van der Wel, Nitin G. Daniel, M. Osman Sheikh, Christopher M. Schafer, Christopher M. West

**Affiliations:** 1 Department of Biochemistry & Molecular Biology, University of Georgia, Athens, Georgia, USA; 2 Complex Carbohydrate Research Center, University of Georgia, Athens, Georgia, USA; 3 Center for Tropical and Emerging Global Diseases, University of Georgia, Athens, Georgia, USA; 4 Department of Biochemistry & Molecular Biology, University of Oklahoma Health Sciences Center, Oklahoma City, Oklahoma, USA

**Keywords:** cellular slime mold, E3(SCF)ubiquitin-ligase, F-box protein, glycosylation, Jumonji C, prolyl hydroxylase, Skp1, oxygen, αKG, α-ketoglutarate, Ab, antibody, ACN, acetonitrile, cDNA, complementary DNA, co-IP, co-immunoprecipitation, DMSO, dimethyl sulfoxide, FDR, false discovery rate, gDNA, genomic DNA, LRR, leucine rich repeat, MS, mass spectrometry, Ub, ubiquitin

## Abstract

E3-SCF (Skp1/cullin-1/F-box protein) polyubiquitin ligases activate the proteasomal degradation of over a thousand proteins, but the evolutionary diversification of the F-box protein (FBP) family of substrate receptor subunits has challenged their elucidation in protists. Here, we expand the FBP candidate list in the social amoeba *Dictyostelium* and show that the Skp1 interactome is highly remodeled as cells transition from growth to multicellular development. Importantly, a subset of candidate FBPs was less represented when the posttranslational hydroxylation and glycosylation of Skp1 was abrogated by deletion of the O_2_-sensing Skp1 prolyl hydroxylase PhyA. A role for this Skp1 modification for SCF activity was indicated by partial rescue of development, which normally depends on high O_2_ and PhyA, of *phyA-*KO cells by proteasomal inhibitors. Further examination of two FBPs, FbxwD and the Jumonji C protein JcdI, suggested that Skp1 was substituted by other factors in *phyA-*KO cells. Although a double-KO of *jcdI* and its paralog *jcdH* did not affect development, overexpression of JcdI increased its sensitivity to O_2_. JcdI, a nonheme dioxygenase shown to have physiological O_2_ dependence, is conserved across protists with its F-box and other domains, and is related to the human oncogene JmjD6. Sensitization of JcdI-overexpression cells to O_2_ depended on its dioxygenase activity and other domains, but not its F-box, which may however be the mediator of its reduced levels in WT relative to Skp1 modification mutant cells. The findings suggest that activation of JcdI by O_2_ is tempered by homeostatic downregulation *via* PhyA and association with Skp1.

For cells to adapt to their surroundings and proliferate or differentiate, their proteomes must be selectively modified. Protein degradation is largely mediated by autophagy and the ubiquitin (Ub) proteasome system. For the latter, the SCF (Skp1/cullin-1/F-box protein/Rbx1) family of E3 Ub ligases, conserved throughout eukaryotes, tags specific proteins with lysine 48 (K48)-linked polyubiquitin chains for degradation by the 26S proteasome (1, 2) (Fig. 1*A*). The specificity of these complexes is mediated by the family of F-box proteins (FBPs) that serve as substrate receptors (3). They are distinguished by the presence of a conserved 40 aa F-box domain that binds to the C-terminal region of Skp1 (4). Protein–protein interaction domains that are commonly found in substrate receptor FBPs include WD40 repeats and leucine rich repeats (LRRs), are referred to as Fbxw# and Fbxl#, respectively, and other FBPs contain ankyrin, kelch, and armadillo repeats as putative substrate receptor domains. Some Fbxo#-type FBPs lack archetypical protein–protein interaction domains and may be substrates themselves (5). In humans, the 69 identified FBPs likely regulate the half-lives of over a thousand proteins. Contrary to the highly conserved Cul1, Skp1, Rbx1, and E2 proteins, FBPs can be challenging to predict owing to extensive diversification of their sequences across eukaryotic phylogeny (6), suggesting a tendency to assume organism specific roles.

**Figure 1:**
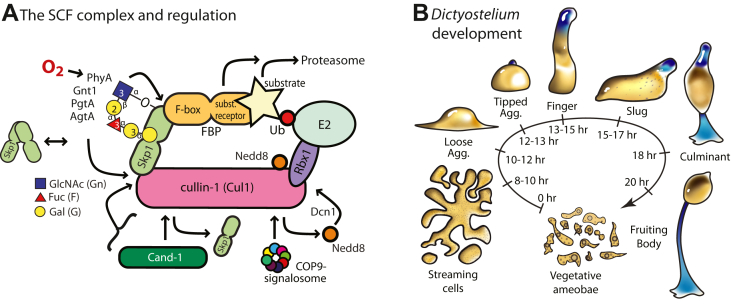
**The SCF complex and *Dictyostelium* development.***A,* schematic illustration of a generic SCF complex including modes of regulation based on studies in yeast, mammalian cells, and plant cells (1, 2, 50, 81, 82). The novel regulation explored here involves the posttranslational prolyl hydroxylation and glycosylation of Skp1 in protists, including *Dictyostelium*. *B*, schematic illustration of the starvation-induced developmental cycle of *Dictyostelium discoideum*. Prestalk cells and anterior-like cells are labeled *blue* (83). The life cycle is renewed when spores from a fruiting body germinate to become feeding amoebae.

The Skp1 adapter of SCF complexes of multiple unicellular eukaryotes is uniquely modified by a linear pentasaccharide assembled on a critical prolyl residue following its hydroxylation by the O_2_-sensing prolyl 4-hydroxylase PhyA (7, 8), a likely ortholog of the human O_2_-sensor PHD2 (9, 10). First described in the social amoeba *Dictyostelium discoideum* (7), a related modification is found in the apicomplexan parasite *Toxoplasma gondii* (11, 12), the crop plant parasite, *Pythium ultimum* (13), and likely many other aerobic protists (8).

The O_2_-dependent development of *Dictyostelium* has made it a favorable model for expanding our understanding of O_2_-sensing of protist pathogens and other eukaryotes (8). We previously showed that Skp1 hydroxylation and glycosylation increased the representation of several FBPs in the interactome of Skp1 as assessed by coimmunoprecipitation (co-IP) coupled with proteomics analysis (14). However, it was unclear whether this was best explained as a binding equilibrium effect or abundance of the interactors and, furthermore, what was the interaction dynamic? Here, we expand our approach using orthogonal Skp1 capture methods applied to both growing and developing cells, and follow by examining characteristics of two predicted FBPs from the interactome. We find that the Skp1 modification appears to influence binding equilibrium in cells but with varied effects on the abundance of the two interactors.

One candidate is JcdI, a Jumonji C (JmjC) domain containing nonheme dioxygenase most related to human JmjD6. JmjD6 is also an O_2_-dependent enzyme and has been implicated in epigenetic regulation, RNA splicing, and oncogenesis (15, 16, 17). JcdI was an interesting candidate because other O_2_-sensing mechanisms are indicated by the observation that the absence of PhyA can be overridden by hyperoxia (18). We show that unlike JmjD6, JcdI is an FBP and, like PhyA, is an O_2_-dependent dioxygenase whose enzyme activity is likely controlled by physiological variations in O_2_. JcdI is to our knowledge the first FBP found to be conserved across the protist kingdom and higher plants. The previously reported decreased representation of JcdI in the interactome of Skp1 from WT (*phyA*^+^) *versus phyA*^–^ cells (14) is attributable to a decreased level of JcdI that obscured increased interaction with Skp1. Significantly, overexpression of JcdI decreased the O_2_ threshold for fruiting body formation. This effect depended on its catalytic activity but not its F-box, suggesting that O_2_ regulates endogenous JcdI both directly as a substrate and indirectly by controlling its level *via* PhyA and Skp1.

An additional question posed by the differential representation of FBPs in the Skp1 interactome of glycosylation competent *Dictyostelium* is whether the effect on organismic O_2_ sensing was actually mediated *via* the Ub-proteasome system. Here, we show that the altered O_2_ threshold for the development of *phyA*^–^ cells can be partially restored by chemical inhibition of the proteasome. This compensatory effect implicates the activity of the SCF complex, which generates the K48-linked Ub chains that direct proteins to the proteasome, as an intermediary in this cellular response mechanism.

## Results

### Proteasome inhibitors bypass the dependence of culmination on PhyA

*Dictyostelium* is a social amoeba that normally proliferates in the presence of a bacterial food source or, for axenic mutants, in a rich liquid broth. In response to starvation, the amoebae, when deposited on a filter surface to ensure a high level of O_2_, chemotactically aggregate *via* streams to form a multicellular slug that eventually, over the course of a few more hours, culminates into a fruiting body consisting of a ball of spores perched on top of a 1 to 2 mm long cellular stalk (Fig. 1*B*). In the absence of sufficient O_2_ or in the absence of PhyA, slugs fail to culminate into fruiting bodies, which reflects the existence of an O_2_ checkpoint that the slug uses to monitor its location in the vertical column of the soil that it normally inhabits (8).

**Figure 2:**
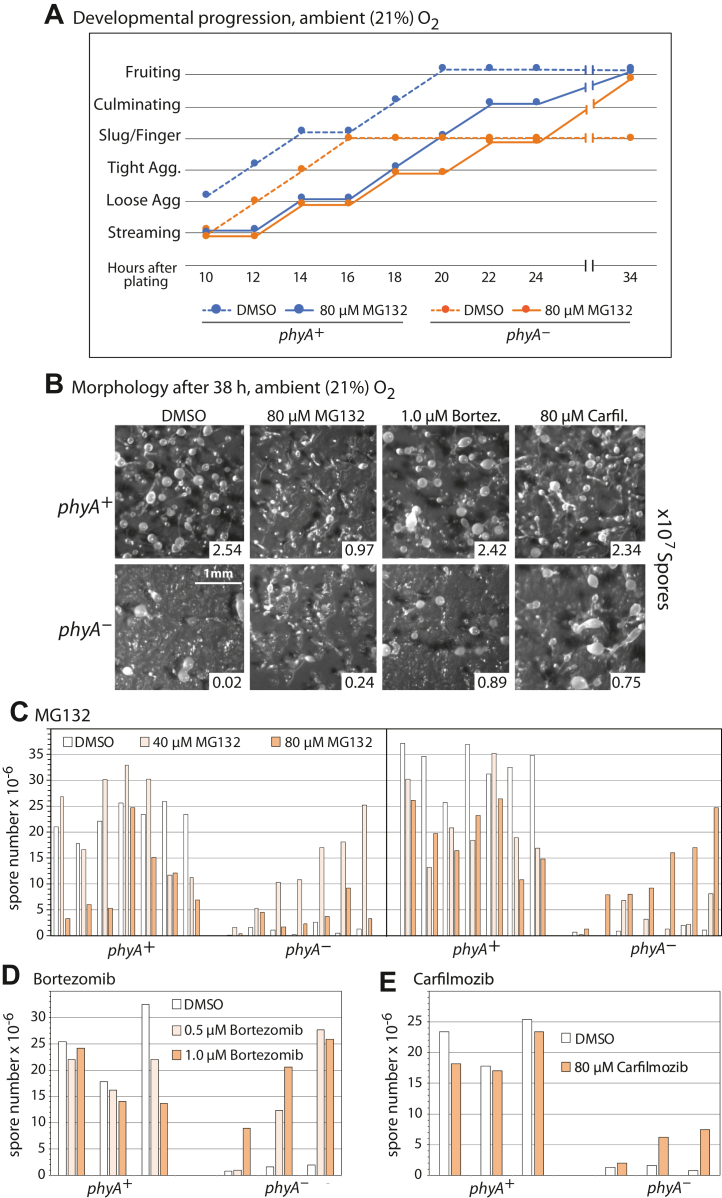
**Effects of proteasome inhibitors on developmental progression.** Amoebae (*phyA*^+^ or *phyA*^–^) were spread on filters in the presence or absence of the indicated concentrations of inhibitors or an equivalent volume of the carrier solvent (DMSO) and allowed to develop. *A,* morphology, observed at 2 h intervals starting at 10 h, was graphed as a function of time. *B,* representative images of morphologies after 38 h in the presence of the indicated concentrations of inhibitors. The spherical structures represent the spore-containing sori of fruiting bodies. Spore counts from the represented trials are shown in the inset. *C–E,* quantitation of spore numbers from all trials. Results from all independent trials are shown and graphed in increasing order of spore numbers produced by inhibitor-treated *phyA*^–^ cells. *phyA*^+^ cells from same trials are at the left in matching order. *C,* trials using 80 μM carfilzomib. *D,* trials using 40 μM and 80 μM MG132. *E,* trials using 0.5 μM and 1.0 μM bortezomib. Trials where maximal spore formation occurred at 40 μM or 80 μM MG132 are in separate panels. DMSO, dimethyl sulfoxide.

In order to examine whether the failure of *phyA*^*–*^ slugs to culminate into fruiting bodies is associated with dysregulation of protein degradation, as might be expected based on evidence that Skp1 is the only PhyA substrate, developing *Dictyostelium* were treated with inhibitors of the proteasome, which mediates the degradation of proteins that are polyubiquitinated by SCF complexes. As shown in Figure 2*A*, under ambient O_2_, *phyA*^–^ cells exhibit a ∼2 h delay through the early phases of multicellular development, as represented on the vertical axis, and fail to progress past the finger/slug stage or produce the normal number of spores, as shown by the examples in Figure 2*B*. However, in the presence of MG132 at a concentration (80 μM) above that which inhibits the proteasome in single *Dictyostelium* cells (19), a substantial fraction of slugs were found to progress to the fruiting body stage and produce spores (Fig. 2, *A* and *B*), despite a 4 to 6 h delay in moving through the earlier stages of development. In contrast, parental *phyA*^+^ cells were simply delayed by 4 to 6 h and were modestly compromised in spore production. In some trials, a greater induction of *phyA*^–^ spores was observed at the lower concentration of 40 μM, whereas 160 μM MG132 resulted in a developmental blockade at the early streaming stage in both strains (data not shown). Though considerable variation was observed over the 16 independent trials where matching pairs of *phyA*^–^ and *phyA*^+^ were analyzed (Fig. 2*C*), increased and usually substantial spore formation by *phyA*^–^ cells was found at either 40 μM (left panel) or 80 μM (right panel) MG132, suggesting that 40 μM MG132 was the minimal concentration for maximal rescue of *phyA*^–^ sporulation. To investigate the specificity of the effect, two proteasome inhibitors that have distinct mechanisms (20, 21, 22, 23) were tested. Similar findings regarding selective induction of sporulation of *phyA*^–^ cells were observed over the range of 0.5 to 1.0 μM bortezomib (Fig. 2, *B* and *D*) and for 80 μM carfilzomib (Fig. 2, *B* and *E*).

**Figure 3:**
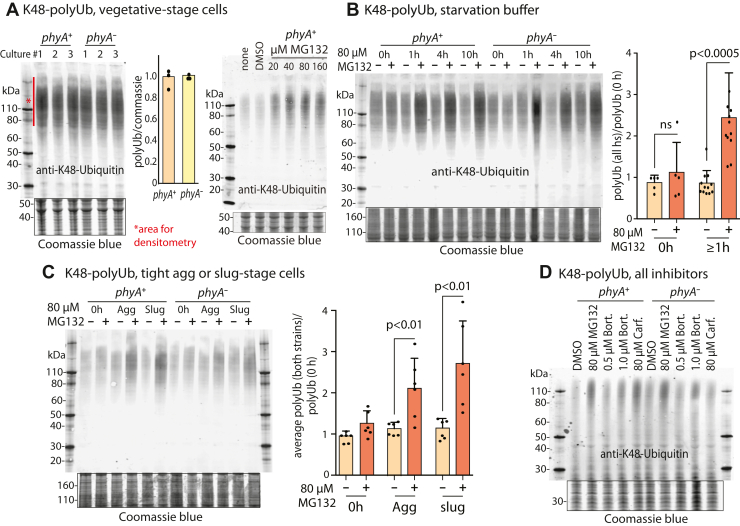
**Effects of proteasome inhibitors on protein polyubiquitination**. Cells were treated with the indicated concentrations of proteasome inhibitors and analyzed by whole cell Western blotting using anti-K48-polyubiquitin (polyUb). Densitometry was performed on the indicated regions and normalized to the Coomassie blue stain intensity of the gel after electrotransfer. *A,* comparison of *phyA*^*+*^ and *phyA*^*–*^ vegetative cells (left). All data points are shown, and the quantitation shows the average ±SD of three independent trials. Response of *phyA*^*+*^ vegetative cells to treatment with the indicated concentrations of MG132 after 2 h (right). *B,* comparison of effects of 80 μM MG132 on cells shaken in starvation buffer over 10 h. For statistical analysis, time points ≥1 h were averaged. *C,* similar analysis of cells starved at an air–water interface, which allows development to the tight aggregate stage (10 h) or slugs (14–16 h, based on morphology). Paired *t* tests were used to determine the significance of differences between pooled data from treated and untreated samples. *D,* comparison of the effects of three proteasome inhibitors, after incubation up to the slug stage of development.

The similar findings from all three compounds suggest that the rescue is the result of proteasome inhibition rather than off-target effects. To test this interpretation, extracts were evaluated for effects on protein polyubiquitination by Western blotting with antibodies (Abs) against Ub or specific for chains of Ub linked *via* K48 of the underlying Ub. These Abs revealed a relatively high level of binding to a higher apparent *M*_*r*_ region of the gel, which is elevated in cells treated with the inhibitors. The level of this labeling was similar between *phyA*^+^ and *phyA*^–^ cells grown vegetatively (Fig. 3*A*) or as slugs (data not shown). Treatment with increasing concentrations of MG132 indicated that accumulation of poly-K48-Ub conjugates plateaued at 40 μM, suggesting that maximal accumulation was required for developmental rescue of *phyA*^–^ cells (Fig. 2*C*). When cells were starved in non-nutrient buffer, 80 μM MG132 rapidly increased polyubiquitin levels in a fashion that plateaued by 1 h and was sustained for at least 10 h (Fig. 3*B*). A similar finding was observed for MG132 in *phyA*^+^ slug cells (Fig. 3*C*). Elevated poly-K48-Ub levels were also detected using 1.0 μM bortezomib and 80 μM carfilzomib (Fig. 3*D*), and lower levels of poly-K48-Ub in slug cells treated with 0.5 μM bortezomib correlated with variable rescue of *phyA*^–^ cells at this lower concentration (Fig. 2*D*). Although *Dictyostelium* has been reported to accumulate poly-K63-Ub during an autophagic response to infection with *Mycobacterium marinum* (24), Western blot labeling of vegetative, aggregation, or slug stage cell extracts (as in Fig. 3*C*) with an Ab specific for poly-K63-Ub chains was minimal and not stimulated by treatment with 80 μM MG132 (data not shown). Thus, although exact thresholds varied between trials, the concentrations of inhibitors that caused maximal steady state levels of poly-K48-Ub conjugated proteins correlated approximately with concentrations required to partially rescue development of *phyA*^–^ cells. However, no reproducible differences in polyubiquitin levels were detected for untreated or treated *phyA*^–^ and *phyA*^+^ cells, suggesting that rescue might have resulted from transient effects on specific targets that were obscured at the level of the entire population.

### The Skp1 interactome of vegetative (proliferating) amoebae

A previous mass spectrometry (MS)–based proteomics study of the Skp1 interactome in slugs (14) identified 18 candidate proteins and showed that the representation of several was influenced by PhyA (Table S2). These proteins are therefore candidates for mediating the role of PhyA and for the rescue of *phyA*^–^ cells by the proteasome inhibitors. However, that study did not address the potentially dynamic nature of the Skp1 interactome, was limited to the use of a single method to immunoprecipitate Skp1, and did not address the basis for differential representation of proteins in *phyA*^+^ *versus phyA*^–^ cells.

As a first step in expanding our understanding of the Skp1 interactome, we applied our previous co-IP method to vegetative cells. This method used an affinity-purified anti-Skp1 antiserum (pAb UOK77) and proteomic analysis of the captured material by nLC/MS-MS and quantitation by spectral counting (14). We analyzed three strains, the normal parental strain (Ax3) and two derivatives that overexpress either His_6_AgtA or FLAG_3_AgtA, which is the terminal Skp1 glycosyltransferase (23). Since the data profiles were indistinguishable, indicating negligible influence of AgtA-overexpression, the samples were analyzed together as biological replicates.

**Figure 4:**
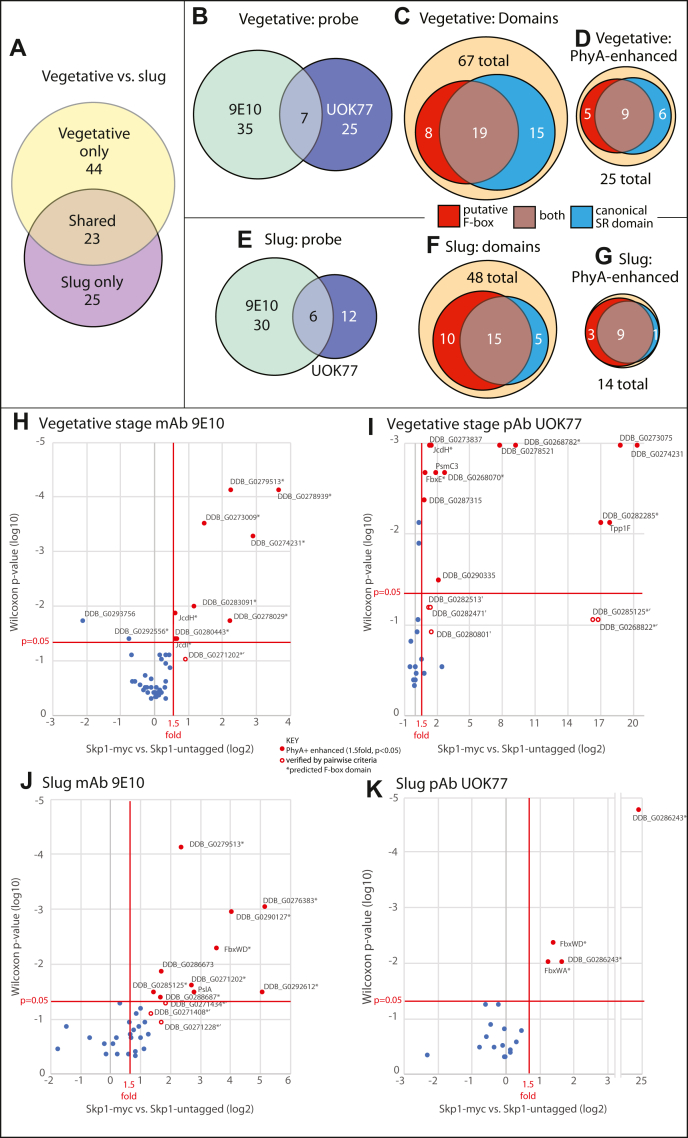
**Dependence of the Skp1 interactome on cell differentiation and Skp1 modification.** The Skp1 interactome was assessed by co-IP with anti-myc (mAb 9E10) from cells expressing Skp1-myc or with anti-Skp1 (affinity-purified pAb UOK77) from strains lacking a tagged Skp1, and captured proteins were detected and quantified by label-free proteomic analysis. Proteins that were enriched relative to a statistically significant extent over control co-IPs (untagged Skp1 for mAb 9E10 and nonspecific IgG for pAb UOK77), as summarized in Table S2, Figs. S1, and S2, are classified according to life cycle stage, the detection method, properties, and dependence on PhyA. *A–G*, Venn diagrams illustrate the overlap of results between the two life cycle stages (panel *A*), overlaps between the two antibodies (panel *B* for vegetative stage cells; *E* for slugs), fraction of interactors possessing predicted F-box and substrate receptor domains (panel *C* for vegetative cells; *F* for slugs), and fraction of the interactors shown in panels *C* and *F* that are enriched in *phyA*^+^*versus phyA*^–^ cells (panel *D* for vegetative stage; G for slug). *H*, Skp1-myc interaction candidates in vegetative cells were plotted according to fold-enrichment in *phyA*^+^*versus phyA*^–^ cells and statistical significance. *Red* data points represent interactors that were found at 1.5-fold higher levels at *t* test and Wilcoxon test *p*-values ≤ 0.05. Open *red* circles represent candidates determined to be *phyA*^*+*^ enhanced by pairwise comparison criteria (Table S2). *I*, same for results from anti-Skp1 and vegetative cells. *J*, same as panel (*H*), using slug cells. *K*, same as panel (*I*), using slug cells. co-IP, coimmunoprecipitation.

Thirty-two proteins were confidently assigned to the Skp1 interactome of vegetative cells (summarized in Figure 4*B*; see Figs. S1, S2, and Table S2 for details), based on the following criteria: (i) ≥2 peptides from the protein were confidently assigned; (ii) peptides and proteins were each detected at a false discovery rate (FDR) upper limit set at 1%; (iii) peptides were detected in the Skp1 co-IPs at a level that was ≥4-fold higher than in co-IPs using a nonimmune rabbit IgG, based on analysis of three technical replicates from each of three independent biological replicates with a Wilcoxon *p*-value of <0.01. Candidates were further filtered to remove mitochondrial, ribosomal and secretory proteins, and other common contaminants (see Experimental procedures).

To complement these findings, we developed an orthogonal capture method by expressing a C-terminally myc-tagged version of Skp1 under a semiconstitutive promoter (discoidin 1γ) and recovering complexes with an anti-myc murine mAb covalently coupled to beads that were collected magnetically rather than centrifugally to reduce nonspecific binding. Western blotting showed that Skp1-myc was expressed at a level comparable to that of native Skp1 (25), thus doubling the total level of Skp1 in the cell. The anti-myc approach identified 42 high confidence candidates in vegetative cells, seven of which overlapped with those from the UOK77 co-IP (Fig. 4*B* and Table S2). The expected SCF subunits, CulE, CulA (Cul1 homologs), and Rbx1, were identified in the anti-myc but absent in the UOK77 co-IPs, suggesting that the myc-tag method was capturing a distinct though partially overlapping set of Skp1 subcomplexes. Combining the results, 67 interactors were identified in vegetative cells (Fig. 4*B*). Their mean relative abundances, summed from peptide counting (26), ranged over 500-fold (Fig. S2*A*). This range might reflect their relative cellular levels, the fraction that interacts with Skp1, the number of tryptic peptides in the protein, and/or the stability of the interaction with Skp1, which may be direct or indirect. However, there was little overall correlation between transcript levels and representation in the Skp1 interactome (Fig. S2 and Table S2).

### The Skp1 interactome differs between vegetative and slug cells

Only 6 of the 32 vegetative stage proteins detected using UOK77 had been observed in the previously published slug stage interactome, which identified 18 interactors (14). This suggested that the interactome of Skp1 is dynamic with respect to developmental progression. To assess the generality of this interpretation, the interactome of slug stage Skp1-myc was examined. Thirty-five interactors were identified at a threshold of 1% FDR (Table S2). Of the total of 35 candidate interactors, six were shared with the UOK77 slug Skp1 interactome (Fig. 4*E*). As for vegetative cells, the largely nonoverlapping sets suggested separate subpopulations of slug Skp1 complexes captured by the orthogonal methods. This may reflect differential epitope accessibility owing to structural differences among Skp1 complexes and is consistent with the finding that 20% to 40% of all Skp1 was uncaptured by either method.

Of the 35 slug Skp1-myc interactors, only 17 were also detected in the vegetative cell Skp1-myc interactome (Table S2), paralleling the dynamic developmental regulation observed for the UOK77 captured subpopulation. Combining the anti-myc and UOK77 slug datasets, a total of 48 Skp1 interactors were described in slug cells at the single time point analyzed, and their relative representation inferred from peptides detected are summarized in Fig. S2*B*. Overall, the co-IP approaches together predict 92 Skp1 interactors. The majority of interactors were exclusive to one stage or the other (Fig. 4*A*). Given the long half-life (14 h) of *Dictyostelium* Skp1 during development (27), these changes likely involve exchange of Skp1 partners, potentially facilitated by chaperone-based mechanisms.

### Sequence characteristics of Skp1 interactors

The interacting proteins included the formerly validated FBPs FbxwD (14) and FbxwA (28), which contain WD40 repeats that likely serve as substrate receptors, and the SCF subunits CulE, CulA, and Rbx1. The remaining hits were compared against a list of 54 proteins predicted to possess F-box like sequences based on reiterative unbiased BLASTp searches of the *D. discoideum* genome. Although these sequences were conserved in five other cellular slime molds, only seven of them appeared in the current interactome of 92 proteins (Fig. S3). The remaining hits were then examined for potential F-box sequences using an algorithm in Geneious guided by a training set of 17 predicted F-box sequences that were supported by the downstream presence of WD40- or LRR-like substrate receptor sequences (see Experimental procedures). Using these methods, 38 of the interaction candidates were predicted to possess an F-box-like sequence, if allowing for insertions between the three predicted α-helices. A heat map of the similarity among these candidates reveals a high degree of variation relative to the sequence of the F-box of FbxwA (Fig. S4*A*). To assess the significance of these differences, the heat map was recalculated using the relatively divergent F-box sequence of JcdI, validated later. As illustrated graphically in Fig. S4*B*, many of the predicted F-box sequences that were distantly related to the FbxwA F-box sequence were more closely related to the JcdI F-box sequence and *vice versa*. However, some sequences were distantly related to both, so are more speculative. The interactor sequences were also searched for canonical substrate receptor domains, including LRR, WD40 β-propeller, and ankyrin repeats, using the NCBI Conserved Domain Database and Swiss Model (see Experimental procedures). Of the 38 interactors predicted to have F-box sequences, 24 possessed substrate receptor-like domains (Fig. 4, *C* and *F*). An additional 18 interactors possessed substrate receptor-like sequences. Most predicted substrate receptor domains were LRRs, including the FNIP subclass common to *Dictyostelium* and Mimiviruses (29). Given the degeneracy of F-box domain sequences, we do not exclude the possibility that these 18 candidates may be divergent FBPs. Thus more than 60% of the vegetative and slug Skp1 interactors show sequence features of FBPs. Significantly, as tabulated in Table S2*A*, many of the 14 FBP-like proteins appear to have domains that lack substrate-receptor characteristics, consistent with the concept that some FBPs are actually independent proteins that possess F-boxes merely for regulating their turnover. Thirty-one of the remaining proteins that lack any FBP features possess sequences indicative of other functions. Whether any of these proteins possess cryptic F-box sequences or are indirectly associated with Skp1 will require future study.

### Dependence of the Skp1 interactome on PhyA

In the previous study of the interactome of slug Skp1, the representation of 3 of the 18 interactors was enhanced by the mutational deletion of enzymes that modify Skp1 (14). To address the generality of this effect, samples of *phyA*^–^ cells, which are unable to glycosylate Skp1, were examined in parallel with the new Skp1 interactomes described above. After normalization of abundances to the total peptide signal, proteins with an average abundance ratio for *phyA*^+^:*phyA*^–^ of >1.5, at a Wilcoxon *p*-value < 0.05, or with pairwise averages of *phyA*^+^:*phyA*^–^ within all biological replicates of >1.5, were considered enriched in the Skp1 interactome when Skp1 could be glycosylated. Of the 67 vegetative Skp1 interactors, 25 showed enhanced representation in WT *versus* mutant cells (Fig. 4*D*), and 14 of the 48 slug Skp1 interactors were similarly enhanced (Fig. 4*G*). In contrast, only two interactors were significantly enhanced (>1.5) in mutant cells and only in the anti-myc vegetative cell co-IP (Table S2). Overall, 29% to 37% of Skp1 interactors from either co-IP methods (mAb 9E10 or pAb UOK77) and from either developmental stage exhibited increased representation when Skp1 was modified. A high proportion (80–93%), in both stages, possessed putative F-box or candidate substrate-binding domains (Fig. 4, *D* and *G*), suggesting a selective enrichment of FBPs. Two candidates, a WD40 domain containing F-box protein (FbxwD), whose representation was enhanced by Skp1 glycosylation in slug cells, and the JmjC-containing FBP JcdI, whose representation was enhanced in vegetative cells, were selected for further analysis.

### Glycosylation dependence of the FbxwD/Skp1 interaction

FbxwD is an example of substrate receptor type of FBP whose representation in the Skp1 interactome was enhanced by PhyA in slug stage cells (Fig. 4 and Table S2). Since increased representation did not achieve significance in vegetative cells, it was unclear whether this was due to differential binding or to increased levels of FbxwD. A previous study suggested differential binding (14), but this was based on overexpression in prespore cells, which is not the cell type in which FbxwD is normally expressed. To verify this finding, FbxwD was epitope tagged *via* modification of its genetic locus to examine the interaction of endogenous FbxwD with Skp1.

**Figure 5:**
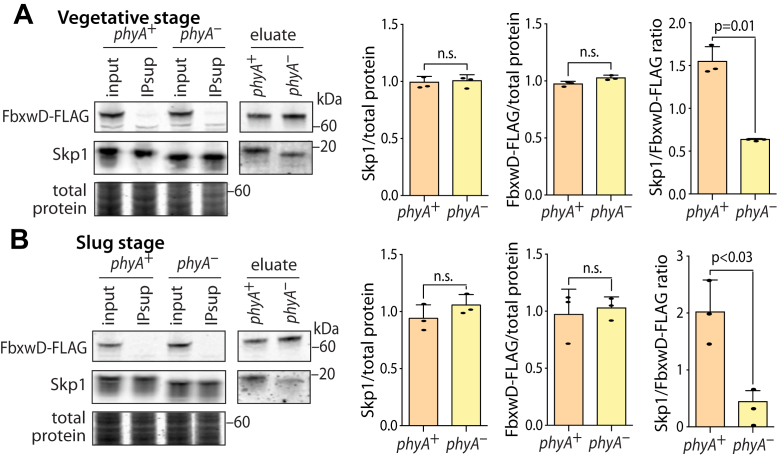
**Interactions of FbxwD with Skp1.** The *fbxwD* locus was modified in *phyA*^+^ and *phyA*^–^ cells to append a C-terminal FLAG tag to the protein. *A,* FbxwD and Skp1 abundance were analyzed by Western blot analysis of whole cells solubilized in SDS using anti-FLAG for FbxwD-FLAG and pAb UOK77 for Skp1. Levels were quantitated by densitometry and normalized to Coomassie blue staining of the blotted gel. Note the more rapid gel migration of Skp1 from *phyA*^*–*^ cells owing to absence of glycosylation. The average ratio of three independent trials ± SD are graphed. FbxwD-FLAG was IPed with mAb M2 under conditions where >90% of FbxwD-FLAG was captured and analyzed similarly by Western blotting. The average ratio of co-IPed Skp1 relative to FbxwD-FLAG is graphed from three independent trials ± SD. *B,* similar analysis of FbxwD-FLAG and Skp1 from slug cells.

A double-crossover homologous recombination strategy was employed to insert complementary DNA (cDNA) for a C-terminal peptide extension that contains a triple-FLAG epitope followed by a floxed blasticidin-resistance cassette, which was subsequently excised by transient expression of Cre-recombinase, and confirmed by PCR (Fig. S5). To examine the role of Skp1 modification, the *phyA* locus was subsequently disrupted as previously described (30). Western blotting with anti-FLAG Ab detected a novel band with an apparent *M*_*r*_ 70,000 in both strains (Fig. 5*A*), consistent with an expected *M*_*r*_ 68,430 (including the appended peptide). Tagging of the *fbxwD* locus did not have an apparent effect on growth rates in axenic medium or the morphology and timing of the developmental program.

Densitometry analysis of Western blots of whole cells indicated that FbxwD-FLAG was expressed at similar levels in *phyA*^+^ (Ax3) and *phyA*^*–*^ cells of either vegetative (Fig. 5*A*) or slug cells (Fig. 5*B*). This showed that increased representation in the Skp1 interactome of *phyA*^*+*^ cells was not simply due to higher levels of FbxwD. Consistent with a previous study (27), the absence of PhyA also did not affect levels of Skp1 (Fig. 5). To verify the increased interaction between Skp1 and FbxwD in Skp1 co-IPs, the reciprocal co-IP was performed using anti-FLAG under conditions where essentially all FbxwD-FLAG was captured. Fourfold greater levels of Skp1 were recovered in *phyA*^+^ *versus phyA*^*–*^ slug cells (Fig. 5*B*), consistent with previous findings when FLAG-FbxwD was overexpressed in prespore cells (14). Unexpectedly, a lesser 2.5-fold increased representation of Skp1 was also observed in vegetative cells. The discrepancy between anti-Skp1 and anti-FbxwD co-IPs may be due to the differential efficiency of target capture. Since anti-FbxwD capture was greater than 90% *versus* ∼60% efficiency for anti-Skp1, the results suggest that differential representation resided in the subpopulation of Skp1 that was not captured by anti-Skp1. Given that recombinantly expressed FBPs are generally insoluble in the absence of Skp1, these findings suggest that, in *phyA*^*–*^ cells, FbxwD is partially associated with alternative protein and unable to enter an SCF complex.

### Glycosylation dependence of the JcdI interaction with Skp1

**Figure 6:**
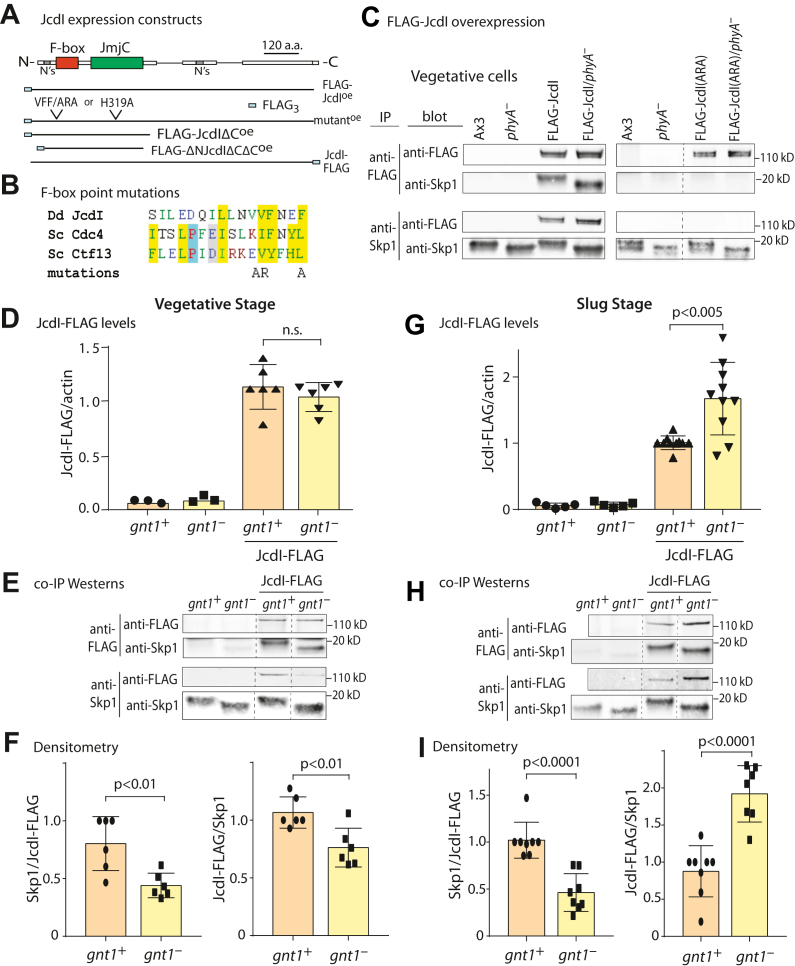
**Effect of Skp1 glycosylation on endogenous levels of JcdI and its interaction with Skp1.***A,* schematic diagrams illustrating ectopic expression of full-length FLAG-JcdI (FLAG-Fbxo13) and truncated and mutated derivatives, and tagging of the endogenous gene with a C-terminal FLAG tag (JcdI-FLAG). *B,* point mutations in the predicted F-box domain, based on mutations used for yeast Ctf13. *C,* strains ectopically expressing FLAG-JcdI and FLAG-JcdI(ARA) were subjected to co-IP with mAb M2 (for FLAG-JcdI) or pAb UOK77 (for Skp1) and analyzed by Western blotting and probing with the respective Abs. *D–F,* endogenous JcdI-FLAG was compared between *gnt1*^*+*^ and *gnt1*^*–*^ vegetative cells. *D,* the total level of JcdI-FLAG relative to actin in NP40-solubilized whole cells (panel *D*) was determined on two independent clones from three biological replicates (each represented by a triangle) using an unpaired *t* test. *E,* the interaction of JcdI-FLAG with Skp1 was assessed in reciprocal co-IPs. *F,* ratios were quantified by densitometry and the average ± SD for the indicated number of independent trials (represented by separate symbols). *G–I,* similar analyses of slug stage cells. Tooled vertical lines mark where irrelevant lanes were deleted from the same Western blot or gel. co-IP, coimmunoprecipitation.

**Figure 8:**
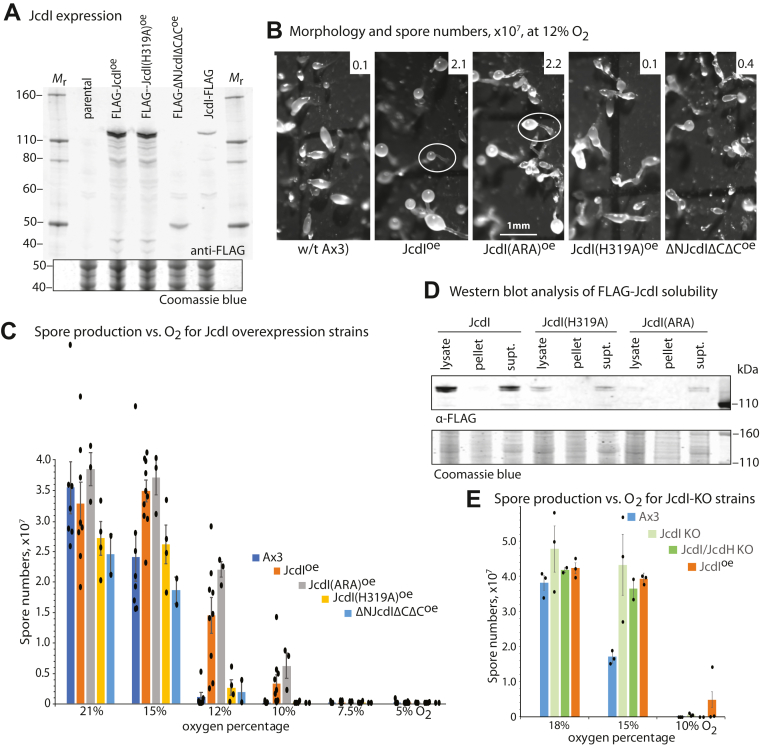
**JcdI overexpression reduces O**_**2**_**threshold for development.***Dictyostelium* was transformed with plasmids directing the overexpression of normal or mutant FLAG-JcdI isoforms under control of the constitutive discoidin-1γ promoter. *A,* clones were screened for expression level in vegetative cells, as assessed by Western blotting of whole cell extracts probed with anti-FLAG (mAb M2). Cells were induced to develop by spreading at high density on moist filters in starvation buffer as in Figure 2*B* and maintained under controlled flow of different O_2_ levels. *B,* representative terminal morphologies at 12% O_2_. Typical fruiting bodies are encircled in white. *C,* mean spore counts from three independent trials of FLAG-JcdI overexpression development, which included data from three independent FLAG-JcdI overexpression strains, after 36 h, ± SEM. *D,* Western blot analysis of the distribution of FLAG-JcdI isoforms between particulate and cytosolic fractions after gentle filter lysis of vegetative stage cells in the absence of detergent and centrifugation at 200,000*g* for 30 min. *E,* mean spore counts from three independent trials of *jcdI*^–^ and *jcdI*^–^/*jcdH*^–^ cells after 36 h, ± SEM.

JcdI is an example of a potential FBP whose representation in the Skp1 interactome is not apparently affected by PhyA (Table S2) and in fact based on previous data (14) was suspected of having enhanced representation in *phyA*^*–*^ slug cells. JcdI is a highly abundant interactor of Skp1 based on Skp1 co-IPs (Fig. S2), and the interaction is predicted to be direct due to the presence of an F-box like sequence near its N terminus (Fig. 6, *A* and *B*). To confirm this, the JcdI coding sequence was modified to append an N-terminal 3×FLAG tag and with point mutations that abrogated binding of the FBP Ctf13, a yeast centrosomal protein, to yeast Skp1 (31). FLAG-JcdI and FLAG-JcdI(ARA), in which the motif VFxxF was changed to ARxxA, were overexpressed in *phyA*^+^ and *phyA*^–^ cells using an integrating plasmid (Fig. S6). Clones expressed a novel FLAG-tagged protein that migrated as a closely spaced doublet with an apparent *M*_*r*_ of 120,000 based on SDS-PAGE (Fig. 6*E*), consistent with the predicted *M*_*r*_ of 122,000 of FLAG-JcdI. The biochemical basis for the doublet and minor anti-FLAG reactive lower *M*_*r*_ species (Fig. 8*A*) is not known. Clones that stably expressed similar levels of normal or mutant FLAG-JcdI were examined using nonionic detergent lysates in which JcdI was fully solubilized. As shown in Figure 6*C*, substantial co-IP of FLAG-JcdI was observed in anti-Skp1 pulldowns and of Skp1 in anti-FLAG pulldowns. In contrast, negligible interactions of Skp1 were observed with FLAG-JcdI(ARA). Furthermore, as described later, recombinant expression of a soluble form of JcdI in *Escherichia coli* required coexpression with Skp1, and the two proteins formed a stable complex during chromatographic purification. Thus, the JcdI/Skp1 interaction appears to be directly mediated by the predicted F-box domain in JcdI, which is now also annotated as Fbxo13 owing to its lack of an obvious substrate receptor domain.

To further analyze the interaction of JcdI with Skp1, its locus was modified to encode a C-terminal FLAG tag as aforementioned for FbxwD (Fig. S11), in *gnt1*^+^ (Ax3) and *gnt1*^–^ cells (27). *gnt1*^–^ cells were used in place of *phyA*^–^ cells owing to an incompatibility in selection markers. Though the *gnt1*^–^ strain allows for 4(*trans*)-hydroxylation of its Pro143 residue, it does not allow for glycosylation thought to be important for affecting FBP interactions (14). These tagged clones grew and developed normally and expressed a novel FLAG-tagged protein at the expected *M*_*r*_ position of 120,000 (Fig. 6*E*) that did not, however, migrate as a doublet as occurred for the overexpressed version.

The interaction between JcdI-FLAG and Skp1 was revisited using Western blot analysis of co-IP experiments, which allowed for a larger number of replicates. Based on densitometry of Western blots, analysis of unbound fractions showed that essentially all JcdI-FLAG was captured, compared to ∼75% of Skp1 that was captured by anti-Skp1 beads. In JcdI co-IPs from vegetative cells, a 2-fold greater amount of Skp1 (Skp1:JcdI ratio) was captured in *gnt1*^+^ relative to *gnt1*^–^ cells (Fig. 6, *E* and *F*). A similar though lesser difference was observed in reciprocal anti-Skp1 co-IPs, despite similar JcdI-FLAG protein levels. A similar analysis of slug cell extracts using anti-FLAG also indicated preferential (2-fold) interaction of JcdI in *gnt1*^+^ *versus gnt1*^–^ cells (Fig. 6,*H* and *I*). Remarkably, the opposite result was observed in the anti-Skp1 co-IPs. However, this can be explained by the 50% reduction in JcdI-FLAG levels in the *gnt1*^+^ slug cells (Fig. 6*G*). Thus JcdI-FLAG appears to bind glycosylated Skp1 better than unglycosylated Skp1 at both stages, as best revealed by the Skp1:JcdI-FLAG ratio in the anti-FLAG co-IPs. Since recombinant JcdI is insoluble in the absence of Skp1 (see later), JcdI appears to partially replace Skp1 with another unknown protein in *gnt1*^–^ cells, as concluded for FbxwD before. Unlike FbxwD, however, this is associated with increased relative levels of JcdI-FLAG though only in slug cells, inviting speculation that reduced involvement in SCF complexes protects it from degradation.

### JcdI is a nonheme dioxygenase

**Figure 7:**
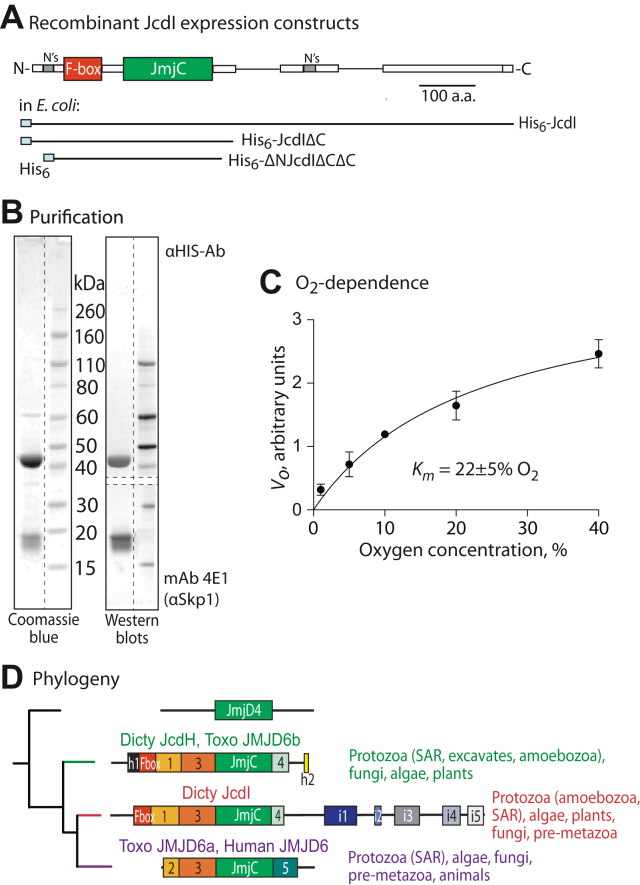
**JcdI/Fbxo13 is an O**_**2**_**-dependent nonheme dioxygenase.***A,* domain analysis of the predicted JcdI protein and summary of expression constructs in *E. coli*. *B,* SDS-PAGE and Western blot analysis of the purified ΔNJcdIΔCΔC/Skp1 complex expressed in *E. coli*. Vertical dashed lines refer to irrelevant lanes removed from the same gel, and horizontal dashed lines indicate the division of the same blot for probing with different Abs. *C,* nonheme dioxygenase activity was measured based on the conversion of αKG to succinate, as is typical of this enzyme class even in the absence of an acceptor substrate, in the presence of various levels of O_2_. Succinate was detected using a commercial luminescence-based succinate-Glo assay. Initial velocities (*V*_*o*_*)* were calculated from three technical replicates of a single trial that was replicated with similar results in an independent trial. *D,* a summary of the evolution of JcdI-like sequences, based on the relatedness of their JmjC domains using a maximum likelihood method. Members of each group have characteristic distinct sequence homology segments that are represented as shaded boxes. Phylogenetic distributions are summarized to the right. SAR, a group including stramenopiles, alveolates, and rhizaria; Dicty, *Dictyostelium* (an amoebozoan); Toxo, *Toxoplasma* (an alveolate). The full tree of 71 sequences is shown in Fig. S8.

JcdI/Fbxo13 contains a JmjC-like sequence C-terminally to its F-box domain (Fig. 7*A*). JmjC domains are a subgroup of the family of nonheme dioxygenase domains and generally hydroxylate the side chains of proteins or their methylated derivatives resulting in demethylation. To verify its enzyme activity, a C-terminally truncated isoform that retained the JmjC sequence was expressed in *E. coli* with or without coexpressed *Dictyostelium* Skp1A. Initial studies showed that coexpression with Skp1 was necessary to recover soluble protein, and further truncations of a poorly conserved N-terminal region and additional C-terminal regions were necessary to prevent degradation during expression. Purification using Ni^++^-Sepharose and gel filtration generated a nearly homogenous preparation of a complex of the truncated His_6_-ΔNJcdIΔCΔC with Skp1 (Fig. 7*B*), confirming the direct and stable association of JcdI with Skp1. Since the target of JcdI is unknown, a luminescence assay for free-wheeling conversion of the cosubstrate α-ketoglutarate (αKG) to succinate and CO_2_, as commonly occurs for this enzyme class, was employed. Pilot studies indicated a pH optimum of 6.0 to 7.0, with markedly decreased activity at pH 7.5 and above, and maximal activity at 10 μM αKG. Thus, the peripheral domains were not required for this core activity. Under optimized conditions (see Experimental procedures) where activity was linearly dependent on time and enzyme concentration, the *K*_*m*_ for O_2_ was estimated at 22 ± 5% O_2_ (Fig. 7*C*), suggesting that, in addition to regulating JcdI *via* PhyA, O_2_ may also directly control JcdI’s enzymatic activity.

### JcdI/Fbxo13 is evolutionarily related to human JMJD6

BLASTp searches at NCBI for sequences similar to the JcdI catalytic domain yielded JmjD6 as the most related group of proteins and JmjD4 as the second most related. A manually curated alignment of the amino acid sequences of the 71 most similar JmjC domains across phylogeny (Fig. S7) was analyzed by a maximum likelihood method to explore the diversity within this group. As indicated by the unrooted tree shown in Fig. S8 and summarized in Figure 7*D*, JmjD6-like sequences are found throughout the eukaryotic kingdoms of protists, plants, fungi, and animals and resolved into three major groups that were well resolved from the JmjD4-like sequences. Human JmjD6 and all of the animal sequences are clustered in a subclade of a larger group (at bottom, in *violet*) that also includes representatives from major protist groups, including the stramenopile-alveolate-rhizaria (SAR) cluster, algae, and fungi. These sequences are related approximately according to the phylogeny of the organisms consistent with a common function. This is supported by the presence of two characteristic sequence motifs, referred to as homology domains H2 and H5, that flank the JmjC domain and the adjacent H3 homology domain that is found with all JmjD6 and JmjD4 JmjC domains. The other two groups each contain representatives from a diverse array of protists, higher plants, and some fungi, but not metazoa. They are distinctive from the animal JmjD6-like sequences in that their H3/JmjC domain pair is flanked by homology domains H1 and H4. Strikingly, they are each in possession of an F-box-like sequence near their N termini, a position that is typical for FBPs. JcdI lies in the second group (in middle, *red*), whereas the closely related *Dictyostelium* paralog, JcdH, lies in the third group (*green*). The members of each of these two groups are related approximately according to the phylogenies of their parent organisms, consistent with a common function, which is supported by their additional shared domains as indicated. Members of the JcdI group have characteristic homology sequences i1-i5 at their C termini, which are lacking from the JcdH group. Many but not all members of the JcdH group have sequence homologies referred to as Hh1 and Hh2 (Fig. S8). The alignment of JmjC domains also reveals sequence motifs that are distinctive among the three groups (asterisks in Fig. S7*A*). The topology of the tree suggests that modern day human JmjD6 originated and diversified in protists *via* two successive gene duplications. In this scenario, the JcdI and JcdH groups evolved from the duplication of an ancestral JmjD6-like gene containing H1 and H4. Then, a second gene duplication spawned the animal and human JmjD6 sequences and was associated with the replacement of H1 and H4 by H2 and H5, respectively. Some members of the JcdH and JcdI groups subsequently acquired additional characteristic domains. Thus, it is unclear whether JcdI and JcdH, with their distinctive F-box domains, have the lysyl hydroxylase or arginine demethylase activities that have been controversially ascribed to human JmjD6 (10, 15, 16). To our knowledge, Fbxo13/JcdI represents the first FBP found to be conserved across the protist kingdom, which includes many pathogens of animals (*e.g.*, *T. gondii*) and plants.

### Developmental role of JcdI in O_2_ sensing

The finding that JcdI’s *in vitro* enzyme activity is limited by physiological levels of O_2_ led us to genetically test its contribution to O_2_ sensing in cells. We previously found that overexpression of PhyA reduces the O_2_ threshold required for culmination, as assayed morphologically and by quantifying the number of spores that differentiate in the resulting fruiting bodies (18). Here, we analyzed a strain that was genetically altered to constitutively overexpress FLAG-JcdI (Fig. 8*A*). In these trials and based on spore numbers generated, parental cells (strain Ax3) required more than 15% O_2_ to fully culminate, and negligible culmination occurred at 12% O_2_ (Fig. 8*C*). In contrast, approximately 50% of FLAG-JcdI overexpressing cells still culminated at 12% O_2_ and 10% culminated at 10% O_2_. Similar results were obtained for two independent FLAG-JcdI overexpression clones. Representative images of the morphology of the terminal structures confirm the failure of to culminate into fruiting bodies at 12% O_2_, whereas the FLAG-JcdI overexpression strain exhibited substantial fruiting body formation (Fig. 8*B*). To assess whether this effect depended on JcdI enzymatic activity, an inactivating point mutant, in which the highly conserved His319 residue critical for binding the cosubstrate αKG (32, 33, 34) was changed to Ala, was overexpressed at similar levels (Fig. 8*A*). This strain required 15% O_2_ for efficient culmination and exhibited minimal culmination at 12% O_2_. To examine the significance of the modestly conserved C-terminal region of the JcdI clade of JmjD6-like sequences, a truncated version, FLAG-ΔNJcdIΔCΔC (Fig. 6*A*), was analyzed in a clone that overexpressed the protein. This strain was similar to the parental Ax3 strain (Fig. 8, *B* and *C*), suggesting that the C-terminal domain contributes to O_2_-dependent JcdI functionality; however, clones expressing the same high level of expression were not found (Fig. 8*A*). Finally, the importance of the F-box domain was tested using FLAG-JcdI(ARA). Interestingly, this overexpression strain, which does not bind Skp1 to a significant extent in co-IPs (Fig. 6*C*), also required less O_2_ to culminate, like native JcdI. Since JcdI required coexpression with Skp1 for solubility when expressed in *E. coli*, the FLAG-JcdI overexpression strains were subjected to gentle filter lysis under conditions that preserve organelles and centrifuged at high *g* force to generate particulate and cytosolic fractions. Unexpectedly, FLAG-JcdI(ARA), like the WT isoform, was primarily soluble (Fig. 8*D*) and, thus, potentially functional as an enzyme.

The contribution of JcdI to O_2_ sensing was also examined in a strain in which its coding region was replaced with a *bsr* cassette. This *jcdI*^–^ strain did not exhibit statistically significant effects of O_2_ levels on spore production (Fig. 8*E*). Since the genome encodes a related JmjC-containing FBP, JcdH (Fig. 7*D*), we examined a strain whose *jcdH* locus was disrupted. This strain also exhibited no effect on O_2_-dependent spore production (data not shown). Finally, we generated a *jcdI*^–^/*jcdH*^–^ strain, which also exhibited normal O_2_ dependence. These findings suggest that genetic redundancy, possibly by another of the 12 annotated JmjC proteins encoded by the *Dictyostelium* genome, may compensate for the absence of these proteins. In contrast, the dominant effect of JcdI’s overexpression suggests a functional role in O_2_ sensing that would be expected to be concealed by redundant functions.

## Discussion

Cells have a need to sense and respond to changing levels of O_2_ in their environments, and this is especially important for single-celled protists that are not buffered by multicellularity. Here, we find that a relative of human JmjD6 contributes to O_2_ sensing in *Dictyostelium*, a protist that uses gradients of O_2_ for aerotaxis (35) and, during development, to polarize its slug (36, 37) and to know to culminate near the soil surface (18). *Dictyostelium* JcdI is a nonheme dioxygenase that uses O_2_ as the source of O atoms to hydroxylate residues on currently undefined target proteins. JcdI is also an FBP, which renders it subject to regulation by another nonheme dioxygenase, the prolyl hydroxylase PhyA, *via* its action on the Skp1 subunit of the SCF class of E3(SCF)Ub-ligases. Prolyl hydroxylation of Skp1 leads to an increased fraction of JcdI that is bound to Skp1, which is associated with decreased cellular levels of JcdI during development. Our finding that the Skp1 association of another FBP, FbxwD, is also increased by PhyA suggests that a similar mechanism may operate for others of the 90 additional and largely dynamic Skp1 interactors suggested by this study. The role of PhyA in regulating Skp1 appears to involve protein degradation owing to rescue of the culmination defect of *phyA*^–^ cells by proteasomal inhibitors.

### JcdI has properties of an oxygen sensor during development

JcdI was selected for analysis not only because of its presence in the interactome of Skp1, which is regulated by the PhyA O_2_ sensor, but also because its possession of a putative JmjC domain suggests that it too directly depends on O_2_. We found that recombinantly expressed JcdI is indeed a nonheme dioxygenase, and its apparent *K*_*m*_ (O_2_) of 22% (Fig. 7) indicates that its enzymatic activity would be directly limited by O_2_ availability in cells. Genetically induced overexpression of JcdI modestly reduced the O_2_ requirement for culmination (Fig. 8), as expected if its activity was rate limited by O_2_ and this was overcome by the presence of more enzyme. The inability of a similarly expressed inactive mutant confirmed that JcdI acted *via* its enzymatic activity to render this effect. A truncated version of JcdI that retained its JmjC and adjacent domains was also not active, suggesting that the missing domains contribute in some way to JcdI function. Thus JcdI is similar to PhyA in the sense that its overexpression reduced the O_2_ requirement for culmination but presumably acts in a parallel pathway as Skp1 is not modified in *phyA*^–^ cells (30). However, its removal by gene replacement did not increase the O_2_ requirement as occurs in *phyA*^*–*^ strains (18). *Dictyostelium* expresses a closely related homolog, JcdH, but neither its disruption nor the double KO of *jcdI* and *jcdH* had any effect on the O_2_ dependence of culmination (Fig. 8). Thus, JcdI may function as a go-signal to coordinate a parallel aspect of O_2_ sensing, whose absence might therefore lie beyond the sensitivity of our analyses. Alternatively, JcdI might function redundantly with another of the 12 predicted JmjC-containing proteins encoded by *Dictyostelium*. The expression pattern of JcdI, which according to a global transcriptomic analysis is expressed at a 2.7-fold higher level in prestalk over prespore cells (38), is consistent with action in the apical end of the slug thought to signal culmination (18).

### Evolution of JcdI

Based on sequence similarities, JcdI is predicted to be a lysyl hydroxylase as directly demonstrated for human JmjD6, and/or possibly an arginine demethylase which has also been proposed (16, 39). However, an analysis of the evolution of the JmjC domains of JmjD6-like sequences suggests that JcdI evolved separately from JmjD6 after a gene duplication early in eukaryotic evolution (Figs. 7*D* and S8), followed by diversification of domains that flank the JmjC domain and persist today in extant versions in protists, fungi, and metazoans. Thus, these paralogs may have a distinct catalytic function. *Dictyostelium* JcdH is found in another lineage of JmjD6-like proteins that appears to have derived from a yet earlier gene duplication, and this group lacks the C-terminal sequences that characterize the JcdI group. The JcdI and JcdH families are distributed widely in protists, algae, and fungi but are conspicuously absent from metazoa. They also all contain a predicted F-box domain, rendering them potentially subject to another level of regulation *via* the SCF complex and PhyA.

### Effect of PhyA on JcdI and FbxwD

The potential for regulation of JcdI by the SCF was suggested by its presence in the Skp1 interactome and its predicted F-box domain. JcdI is confirmed to be an FBP because point mutations in its predicted F-box domain inhibited its interaction with Skp1 (Fig. 6*C*). Furthermore, coexpression with Skp1 in *E. coli* was required for its solubility and a stable complex was formed (Fig. 7). The original co-IP studies suggested a modestly greater representation in the Skp1 interactome of *phyA*^–^ slugs, in contrast with other interactors. A similar result was obtained by Western blot analysis of strains in which endogenous JcdI was FLAG-tagged in *gnt1*^+^ and *gnt1*^–^ cells (Fig. 6*I*), though this difference was not observed in vegetative cells (Fig. 6*F*). More telling was that in reciprocal co-IPs captured with anti-FLAG, approximately 2-fold more Skp1 (ratioed to JcdI-FLAG) was captured from *gnt1*^+^ compared to *gnt1*^–^ cells in both slug and vegetative cells. The discrepancy is explained by the finding that endogenous JcdI-FLAG was reduced 2-fold in *gnt1*^+^ compared to *gnt1*^–^ slug but not vegetative cells. The result from the anti-FLAG co-IP is considered more reliable owing to is capture of >95% of JcdI-FLAG from extracts, compared to the <75% of Skp1 captured by anti-Skp1, which would allow for bias if subpopulations vary in epitope accessibility. Since recombinant JcdI is insoluble in the absence of Skp1, it is likely that in *gnt1*^–^ cells, JcdI is bound to an alternative protein, as has been observed for other FBPs (40, 41, 42), and would not be accessible to the SCF and possible autoubiquitination and degradation. An example of this scenario was described for the FBP Tir1 in *Arabidopsis* (43). This explanation for why JcdI-FLAG levels are reduced in *gnt1*^+^ cells is consistent with the ability of overexpressed mutant FLAG-JcdI(ARA) to affect O_2_ sensing, if the substitution of the very hydrophobic Val and two Phe residues with an Arg and two Ala residues were sufficient to render it soluble, as suggested by the cell fractionation results (Fig. 8*D*). While the physiological role of PhyA-dependent reduction of JcdI levels is not known, one possibility is that it is a slow homeostatic process to regulate the rapid stimulatory effect of O_2_ on JcdI enzymatic activity.

A second FBP, FbxwD, also exhibited preferential binding to Skp1 when it was glycosylated, though no statistically significant effects on its levels were observed (Fig. 5). Unlike FbxwD, JcdI is an enzyme and potentially not a traditional substrate receptor, which might subject it to differential outcomes.

Regulation of FBP/Skp1 interactions has also been described in yeast in response to stress (*e.g.*, heavy metals) or Skp1 phosphorylation (44, 45). Skp1 glycosylation represents a novel mechanism. Previous studies showed that glycosylation inhibits Skp1 dimerization (21) and since dimerization interferes with FBP binding (46), glycosylation could increase the availability of Skp1 for FBP interactions. In addition, glycosylation also modifies the conformational profile of the F-box binding region of Skp1 in a manner that is predicted to increase accessibility to F-box domains (47), which was supported by size-exclusion chromatography of Skp1 complexes from normal compared to mutant extracts (14), and enhanced binding to mammalian Fbs1 *in vitro* (48). Evidence also exists for this mechanism in other protists including *T. gondii* and *P. ultimum* (8, 13, 49).

### How general is the effect of Skp1 modification on FBPs?

The current study expands our knowledge of the putative Skp1 interactome by use of an epitope-tagged Skp1 in addition to an affinity purified Skp1 antiserum and by analyzing its developmental regulation (Fig. 4 and Table S2). In addition to three known non-FBP SCF subunits, 89 proteins were specifically associated with Skp1 at a high confidence level, which is a 5-fold increase over our previous study of slug stage cells using anti-Skp1 (50). The proteins in the interactome range in abundance by over 500-fold (Fig. S2); 16% lack known substrate receptor or other domains (Table S2). Only 14% of the proteins were detected by both pulldown methods, suggesting that epitope accessibility varied among complexes and confirming the value of orthogonal capture agents for these complexes. About 73% of the interactors were observed in vegetative cells, and 52% were in slug cells, with only 25% apparently present throughout the life cycle. To address the possibility that presence simply reflected the availability of interactors at the two stages, we queried the transcriptomes of vegetative and slug cells as reported (51). Superimposing values for reads per kilobase of transcript per million mapped reads above protein abundance values interpreted from the proteomics data showed no systematic correlations (Fig. S2), suggesting that presence in the interactome is based on selectivity rather than simple availability. Using a 4-fold reduction in transcript level as an approximate prediction of what difference might on average result in loss of detection in one stage relative to the other, we found that less than 40% of the vegetative-only interactors and less than 30% of the slug-only interactors met this criterion (Table S2). Furthermore, two vegetative-only proteins had higher transcript expression in slug cells and *vice versa* for 16% of the slug-only proteins. Given the long half-life of Skp1 in cells (27), which undergo minimal proliferation during development, it is thus likely that changes in the Skp1 interactome involve some developmental reassortment of Skp1 interactors.

Many of the Skp1 interactors appear to be FBPs and may bind Skp1 directly. Thirty-eight possess F-box-like sequences in their N-terminal regions based on sequence and/or structural homology. Twenty-four of those possess C-terminally oriented sequences that represent known substrate receptor domains, and 18 additional proteins possess potential substrate receptor domains, raising the possibility that they also possess cryptic F-box domains that explain their association with Skp1. These approaches likely underestimate the number of FBPs, given the extensive variation within known F-box sequences including insertions that make the motif difficult to detect. In addition, a BLASTp search for F-box-like sequences that are conserved among social amoebae yielded 38 additional candidates (Fig. S4) that were not detected in this study, which might have been due to epitope inaccessibility, low expression level, or expression at other life cycle stages. The remaining proteins that are not predicted to be FBPs might be FBP substrates or cofactors that contribute to substrate interactions (52) or possibly reflect unknown functions of Skp1 in this protist. Skp1 interactors described in other organisms, such as Sgt1 (53), were not observed despite the presence of a highly conserved homolog in the *Dictyostelium* genome.

The representation of ∼39% of the interactors were increased when Skp1 was glycosylated in either of the two biological states investigated, whereas, strikingly, only two were decreased (in vegetative cells). The affected proteins were enriched in FBPs relative to all interactors (80% *versus* 92%) (Fig. 4, *A*–*G*). Possible explanations for increased representation are increased absolute levels available for co-IP, colocalization of interactor and Skp1 pools in cells, different interaction stability, or an increased fraction of the FBP pool that is stably bound to Skp1 as shown for FbxwD and Skp1 (Figs. 5 and 6). This latter effect may be more common than inferred based on the example of JcdI where decreased levels concealed increased interaction (Fig. 6). Analysis of an alignment of F-box-like sequences of interactors did not reveal obvious correlations with PhyA dependence, but a role for Skp1-interacting sequences outside of the canonical F-box domain cannot be excluded.

### The role of protein degradation

The dynamic nature of the Skp1 interactome suggests that the specificity of protein degradation changes over the life cycle, a concept that has been previously proposed (28). O_2_ sensing occurs not only for regulating slug migration and polarization (37), culmination (18), and sporulation (36) but also for aerotaxis at the single cell vegetative stage (35). The role of Skp1 modification in vegetative cells is unknown. The very low conservation of FBP sequences across phylogeny makes it difficult to predict substrates so experimental approaches will be key to confirming this prediction. Nevertheless, the ability of proteasomal inhibitors to partially rescue the development of *phyA*^–^ cells (Fig. 2) indicates that PhyA senses O_2_ *via* an effect on E3(SCF)Ub ligase activity, which is consistent with its evident specificity for Skp1 (13, 54, 55). A possible explanation is that proteasomal inhibition raises the level of FBPs, which would have the effect of driving increased interactions with Skp1 toward levels normally achieved *via* Skp1 glycosylation. Although proteasomal inhibition would also be expected to interfere with the action of substrate receptor type FBPs, FBPs like JcdI and JcdH with SCF independent activities might experience increased levels and promote developmental progression. However, which FBP(s) is responsible for mediating PhyA’s role in culmination remains to be determined. Overall, the findings support the general model that, for the soil-dwelling *Dictyostelium*, Skp1 modification is a mechanism that links the pace of protein turnover required for developmental progression to favorable environmental factors like optimal O_2_ levels. This phenomenon is reminiscent of the role of protein turnover in regulation of developmental timing during mammalian development (56).

## Experimental procedures

### Cell culture

*D. discoideum* strains Ax3, Ax4, and their genetically modified derivatives (Table 1) were routinely grown vegetatively in shaking axenic HL-5 media at 22 °C and passaged by 1000-fold dilution every third or fourth day. Strains Ax3 and Ax4 are considered normal but are mutant derivatives of a WT strain. Vegetative stage cells were collected by centrifugation during logarithmic growth for experimental studies. Starvation-induced development was induced on at an air–water interface on filters or in suspension as previously described (36). Slug stage cells were typically scraped at 14 h unless otherwise indicated. Cell density was determined using a hemacytometer.

**Table 1:** Strains used in this study

Strains	Parental	Genotype	Resistance	Ref.
Ax3	NC-4	*axeA/B/C*	none	(84)
Ax4	Ax3	*axeA/B/C*	none	(64)
HW288	Ax3	*phyA*^–^	blasticidin-S	(30)
HW302	Ax3	*dscC*::Skp1B-myc	G418	(25)
HW304	HW302	*dscC*::Skp1B-myc; *phyA*^–^	G418, blasticidin-S	this report
HW503	Ax3	*gnt1*^*–*^	hygromycin	(27)
HW418	Ax3	*dscC*::His_6_AgtA	G418	(23)
HW535	Ax3	*dscC*::FLAG-AgtA	G418	this report
HW536	HW288	*dscC*::FLAG-AgtA; *phyA*^–^	G418, blasticidin-S	this report
HW540	Ax3	FbxwD-FLAG	blasticidin-S	(14)
HW544	HW288	FbxwD-FLAG; *phyA*^–^	blasticidin-S	(14)
HW559	Ax3	*dscC*::FLAG-JcdI (Fbxo13)	G418	this report
HW560	Ax3	*dscC*::FLAG-JcdI	G418	this report
HW561	Ax3	*dscC*::FLAG-JcdI	G418	this report
HW562	Ax3	*dscC*::FLAG-JcdI(ARA)	G418	this report
HW564	Ax3	*dscC*::FLAG-JcdI(H319 A)	G418	this report
HW566	Ax3	*dscC*::FLAG-JcdI(ΔNΔCΔC)	G418	this report
HW568	HW288	*dscC*::FLAG-JcdI; *phyA*^–^	G418, blasticidin-S	this report
HW570	Ax3	JcdI-FLAG	blasticidin-S	this report
HW572	HW503	JcdI-FLAG; *gnt1*^*–*^	blasticidin-S, hygromycin	this report
HW574	Ax3	*jcdI*^–^	blasticidin-S	this report
HW576	Ax3	*jcdI*^–^	none	this report
HW578	HW576	*jcdI*^–^/*jcdH*^–^	blasticidin-S	this report
37_C_10	Ax4	*jcdH*^–^	blasticidin-S	(85) (GWDI)

The dependence of culmination on O_2_ levels was performed by incubating filter dishes in air-tight translucent containers, through which was continuously passed humidified premixed gasses consisting of the indicated O_2_ level with the balance made up of N_2_, as described (27). Plates were illuminated by overhead fluorescent (daylight) room lighting at 22 °C. Morphology was qualitatively assessed by direct visualization during development and after 38 h. Culmination was quantitated by direct spore counting in a hemacytometer. Over the years and possibly the seasons, the O_2_ requirement for culmination of the parental strain Ax3 in the laboratory varies from 7.5% to 15% O_2_, suggesting sensing of unknown factors. For the trials conducted for this project, the average O_2_ requirement was 15%. Each trial included all experimental and reference strains to control for this variation and were carried out over a period of several months.

### Proteasome inhibitor treatments

Proteasome inhibitors (Ubiquitin-Proteasome Biotechnologies) were dissolved in anhydrous dimethyl sulfoxide (DMSO) and used fresh or from frozen aliquots stored at −80 °C. Stock solutions were 30 mM MG132, 30 mM bortezomib, and 50 mM carfilzomib. For suspension development, cells were resuspended in Agg buffer (0.01 M sodium phosphate, pH 6.0, 0.01 M KCl, 0.005 M MgCl_2_) containing the indicated concentration of inhibitor or the equivalent concentration of DMSO and, after shaking for the indicated times, were recovered by centrifugation and flash frozen. For development at an air–water interface, cells were resuspended in PDF (33 mM NaH__2__PO__4__, 10.6 mM Na__2__HPO__4__, 20 mM KCl, 6 mM MgSO__4__, pH 5.8), deposited on filters wetted in the same inhibitor solution or the equivalent volume of DMSO and, after incubation for the indicated times, scraped and flash frozen. Because the occasional breakthrough culmination of *phyA*^–^ cells at ambient (21%) O_2_, as previously reported (18), would confound interpretation of inhibitor effects, only data from the trials (16/20) that failed to culminate at 21% O_2_ were analyzed.

### SDS-PAGE and Western blotting

For whole cell analysis, fresh or frozen cell pellets were diluted with Laemmli sample buffer and boiled for 5 min. For analysis of cytosolic fractions, cells were filter-lysed (57) in 0.25 M sucrose in 50 mM Tris–HCl (pH 8.0) plus protease inhibitors and centrifuged at 200,000*g* for 30 min. Supernatants were diluted 1:1 and boiled for 5 min in 2× Laemmli buffer and pellets resuspended in Laemmli buffer as aforementioned. Samples (2–10 × 10^5^ cell equivalents) were separated on precast 4% to 12% Bis–Tris NuPage polyacrylamide SDS page gels (Invitrogen) for 45 min in MOPS or MES (for Skp1 analysis) running buffer. Proteins were transferred to nitrocellulose using an iBlot 2 (Invitrogen) dry blotting system. Blots were blocked in 5% (w/v) nonfat dry milk in Tris-buffered saline (100 mM NaCl, 50 mM Tris–HCl, pH 7.5), probed with the indicated primary antibody in blocking buffer overnight at 22 °C, washed in Tris-buffered saline, probed again with a corresponding Alexa Fluor-680 or Alexa Fluor-800 secondary Ab (1:10,000 dilution) in blocking buffer, and quantitated for fluorescence in a Li-Cor Odyssey infrared scanner. Blotted gels were stained with Coomassie blue and scanned for use as a protein loading control. Blots were alternatively postincubated with antiactin (Sigma) as a loading control. Digital image files were processed uniformly in Adobe Photoshop by expanding to use the full 8 bit range without altering contrast. Scans were densitometrically analyzed in ImageJ (imagej.NIH.gov/ij/) with appropriate background subtraction.

For detection of polyubiquitinated proteins, blots were probed with 1:700 mouse anti-Ub antibody (P4D1, Cell Signaling Technology), 1:1000 rabbit monoclonal K48-linked Ub-specific antibody (D9D5, Cell Signaling Technology), or 1:1000 rabbit monoclonal K63-linked Ub-specific antibody (D7A11, Cell Signaling) (58), and processed with an appropriate species-specific Alexa Fluor-conjugated secondary antibody as aforementioned. mAb M2 (Sigma) was used at 1:1000 for probing FLAG-tagged proteins, and mouse mAb 4E1 or rabbit pAb UOK77 (14) were used for Skp1.

### Co-IPs

For MS, co-IPs of Skp1 using affinity purified pAb UOK77 and a nonimmune rabbit IgG were performed as described (14). For co-IPs of Skp1-myc and corresponding untagged strains, cell pellets were solubilized in lysis buffer (250 mM NaCl, 50 mM Tris–HCl, pH 8.0, 0.2% v/v NP-40, 10 μg/ml leupeptin, 10 μg/ml aprotinin) at a final concentration of 1.2 to 1.5 × 10^5^ cells/μl for vegetative cells and 2.4 × 10^5^ cells/μl for slugs for 15 min on ice. Cell lysates were spun at 21,000*g* for 15 min to produce an S21 supernatant. Eighty-three microliters was incubated with a 100 μl slurry of Pierce anti-c-*myc* magnetic polystyrene beads (#88842), a ratio that was optimized for the minimal amount that achieved maximal (>60%) depletion of Skp1-myc from lysates. After rotation at 4 °C for 1 h, beads were magnetically captured and the supernatant removed. The beads were successively rinsed 1× in lysis buffer, 2× in lysis buffer without protease inhibitors, 3× in detergent buffer (250 mM NaCl, 50 mM Tris–HCl, pH 8.0), and finally in detergent and buffer free solution (50 mM NaCl). Proteins were eluted in 133 mM triethanolamine for 15 min, neutralized immediately with acetic acid, and dried in a vacuum centrifuge for MS.

For Western blot analysis of JcdI/Skp1 interactions, 3 × 10^7^ slugs or exponentially growing vegetative cells were lysed as aforementioned. Skp1 was immunoprecipitated using 5 μl of packed affinity purified UOK77 conjugated to Sepharose beads (14). FLAG-JcdI and JcdI-FLAG were immunoprecipitated using 2.5 μl of packed anti-FLAG M2 beads (Sigma). After rotation for 1 h at 4 °C, beads were collected by centrifugation at 2000*g* for 30 s, resuspended and washed 3× with 1 ml of IP buffer (50 mM Tris–HCl, pH 8.0, 150 mM NaCl, 0.2% NP-40), and transferred to a fresh tube after the first centrifugation. The preparation was boiled in 1× Laemmli buffer for 3 min, and 1 × 10^6^ cell equivalents were analyzed by SDS−PAGE and Western blotting.

For Western blot analysis of FbxwD co-IPs, endogenously tagged FbxwD-FLAG and untagged control strains were lysed as aforementioned to produce S21 fractions. One hundred microliters were incubated with 10 μl packed M2 magnetic beads (Sigma) by rotation at 4 °C for 1 h. Beads were washed as aforementioned and eluted with Laemmli buffer for SDS-PAGE and Western blotting.

### Proteomic analyses

Eluted proteins were solubilized in 8 M urea in 50 mM Tris–HCl (pH 7.8), reduced with 10 mM DTT at 22 °C, and alkylated with 50 mM chloroacetamide (59). After 1:1 dilution with 50 mM Tris–HCl (pH 7.8), 1 μg of endo-LysC/trypsin (Pierce; catalog no.: #A40009) was added and rotated at 22 °C for 2 h and then subsequently diluted with the same buffer to a final concentration of 2 M urea for continued overnight digestion.

The peptide samples were amended with heptafluorobutyric acid to a final concentration of 0.1% (v/v) and aspirated five times through a pre-equilibrated C18 Zip-Tip (Agilent Bond Elut Omix), which was then rinsed twice with 0.1% heptafluorobutyric acid. Peptides were eluted with 50% (v/v) acetonitrile (ACN), 0.1% formic acid, followed by 75% ACN, 0.1% formic acid. The eluents were pooled, dried in a vacuum centrifuge, and dissolved in 5% ACN, 0.05% TFA.

The peptide solution was loaded onto a C18 trap column (Thermo Acclaim PepMap 100 C18 series) in a Thermo Fisher UltiMate 3000 nano-HPLC and eluted from the trap column onto a C18 nano-column (Thermo Acclaim PepMap 100 C18 series) in a 5% to 90% ACN gradient in 0.1% formic acid over 3 h. The eluent was directly introduced *via* a nanoelectrospray source into a Thermo-Fisher Q Exactive Plus and analyzed by MS and MS/MS. Full MS scans were acquired from *m*/*z* 350 to 2000 at 70,000 resolution. Peptides were selected for fragmentation in the C-trap *via* higher energy collision-induced dissociation for MS/MS analysis using a Top 10 method and a 30 s fragmentation exclusion window.

Samples were analyzed in Proteome Discoverer 2.5, using its two step protein search method with label-free quantification and Consensus workflow with parameters specific to the MS1 and MS2 fragmentation and mass tolerances of the Thermo-Fisher Q Exactive Plus. A modified *D. discoideum* protein database containing 12,428 unique proteins (60), modified to include a 179 protein exclusion list of common ectopic contaminants (38), was used for peptide identification. Sequest HT search parameters were 10 ppm precursor ion mass tolerance, 0.02 Da fragment ion tolerance, and up to two missed tryptic cleavages; variable modifications: oxidation of Met, formylation or acetylation of the protein N terminus; fixed modification: carbamidomethylation of Cys. FDR was determined *via* Target/Decoy in the Proteome Discoverer Processing Workflow. Protein identifications were ranked by protein FDR confidence intervals of high (X < 1%), medium (1% < X < 5%), and low (5% < X < 10%). Candidates assigned as mitochondrial, ribosomal, or secretory proteins (see listing in database referenced below) were filtered as before (14). Protein quantifications were derived from reconstructed ion chromatograms of all peptides assigned to a protein at the MS1 level. Comparisons of protein abundance were done after setting total abundance of each sample within a single analysis equal to the highest value within the experiment in Proteome Discoverer. Normalized abundance values of proteins were analyzed for statistically significant differences between groups using the SimpliFi algorithm (https://simplifi.protifi.com/) (61). Since SimpliFi can use nonparametric statistics that assume only that data model themselves, we used this to perform tests on normalized abundances, with 1 added to all spectral counts to avoid zero values, rather than on logarithmic transformations. Proteins whose abundances were >4-fold higher in experimental *versus* control samples with a Wilcoxon test *p*-value <0.05 were classified as Skp1 interactors. Proteins whose values were ≥1.5-fold higher in *phyA*^+^ *versus phyA*^–^ samples, with *t* test and Wilcoxon test *p*-values <0.05, were classified as enriched in the *phyA*^+^ Skp1 interactome. The MS proteomics data (listed in Table S3) are deposited in the ProteomeXchange Consortium *via* the PRIDE (62) partner repository with the dataset identifier PXD033864 and 10.6019/PXD033864.

### Sequence motif searches

To predict the FBP repertoire of *D. discoideum*, BLASTp studies (63) were conducted at dictybase.org using multiple validated human and yeast (6) F-box sequences to generate a candidate list. This list was expanded by additional BLASTp searches seeded with candidate *Dictyostelium* F-box sequences to ensure maximal coverage. Additional inclusion criteria were evidence for expression based on RNA-seq data (64), predicted targeting to the cytoplasm or nucleus, positioning of the F-box motif N-terminal to other domains of interest, and manual confirmation of conservation of hydrophobic residues and helical motifs with proven F-box motifs. This yielded 52 confident candidates (Fig. S4), almost all of which were conserved in the genome sequences of other cellular slime molds.

In addition, the sequences of new Skp1 interactors (Table S2) were searched using the Geneious https://www.geneious.com pairwise alignment algorithm against a training set of 17 predicted F-box domains from FBPs found above that also contained a canonical substrate receptor domain. Parameters included a PAM250 cost matrix, a gap open penalty of 20, a gap extension penalty of 10, and two refinement iterations. In some examples of F-boxes, such as Ctf13, Amn1, Ylr352w, and Ydr306c of *Saccharomyces cerevisiae* (*e.g.*, 71), intervening sequences occur between α-helices. Thus, manual refinement of the alignments tolerated loops between α-helix 1 and α-helix 2, sequence extensions between the C terminus of α-helix 3 and the final 4 to 5 amino acids of the F-box domains but not gaps/extensions within α-helices or between α-helix 2 and 3. All predicted F-box sequences were aligned with the training set using the same parameters (excluding inserts) (Fig. S4*A*), and Percent pairwise similarity was used to generate heat maps (Fig. S4*B*).

To search for potential substrate receptor domains, the full-length amino acid sequences of Skp1 interactors were input into Swiss Model’s (65, 66, 67, 68, 69, 70) web interface for homology modeling. Proteins containing more than 500 amino acids were divided into multiple amino acid segments to reduce complexity.

### FLAG-AgtA overexpression strains

AgtA cDNA was excised from the previously described pVS(His)spy-AgtA (71) using BamHI and ligated into the BamHI site of the previously described (14) pVS3 (pV3D) expression plasmid, which derives from pVS4 and contains a sequence downstream of the semiconstitutive discoidin 1γ promoter encoding the following motifs: ATG/BclI/His_6_/BglII/3 × FLAG/EcoRI/BirA recognition/NcoI/TEV protease site/BamHI/TAA. A clone containing the desired forward orientation of AgtA, termed pVS3-FLAGAgtA, was confirmed by DNA sequencing. pVS3-FLAGAgtA was electroporated into Ax3 and *phyA*^–^ strains, and stable transformants were selected in the presence of 20 or 120 μg/ml G418, as described (72). Clones from each transformation were grown and screened for FLAG-AgtA expression levels using anti-FLAG (M2) and anti-AgtA (pAb UOK101), enabling selection of clones with equivalent levels of overexpression in growth stage cells.

### Tagging the fbxwD and jcdI loci

C-terminal tagging of the FbxwD gene product was based on double crossover homologous recombination (73). The *fbwxD* 5′-targeting sequence was amplified from *D. discoideum* genomic DNA (gDNA) (Ax3 strain) by a PCR using primers FbxwD-5′-S and FbxwD-5′-AS (Table S1) and ligated into pCR4-TOPO (Invitrogen). The *fbxwD* 3′-targeting sequence was amplified and ligated in a similar manner using FbxwD-3′-S and FbxwD-3′-AS. The *fbxwD* 5′-targeting sequence was excised using BssHII and BglII and cloned into similarly digested pVSCulE-BsR, which was used previously for tagging the *culE* locus (14). The *fbwxD* 3′-targeting sequence was similarly transferred using PstI and PvuII. The *fbwxD*-tagging construct was excised using *PvuII* and *BssHII*, briefly treated with BAL-31 exonuclease (72), and gel purified. WT strain Ax3 cells were transformed by electroporation as previously described (71), selected in 10 μg/ml blasticidin-S (MP Biomedicals) in HL-5+ medium, and cloned on *Klebsiella aerogenes* on SM-agar. Cell lysates from clones were initially analyzed for modification of the *fbxwD* locus by Western blotting using the M2 anti-FLAG mAb (Sigma) and verified by PCR (Fig. S5). To examine the role of Skp1 modification, the floxed *bsr* cassette used to select for tagged *fbxwD* transformants was excised by transient transfection with the puts-NLS Cre plasmid encoding Cre recombinase as before (73). Successfully deleted clones were screened based on sensitivity to blasticidin and confirmed by PCR (Fig. S5). The *phyA* locus was subsequently disrupted as previously described (30).

JcdI was tagged in a similar manner (see Fig. S11). The primer pair JET-5′-S and JET-5′-AS (Table S1) was used to amplify the 3′-region (996 bp) of *jcdI* from gDNA by PCR, and a 915 bp stretch downstream of the JcdI stop codon was amplified using JET-3′-S and JET-3′-AS. The PCR products were separately cloned into pCR4-TOPO and sequences confirmed using M13F and M13R primers. The 5′-targeting insert was recovered from pCR4-TOPO-JET-5′ by digestion with BssHII and BglII and cloned into similarly digested pVSCulE-BsR. pCR4-TOPO-JET-3′ was then digested with PstI and PvuII and the insert cloned into similarly digested pVSCulE3′-JET5′-BsR. The 4 kb tagging cassette was excised using BssHII and PvuII and electroporated into strains Ax3 (*gnt1*^+^) and HW503 (*gnt1*^*–*^). Cells were selected in the presence of 10 μg/ml blasticidin-S and clones recovered from bacterial plates were extracted for gDNA and screened by PCR, using JET-UP-S, designed to anneal just upstream of the 5′-targeting sequence, and the reverse primer JET-BSR-AS designed to anneal in the bsr cassette of the construct. PCR was performed as aforementioned with a denaturation temperature of 94 °C for 45 s, annealing temperature of 56 °C for 1 min, and extension temperature of 68 °C for 2 min, repeated for 30 cycles (Fig. S11).

### Expression of JcdI in *E. coli*

The coding sequence for JcdI from *D. discoideum* (DDB_G0270006 at dictyBase.org; Q55CL5 at UniProtKB) was codon optimized for *E. coli* expression, modified to include silent restriction sites to facilitate domain deletions, and synthesized and inserted into pUC57 by GenScript (Fig. S9). pUC57-JcdI was digested with NdeI and BglII to release the coding region for a C-terminally truncated fragment of JcdI that retains its F-box and catalytic domains and ligated into pET15TEVi that was digested with NdeI and BamHI. This resulted in the addition of an N-terminal His_6_-tag; the construct was referred to as His_6_-JcdIΔC. Transformation of *E. coli* resulted in expression of substantial amounts full-length protein, which was nearly all insoluble.

A Skp1A expression cassette (T7 promoter + cDNA) was amplified by PCR using p48-Skp1-for-dual-S and p48-Skp1-for-dual-AS (Table S1) from pET19b-Skp1A and ligated into the SpeI site of pET15TEV-JcdIΔC. The correct orientation of the insert was confirmed by dual digestion with XbaI and HindIII and agarose gel electrophoresis. Induction of *E. coli* transformed with this dual expression plasmid yielded a soluble His_6_-JcdIΔC/Skp1 complex (see later), but breakdown products were observed on Coomassie brilliant blue gels and anti-His_6_ Western blots. To allow future coexpression with other proteins, this double expression cassette was transferred from the pET15 backbone (*ampR*) to pET24 backbone (*kanR*) using BglII and HindIII.

To overcome apparent instability, His_6_JcdIΔC was subjected to further truncations. To remove the N-terminal amino acids 2 to 42 (123 nucleotides), which included a poorly conserved poly-N tract, two primers, NtermJCDIdel-S and NtermJCDIdel-AS, that bridged the interval to be deleted, with nine nucleotides on either side, and off-set from each other so that the 3′-end of each primer did not anneal to the 5′-end of the other, were used to amplify the deletion construct by PCR. PCR conditions were as follows: 2U Q5 DNA polymerase (NEB), 1 μM each primer, 0.2 mM dNTPs, plasmid template, and 30 cycles with 70 °C annealing temperature and 4 min extension time. The amplification product was treated with DpnI, reacted with T4 DNAse at 22 °C for 1 to 2 min to create sticky ends, incubated at 75 °C for 20 min, and allowed to self-anneal for 30 min at 55 °C or 65 °C. The final product was transformed into *E. coli* TOP 10 cells yielding, after sequence confirmation, pET28a-ΔNJcdIΔC/Skp1A. Next, the C-terminal coding region was further truncated by 60 aa using the same method, using JCDI-N-ΔN-S and JCDI-N-ΔN-AS as primers, except that DNAse treatment was not done, resulting in expression plasmid pET28a-ΔNJcdIΔCΔC/Skp1A.

This plasmid was transformed into *E. coli* strain ER2566 and protein expression was induced using 0.5 mM IPTG in 100 μg/ml ampicillin in LB. Cells were harvested by centrifugation, resuspended in ice-cold 20 mM Tris–HCl, pH 7.5, spun again, and frozen at −80 °C. The cell pellet was thawed in 20 ml/culture liter of 0.1 M Tris–HCl, pH 7.5, 5 mM benzamidine, 0.5 μg/ml pepstatin A, 10 μg/ml aprotinin, 10 μg/ml leupeptin, 1 mg/ml lysozyme, and 1 mM PMSF, subjected to Dounce homogenization with four strokes on ice, and probe-sonicated with a 1/2 inch probe, 60% amplitude, 1 s on, 3 s off, for 5 min, in an ice-water bath. The suspension was supplemented to achieve 5 mM MgCl_2_, 50 μg/ml RNaseA, and 10 μg/ml DNaseI. A supernatant was generated by centrifugation at 22,000*g* for 30 min at 4 °C and adjusted to 0.5 M NaCl and 5 mM imidazole before loading onto a pre-equilibrated 1 ml His-Trap column (Pharmacia). The column was washed with the same buffer and step eluted with increasing concentrations of imidazole in the same buffer. The majority of ΔNJcdIΔCΔC coeluted with Skp1A at 250 mM imidazole and was concentrated by centrifugal ultrafiltration and further purified over a Superdex200 16/60 column equilibrated in 50 mM Hepes-NaOH, pH 7.4, 50 mM NaCl, 10% (v/v) glycerol, 2 mM DTT, 10 μg/ml aprotinin, and 10 μg/ml leupeptin.

### JcdI enzyme activity

Reaction cocktails consisting of 100 μM α-ketoglutarate, 1 mM ascorbate, 5 μM FeSO_4_, 0. 1% Tween-20, 50 mM Tris–HCl (pH 7.5), 25 mM NaCl, 1 mM tris(2-carboxyethyl)phosphine, 0.5 M betaine, 5% (v/v) glycerol, 10 μg/ml aprotinin, and 10 μg/ml leupeptin were pre-equilibrated in 1 ml sealed conical reactivials (Pierce) through which flowed, *via* needles piercing the Teflon seal, humidified premixed gas mixtures of O_2_ and N_2_. The reaction was initiated by the introduction, *via* a gas tight syringe, of highly purified and degassed JcdI (ΔNJcdINShort + Dd-Skp1A), to a final volume of 50 μl. Following incubation at 22 °C, 10 μl was withdrawn with a syringe and divided over two wells of a white 384-well plate each containing 5 μl of freshly prepared SDR-1 (according to manufacturer’s directions for Succinate-Glo kit, Promega). Following mixing for 30 s and incubation for 1 h at 22 °C, 10 μl of SDR-II was added. After 30 s of mixing and 10 min of incubation at 22 ºC, luminescence was recorded at 1 mm height for 1 s by a Synergy H4 plate reader (Bio-Tek). Pilot studies established incubation times and protein concentrations for which activity was linearly dependent.

### Ectopic expression of JcdI in *D. discoideum*

Exon 1 of JcdI (Fig. S6) was amplified from *D. discoideum* gDNA (74) by PCR using primers JcdI-Ex1-S and JcdI-Ex1-AS, and exon 2 was similarly amplified using JcdI-Ex2-S and JcdI-Ex2-AS (Table S1). The PCR product bands were purified by electrophoresis and separately cloned into pCR4-TOPO (Invitrogen), resulting in pCR4-TOPO-JcdI-Ex1 and pCR4-TOPO-JcdI-Ex2. Their sequences were confirmed using primers M13F and M13R. Exon 2 was released using BspEI and SacI and cloned into similarly digested and Antarctic phosphatase (NEB) treated pCR4-TOPO-JcdI-Ex1. The predicted F-box domain was modified by the introduction of three point mutations by site-directed mutagenesis of pCR4-TOPO-JcdI using JcdI-SDM-FboxARA-S and JcdI-SDM-FboxARA-AS (Table S1), as previously described (18), yielding pCR4-TOPO-JcdI(ARA). The *jcdI* cDNAs were released using NcoI and SacI and cloned into similarly digested and Antarctic phosphatase treated pV3D (pVS3). The pV3D-JcdI plasmid was used as a template to generate a FLAG-JcdI(H319A) expression plasmid through site-directed mutagenesis. The FLAG-ΔNJcdIΔCΔC expression construct was achieved by PCR deletion on the pV3D JcdI plasmid with overlapping homology regions in the 5′ portion of primers (Table S1). All sequences were verified. Native pV3D-JcdI and the mutant constructs were electroporated into strains Ax3 (*phyA*^+^) or HW288 (*phyA*^–^) and transformants were selected in the presence of 10 μM G418 (71). Clones were recovered from plaques growing on lawns of *K. aerogenes* and screened by Western blotting with an anti-FLAG mAb M2. Clones expressing similar levels of FLAG-JcdI, FLAG-JcdI-ARA, and FLAG-JcdI(H319A) during vegetative growth were chosen for further analysis. High levels of FLAG-ΔNJcdIΔCΔC expression were not found. None of the stains showed proliferation defects in HL-5 medium.

### Disruption of jcdI and jcdH loci

The coding region of the *jcdI* locus was disrupted by double crossover homologous recombination as previously described (71) (Fig. S12). Briefly, a 1033 nt 5′-targeting region was amplified from the 5′-end of the coding region with JCDI-5′-S and JCDI-5′-AS, using gDNA (74) as a template, and cloned into pCR4-TOPO. An 860 nt 3′-targeting region was amplified from the middle of the coding region using JcdI-3′-S-Stop and JcdI-3′-AS and cloned into pCR4-TOPO. The 5′-targeting region was excised using BssHII and BamHI cloned into pVSCulE-BsR (see previous text) predigested with BssHII and BglII. The 3′-targeting region was next excised with PstI and PvuII and cloned into similarly digested pVS-JcdI-5′-BsR, yielding pVS-JcdI-5′-BsR-3’. This construct was designed to replace the JmjC domain with a floxed *bsr* cassette and introduced stop codons at the intersections to minimize possible read-through translation of remaining coding DNA. The *jcdI* disruption DNA was released using BssHII and PvuII and electroporated into Ax3 cells. Clones were screened by PCR using JcdI-KO-5′UpS, which anneals upstream of the 5′-targeting sequence, and JET-BSR-AS, which anneals within the introduced bsr cassette, and with JcdI-KO-3′Scr-AS, which anneals downstream of the 3′-targeting DNA, and JcdI-KO-BSR3′Scr-S, which anneals within the bsr cassette. The floxed bsr cassette was then excised as aforementioned and successfully deleted clones were screened based on sensitivity to bsr, and confirmed by PCR using primer pairs p5 and p9 and p8 and p6 (Fig. S12).

JcdH (DDB_G0280485; protein model: DDB0238368, at dictyBase.org; Q54VA7 at UniProtKB) is described in Fig. S10 and was disrupted in the *jcdI*^–^ strain as described in Fig. S12. As for *jcdI*, a 739 bp 5′-targeting DNA was PCR-amplified from gDNA using 5′-targeting forward (p1) and 5′-targeting reverse (p2) oligonucleotides (Table S1) and cloned into pCR4-TOPO. A 665 bp 3′-targeting DNA was PCR amplified from within the coding region using 3′-targeting forward (p3) and 3′-targeting reverse (p4) oligonucleotides and cloned into pCR4-TOPO. These fragments were assembled into pVSCulE-BsR as aforementioned to generate pVS-JcdH-5′-BsR-3’. This construct is designed to replace JcdH amino acids 24 to 347, which include the F-box domain and a portion of the JmjC domain, with the *bsr* cassette. PCR screening of transfected clones was performed using pVS_tag_non-c_seq_F-v2, which anneals within the bsr cassette, and JcdH3′UTRScrR(p6) or p12/JcdH-3′ext-AS, which anneal downstream from the 3′-targeting DNA.

### Phylogenetic analyses

Sequences related to *Dictyostelium* JcdI were analyzed as described previously (75). In brief, the sequence of the JmjC domain (147 amino acid residues) from XP_646466.1 was used as query for BLASTp searches against sequence databases (NCBI, DOE JGI, EUPATHDB, and Broad Institute). The 74 best hit sequences were aligned and trimmed of highly gapped insert regions. The phylogenetic tree was built using IQ-Tree (76) with the following options: -m MFP -bb 10,000 and used the LG+R5 substitution model. ModelFinder (77), implemented in IQ-Tree, was used to find the best fit model. Bootstrap support values were obtained using UltraFast bootstrap approximation (UFBoot) (78) implemented in IQ-Tree, and the tree was visualized in iTOL (79). The overall topology of the tree was robust as an independent tree generated using MEGA had a similar topology.

## Data availability

The mass spectrometry proteomics data are deposited in the ProteomeXchange Consortium *via* the PRIDE partner repository with the dataset identifier 10.6019/PXD033864.

## Supporting information

This article contains supporting information (14, 51, 80). The file contains 3 tables and 12 figures.

## Conflict of interest

The authors declare that they have no conflicts of interest with the contents of this article.
