# Supplementary material for: Oxygen-dependent regulation of E3(SCF)ubiquitin ligases and a Skp1-associated JmjD6 homolog in development of the social amoeba Dictyostelium
Source: J Biol Chem. 2022 Aug 4;298(9):102305. doi: 10.1016/j.jbc.2022.102305 (PMC9485057; doi:10.1016/j.jbc.2022.102305)

SUPPORTING INFORMATION

Oxygen-dependent regulation of E3(SCF)ubiquitin ligases and a Skp1-associated JmjD6 homolog in *Dictyostelium*

Andrew W. Boland[1,2], Elisabet Gas-Pascual[1,2,3], Braxton L. Nottingham[4], Hanke van der Wel[1,4], Nitin G. Daniel[1], M. Osman Sheikh[4], Christopher M. Schafer[4], Christopher M. West[1,2,3,4]

[1]Dept. of Biochemistry & Molecular Biology, [2]Complex Carbohydrate Research Center, [3]Center for Tropical and Emerging Global Diseases, University of Georgia, Athens, GA 30602, [4]Dept. of Biochemistry & Molecular Biology, University of Oklahoma Health Sciences Center, Oklahoma City, OK 73104 USA

Table of Contents

Table S1. Oligonucleotides employed in this study.

Table S2. Summary of Skp1 interactors.

Table S3. Database submissions.

Fig. S1. Volcano plots of proteins identified in Skp1 co-immunoprecipitates.

Fig. S2. Average abundances and scaled PSMs of proteins found in Skp1 co-immunoprecipitates.

Fig. S3. Alignment of predicted F-box domain sequences found in Skp1 interactors.

Fig. S4. Heat map of relatedness of F-box sequences.

Fig. S5. Tagging the FbxwD gene locus.

Fig. S6. Expression of FLAG-JcdI.

Fig. S7. Alignment of sequences of JmjC domains of JcdI-related proteins.

Fig. S8. Evolution of JmjD6-related sequences.

Fig. S9. JcdI nucleotide and amino acid sequences.

Fig. S10. JcdH nucleotide and amino acid sequences.

Fig. S11. Tagging the *jcdI* gene locus.

Fig. S12. Disruption of the *jcdI* and *jcdH* gene loci.

Table S1. Oligonucleotides employed in this study. Restriction sites used are *italicized*.

Primer Name	Sequence
FbxwD tagging	
FbxwD-5'-S	5'-AA*GCGCGC*GAAAAGAATGAGAATAAAGGAGCAGCA (*BssH1*)
FbxwD-5'-AS	5'-AA*CCATGG*ATCTAAATTAAAATTATATAATCTAACTAAATTATTGAAAACAC (*NcoI*)
FbxwD-3'-S	5'-AT*CTGCAG*TTGTTAAGATCAAATTACTGTGGATTTAATTG (*PstI*)
FbxwD-3'-AS	5'-TT*CAGCTG*GATGTTTTAATGGTAATTTTTTAATTGTTTCTC (*PvuII*)
FbxD3Tar-AS	5'-ATCCACAGTAATTTGATCTTAACAACTGC
BsR-AS (SKPK/O2U–AS)	5'-GAGTGGAATGAGTTCTTCAATCGTAG
BirATAG-S	5'-AGCACAAAAAATTGAATGGCATGG
JcdI expression and tagging	
JcdI-Ex1-S	5'-TAT*CCATGG*TATGGTAGTTCTTAAAAACACAT (*NcoI*)
JcdI-Ex1-AS	5'-GAA*TCCGGA*AAATGTAATGGAATATATG (*BspEI*)
JcdI-Ex2-S	5'-CATTT*TCCGGA*TTTCTATTCACATGA (*BspEI*)
JcdI-Ex2-AS	5'-*GAGCTC*TTATACATCTAAATTCCAAATTGAATT (*SacI*)
J-ET-5'S	5'-*GCGCGC*TACCAGATTCATTACCTTA (*BssH1*)
J-ET-5'AS	5'-*AGATCT*TGAGCCTGATCCTACATCTAAA (*BglII*)
J-ET-3'S	5'-*CTGCAG*ATCGCATTGCAATCTACTG (*PstI*)
J-ET-3'AS	5'-*CAGCTG*TCACTTTTTTACCAGTTTTAACTCC (*PvuII*)
JET-OUT-S (JET-UP-S)	5'-GTCATGGTCAACAGAGTTATTCTTATATTCAA
JET-BSR-AS	5'-TTCGTATAATGTATGCTATACGAAGTTATCCGT
JcdI-disruption	
JCDI-5'-S-Stop	5'-*GCGCGC*ATGGTAGTTCTTTAAAACA (*BssH1*)
JCDI-5'-AS	5'-*GGATCC*TGATGGCACTTTCTCGC (*BamHI*)
JCDI-3'-S-Stop	5'-*CTGCAG*TGTAATTCTTAAAATTTCGATGAAGT (*PstI*)
JCDI-3'-AS	5'-*CAGCTG*CAACTAAGAAATCAACTAAATTGTC (*PvuII*)
JcdI-disruption Screening	
JcdI-KO-5'UpS	5'-TGTGCATACATCATAATAATCGGTTGGAA
JET-BSR-AS	5'-TTCGTATAATGTATGCTATACGAAGTTATCCGT
JcdI-KO-BSR3'Scr-S	5'-ATATGCATTAGATGTAAAACAGCCAAAGAGTATGAA
JcdI-KO-Dwn3'Scr-AS	5'-TATCATCAATTTCAATTGAATGAATTAATGATA
JcdI-KO-BSR-AS	5'-ACAATTGATGGACGACCCGAGCT
JcdI-F-box point mutation	
JCDI-SDM-FboxARA-S	5'-ATCAATTTTAGAGGATCAAATTTTATTGAACGTTGCTAGAAATGAAGCAACATGTAGTGAACTATTGA
JCDI-SDM-FboxARA-AS	5'-AACACATTGATATTTCAATAGTTCACTACATGTTGCTTCATTTCTAGCAACGTTCAATAAAATTTGAT
JcdI-H319A point mutation	
JcdI-H319A-S	5'-CTTCGTGGGCGATCGACCCTGCCG
JcdI-H319A-AS	5'-GGGTCGATCGCCCACGAAGCACCG
ΔN-JcdI Nshort Dicty expression	
JcdI-ΔN-S	5'-CATGGTATGTCAAAAGGTGTCAAAGTTGTAATTGGAGAAAC

JcdI-ΔN-AS	5'-ACCTTTTGACATACCATGGATATGCCATTCAATTTTTTGTG
JcdI-ΔC-S	5'-TCTCAAAATTAAGAGCTCGGGTCgtccatcaattg
JcdI-ΔC-AS	5'-GAGCTCTTAATTTTGAGAATTACAAAAGTTTTGAGTTACTGCAATC
JcdH-disruption	
JcdH-5′-KO-S	5'-*GCGCGC*GCTAATGAACCATCAAAATGTGAAAT (*BssHI*)
JcdH-5′-KO-AS	5'-*GGATCC*CACCAAATGGATGTGGAATATATAATTCAAC (*BamHI*)
JcdH-3′-KO-S	5'-*CTGCAG*CCACCACATGTTGTACCACCAGG (*PstI*)
JcdH-3′-KO-AS	5'-*GTCGAC*CTTAAATCATCACCAAAAGAGAATGAAAATG (*PvuII*)
JcdH-disruption validation	
JcdH-del-S1	5'-ATAATTGATTCAATTTTAGATTTATTAAGTGCAGAAGAATTAAC
JcdH-del-S2	5'-CAATGAATTTAGTTAGTAAAACATTTTATATTTATGTTCAAGAGG
JcdH-del-AS1	5'-AATACATTGAATAAATCCTCTTTGAAATAAGCATCTG
JcdH-del-AS2	5'-GTTTGGATCTTTATGAAATGAAGCACCTG
BSR_pVS_conf_v3	5'-GACCCGAGCTCTGATCATTAGGAT
pVS_tag_non-c_seq_F-v2	5'-CCGAAAGCTCGGATCTGATATCATAACTTC
JcdH3′ext-AS	5′-AGTCGTACTTACTGCTGCCGC

Table S2A, B. Summary of Skp1 interactors. *A*, the average fold-enrichment of proteins, based on the sum of their peptides quantitated in co-IPs with Skp1 or Skp1-myc, relative to control co-IPs, are listed for those with an enrichment ratio of greater than 4 at p-values (t-test or Wilcoxon) of <0.05. Statistical significance is based on 3 biological replicates with up to three technical replicates each. Sequest scores and FDR values (depending on data processing method) indicate confidence of protein assignments. Interactors that satisfied the above criteria, but fell within the category of common background/IP artifact proteins, were excluded from the list of interactors (See Methods). Data from experiments that detected a protein whose identification and enrichment were statistically significant using a different antibody, but were not independently significant, are shaded in gray. Skp1 interactor candidates are grouped from top to bottom according to their detection at the vegetative stage, both stages, or slug stage only. Within groups, candidates are clustered according to method of detection and generally from highest to lowest confidence (top to bottom). Columns to the right-hand indicate the predicted presence of F-box, substrate receptor, and other domains based on sequence analysis and structure modeling. The right-hand columns indicate transcript abundance at the vegetative and slug stages from ref. 51. See Fig. S1 for 2-D volcano plots illustrating the distribution of enrichment ratios and statistical significance relative to control co-IPs, and Fig. S2 for graphing of statistically significant interactors according to relative abundance.
B, The relative enrichments of proteins in w/t (*phyA*$^+$) vs. *phyA*$^-$ strains are listed, and those whose enrichment is ≥1.5 with statistical significance of p<0.05 are shaded in green. Additionally, candidates enriched 1.5-fold in w/t co-IPs in pairwise comparisons of w/t vs. *phyA*$^-$ in all three biological reps are shaded in green. See Fig. 4 for volcano plots for enrichment in *phyA*$^+$ vs. *phyA*$^-$ cells.

Table S3. PRIDE submission table of contents
Dataset identifiers PXD033864 and 10.6019/PXD033864

	file name
MS summary charts	Skp1-myc_veg_proteinsA.xlsx
	Skp1-myc_veg_peptidesA.xlsx
	Skp1-myc_slug_proteinsA.xlsx
	Skp1-myc_slug_peptidesA.xlsx
	UOK77 veg IP PSMs.xls
	Dictyostelium non-specific binders.xlsx

	IP date	.RAW file name
veg	20-09-11	AX3_myc_IP_10ul_24Sep20
		HW302_1_myc_IP_10uL_1
		HW302_2_myc_IP_10uL_2part1
		HW302_2_myc_IP_10uL_2part2
		PhyA_myc_IP_10ul_25Sep20
		HW302_1_PhyA_myc_IP_10ul_25Sep20
		HW302_2_PhyA_myc_IP_10ul_25Sep20
	22-03-02	Ax3_2uL_04Mar22_1
		Ax3_2uL_04Mar22_2
		Ax3_2uL_04Mar22_3
		HW302_2uL_04Mar22_1
		HW302_2uL_04Mar22_2
		HW302_2uL_04Mar22_3
		PhyA_2uL_07Mar22_1
		PhyA_2uL_07Mar22_2
		PhyA_2uL_07Mar22_3
		PhyA_HW302_rep1_3uL_22Mar22_1
		PhyA_HW302_rep1_3uL_22Mar22_2
		PhyA_HW302_rep1_3uL_22Mar22_3
	22-03-27	AX3_1uL_28Mar22_1
		AX3_1uL_29Mar22_2
		AX3_1uL_29Mar22_3
		AX3_HW302_2uL_30Mar22_1
		AX3_HW302_2uL_30Mar22_2
		AX3_HW302_2uL_1Apr22_3
		PhyA_1uL_1Apr22_1
		PhyA_1uL_1Apr22_2
		PhyA_1uL_1Apr22_3
		PhyA_HW302_test_2uL_4Apr22_1
		PhyA_HW302_test_2uL_4Apr22_2
		PhyA_HW302_test_2uL_4Apr22_3

	IP date	.RAW file name
slug	20-02-10	AX3_slug_10uL_11Feb20_1
		AX3_slug_10uL_11Feb20_2
		AX3_slug_10uL_11Feb20_3
		AX3_HW302_myc_slug_10uL_13Feb20_1

		AX3_HW302_myc_slug_10uL_13Feb20_2
		AX3_HW302_myc_slug_10uL_13Feb20_3
		PhyA_myc_slug_10uL_13Feb20_1
		PhyA_myc_slug_10uL_13Feb20_2
		PhyA_myc_slug_10uL_13Feb20_3
		PhyA_HW302_slug_10uL_13Feb20_1
		PhyA_HW302_slug_10uL_13Feb20_2
		PhyA_HW302_slug_10uL_13Feb20_3
		AX3_slugs_myc_IP_12ul_28Sep20
		HW302_1_slugs_myc_IP_12ul_28Sep20
		HW302_2_slugs_myc_IP_12ul_28Sep20
		PhyA_slugs_myc_IP_12ul_30Sep20
		HW302_1_PhyA_slugs_myc_IP_12ul_30Sep20
	20-09-20	HW302_2_PhyA_slugs_myc_IP_12ul_30Sep20
		AX3_slugs_5uL_07Apr22_1
		AX3_slugs_7uL_07Apr22_2
		AX3_slugs_5uL_08Apr22_3
		HW302_slugs_5uL_08Apr22_1
		HW302_slugs_5uL_08Apr22_2
		HW302_slugs_5uL_08Apr22_3
		PhyA_slugs_test_5uL_09Apr22_1
		PhyA_slugs_test_5uL_11Apr22_2
		PhyA_slugs_test_5uL_11Apr22_3
		PhyA_HW302_slugs_test_5uL_12Apr22_1
		PhyA_HW302_slugs_test_5uL_12Apr22_2
	22-04-06	PhyA_HW302_slugs_test_5uL_12Apr22_3

veg	results files	22_04_06Skp1vegAll.mzid
	search	22_04_06Skp1vegAll.msf
		22_04_06Skp1vegAll.pep
	mzXML	22_04_06Skp1vegAll.prot
	mzML	22_04_06Skp1vegAll.mzML

slug	results files	220413_AllSkp1Slug.mzid
	search	220413_AllSkp1Slug.msf
		220413_AllSkp1Slug.pep
	mzXML	220413_AllSkp1Slug.prot
	mzML	220413_AllSkp1Slug.mzML

Fig. S1. Volcano plots of protein abundance ratios from experimental vs. control co-IPs, against statistical significance.

A, average abundance values from reconstructed chromatograms from Proteome Discoverer 2.5 analyses of vegetative stage samples co-IPed with mAb 9E10 (anti-myc) from cells expressing Skp1-myc vs. controls were used to determine fold enhancement of proteins identified at an FDR <1%. These values were plotted vs. statistical significance based on pooled $phyA^+$ and $phyA^-$ samples (6 biological replicates × 3 technical replicates each). Thresholds (red bars) were set at an enrichment ratio of 4 at t-test and Wilcoxon test p-values <0.05. Red symbols represent proteins that satisfy these criteria, and those with predicted F-box domains are represented as red asterisks. Gray dots represent proteins that were excluded from further analysis owing to their common high abundance in control pull-downs. 42 proteins were assigned as Skp1 interactors.

B, same as in panel A, except that the ratio of total spectral counts from co-IPs of control vegetative cells using pAb UOK77 (anti-Skp1) or non-immune rabbit IgG (3 biological replicates × 3 technical replicates each) were analyzed. 32 proteins were identified in this group.

C, analysis of slug stage co-IPs using mAb 9E10 as in panel A. 35 proteins identified with an FDR <1% were detected in this group.

D, re-analysis of previously published (14) dataset of slug stage co-IP using pAb UOK77 at FDR <1%, collected as in panel B, with the current analysis protocol yielding 18 proteins.

Fig. S2. Average abundances of Skp1 interactors.

Proteins that satisfied criteria as Skp1 interactors (see Table S2 and Fig. S1) are quantified. *A,* average abundance values of proteins derived from reconstructed chromatograms of peptides from pooled *phyA*$^{+}$ and *phyA*$^{-}$ mAb 9E10 co-IPs from vegetative stage cells (6 biological replicates × 3 technical replicates each) are graphed in blue. Average scaled spectral counts pooled from *phyA*$^{+}$ and *phyA*$^{-}$ pAb UOK77 co-IPs from vegetative cells (3 biological replicates × 3 technical replicates each) were scaled to the mAb 9E10 values based on the average values for proteins detected in both samples, and are graphed in orange. Left and right panels show higher and lower abundance proteins. Protein candidate names are bolded, italicized or colored based on predicted characteristics as shown in the key. Candidates enhanced in *phyA*$^{+}$ samples are marked by an asterisk (see Fig. 4). RPKM values (51) at 0 h of development, equivalent to vegetative cells, are graphed in gray and after 18 h (slug stage) of development are graphed in yellow.

B, same for slug-stage samples (6 biological replicates × 3 technical replicates each for mAb 9E10; data from (14) for UOK77.

Fig. S3. Alignment of predicted F-box domain sequences found in Skp1 interactors. An alignment of 83 F-box sequences predicted based on global BLASTp studies (class b) or from sequences of Skp1 interactors (this study) searched using a Geneious pair-wise alignment algorithm relative to a training set of 17 confident F-box domain sequences (class p or t) (See Methods for details). Loops and inserts were removed in the alignment shown but are noted below. To facilitate visualization of relatedness, acidic residues are in blue, basic in dark red, Gly and Pro in red, and hydrophobic in green, as previously described (80). Positions possessing related chemical characteristics are highlighted in yellow (hydrophobic), gray (acidic), dark grey (basic), or teal (small). Residues identical to the consensus sequences are bolded. Sequences are ordered from high to low similarity to FbxwA. See Fig. S4 for a similarity matrix.

Gene name[§]	Class[‡]	F-box-like sequence with loops removed	SR/other	PhyA enhanced
Geneious Consensus		INILPEEILLKILSYL-DLNDLLNLSLV-CKXWRKIISBN----LWKNL		
>*fbxA*	abtm	FDNLPEEVVQIIFSNL-SAINIVNLSLV-CKRFKMATDSP---ILWKNL	WD40	slug
>DDB_G0268696	a	LPNFPEEIKLQIFSHL-SASDLVSISLT-CKTYYAIANERT---LWRNL	Kelch	
>DDB_G0281237	abt	IYGLPMEMLFEILSNL-SVVDLVKVSKV-SKFFYSVVNNTS--ISFENM	LRR	
>DDB_G0285445	abt	FDLLPYEMIQYIFTLM--DATHLIRMSR-TCKYFNRICLDDN--IWRDL	WD40	
>DDB_G0290127	abts	FDNLPSEVIEKIFSYLSFEYDIYRVSLV-CNYWNQIAKSN---DIWLNI	WD40	slug
>DDB_G0269442	abt	FQLLPLEVMVMIFENL-FHDVHYCVSLV-CRQWHSYTFADS---IWKNL	WD40	
>DDB_G0285411	ab	MEDLPTETLILIFKNL-KINELLNVGLV-SRFFFLVSSDD---RLWKLL	Ankyrin	
>DDB_G0291902	ab	CEELPYDILLYILSYL-TPIELCCLSRV-CYSFRVLAEDD---WIWRGF		
>*gefG*	ab	FLDLDEKIYLKIFGYL-FAEDLCSINRV-SKHLCNIINNQQ---LWKDL	RasGEF	
>DDB_G0280581	at	LFLLPNEIISNILSIL-EVKDIVALSIV-NNHFYYLINSYNN--LWKNL	WD40	
>*aarA*	ab	IFLIPTEMLVHLLSFL-SANDLWRISLT-CKRIWYIVDVF---KFWELL	ARM	
>DDB_G0291716	ab	FEELPLEIIVYILGFLECEDIGLRTSLL-NKNFRDISDFY---FIWKNL	WD40	
>DDB_G0293826	ab	IGSVPREVWIHILSFL-NERDLLNISMV-DSFLNEISQDN---TLWKPL		
>*rliC*	ab	SNILSKEIQSLIVLNL-SIYDLLSLFRV-NKYWYEFGQNN---SFWKSL		
>DDB_G0278443	b	LEVLKPELVNYIFLKV-GALDMTSLIGV-NKQCYRIGMNE---KLWLYF	ARF	
>DDB_G0275777	ab	ISDLPYEVLCKILSFL-DVNTLISTSLV-SNLFNKISNSN---EIWKPK		
>DDB_G0280895	abt	LFYLPHEIIMTILSYL--DHRALCKVSR-TCKYMKQMSESD--ILWYRL	WD40	
>DDB_G0289099	ab	ILELPDDILLMIFRYL-DVPTLLKVSQL-CNFLNSVSSNH---ELWKEL		
>DDB_G0268536	abt	ILKLPLELLEKIFKFL-EQKSLCSTSLV-NKQWNHTVN-----LVWHTI	LRR	
>DDB_G0292834	abt	ISILPIEITMKIISYL-PFQDVLSIQYV-CSEWFLITCEE---ILWKSI	WD40	
>DDB_G0280575	p	LNDLPKYLLVEILKHL*YLKDIIGLSLV-CKLWAKCIVPVSI-SSFIKI	LRR	
>DDB_G0282285	au	ILNIPFENLLIILQNL-HTKSLLELSQV-NLYFNQIISNSSI-MIWNDI	ApaG	veg
>DDB_G0290983	ab	INNLPQEIIQNILNQLNDIRDLNNCSQV-SSSFYRVCSDQ---IFWKTK	Ankyrin	
>DDB_G0270452	ab	KISNNLIIVKEIVSNL-DVLDLVGIQYV-CKQWNSIATSN---VIWLEA		
>DDB_G0280141	ab	VNGLPEEVLVRIFKIL-NVEDLYKCYSV-CSLWKRLCEDE---AIFRKV		
>*fbxWD*	abtvsm	ILNLPSTIYAQIFSEF-PVKEILKFSLV-CKEFNKAINHK---FLWKIK	WD40+RING	slug
>DDB_G0276383	as	PVFLDSLQVSILFGYL*DSDSLVACSMV-CKLWRKCSLNSN---IWTRF	vWFA	slug
>DDB_G0272020	ab	INDFPEEILIHILKWV-SPFDLRICSLV-SNYWSIISSQN---PIWYEK	Ankyrin	
>DDB_G0282667	a	LNIFSYEVLVHIFSFL-DLTDIKNLKLV-SKDFSNIASECC---LWRMF	Kelch	
>DDB_G0275117	abt	FNLLSTELILYILKYM-GTHDLCILARI-SKRFRECCYDST---LWRTL	LRR	
>DDB_G0276013	ab	FFDLPIEIRMHILSFS-DAVDLSKTCTV-SKYWKSMVDDEQ---LWNNL	Ankyrin	
>DDB_G0272386	ab	ILEFPDEVLLTILSFL-EPNDIKNIYLS-SRYLSSFCFEN---KIWKAI	Kelch	
>DDB_G0292070	ab	IHELPEEIIVKIISYF-SLDTIFNMSLV-SMNFYRLTQDY---DLWKKK	Kelch	
>DDB_G0268070	pvsu	IESLPNIILINIFSII*YHENISDFRFI-CKLWSDLICKN---VSIRIN	LRR	veg
>*dsGG*	ab	TLLLDSEILIRIFSKL-EYSDLFIVKRV-CSRWKPLCEHK---SLFNSL	RabGAP	
>DDB_G0269446	ab	LQDLPTEVFYLILSKV-SSDFIIQEFQYVSKYIYHLCSDE---LFWKLL		
>DDB_G0289577	abt	IRDIPYDLLGKLLALI-LTKEFMSTSLV-CRKWRALARLNGN--HFNLE	LRR	
>DDB_G0270750	b	FNHFPEEILYQIFSRL-SQAGKLIPLQL-DYLIRKSYLADQV-ALVLST	Kelch	
>DDB_G0271408	ps	QDIIDFEILKSIINYL*NINEILNYILV-SKKWYNFISLTIS`KSFEHC	LRR+	slug
>DDB_G0271404	p	QDVIEFEILRSIINYL*NRNEILNYILV-SKKWYNFISLTIS`KLFEYW	LRR+	
>DDB_G0347826	pv	QDIIDFEILKSIINYL*YRNEILNYILV-SKKWYNFISLTIS`KLFEHW		
>DDB_G0276383	ab	VFLDSLQVSILFGYLL-DSDSLVACSMV-CKLWRKCSLNS---NIWTRF	vWFA	
>DDB_G0278671	ab	AIIQSEKLQFKCKNRI-NVQSLIQVSST-NSLLQLISNDN---ELWKKI		
>*gacFF*	av	FSLLPTHLTLYVFSYL-EPKELLILAQV-SSQWQKLAGDN---LLWVRF	Rho-GAP	
>*dlrA*	abtvsm	IGSLADIIISNILGKA-NSQNFTNICSV-CSKWRKISVGRL--VNYTYQ	LRR	
>DDB_G0279513	pvs	INELPIPVLIKLFGFV*SSYWFVNYTLV-CKLWTTQILPT----AWNEM	LRR	veg/slug
>DDB_G0278767	ab	MKSIVLVFNILKHL-EIDDIYCCEIV-CKDWRKVANQN---KLWEIL		
>*fbxE*	avsum	LSTIPEVQLFDILVLL-KPIDLGRL-RV-TSKYFATIVAKDS--LWRRI		veg
>DDB_G0285125	asu	LISLPHQILYQIFESI-SPQDFTNCSKL-NKKWNSVLIKLDK--CWIQY		veg
>DDB_G0289995	apvsm	SQSIYIDSEEIILNNI*DNFFLLDNSVR-FNVFNESHCDDKS`KVWRTQ	LRR/FNIP	
>DDB_G0270322	pvs	INSLPTHLIRKILSYY*LRFRWMDLCLV-CKFWMEMVMPYLP`FYFMDS	LRR	
>*docC*	ab	ISVLNGEVLVCIFQYL-DIKEICCLAQT-CSWWRIVSEQN---VLWLKK		
>DDB_G0294633	ps	YNNI=SILFSKILSNF-KGVKRFGSKVI-SRKYKDIIS-----LEWMIN	Ankyrin	
>*fbxC*	abt	INFLPGTVLLKIFSMV---NENDKPNELPSQSFYQLSLVCK---LWRYY	LRR	
>*mkkA*	abt	NIILPINLIILIFREI-KPNFVNTLSRV-CKHWKQIIDDDE---LWNKY	WD40	
>DDB_G0286129	abt	TLILPEEVMLHILKFLSPIDLCRGVCRV-SLKWRSLAFDIS---LWRDL	WD40	
>DDB_G0285933	b	NDLFSVELMVKIFGMC--DPFSTALILV-CKHWYSVLINNQL-SLWRDL	LRR	
>*jcdI*	bvum	SILEDQILLNVVFNEF-TCSELLKYQCV-SPAFYILLGDDR---LWKDA	JmjC	veg
>*jcdH*	bvu	SVFEDNFIIDSILDLL-SAEELTKT=LV SKTFYIYVQEEE---QWKMR	JmjC	veg
>DDB_G0288687	ps	SNYIQSYLIEYLIEFL*NRNEILGYSLV-CKYWLNSIKKTIK-YNLFDF	LRR+	slug
>DDB_G0271202	pvsu	FEILRSIINYLLISKK*NTNEILNYILV-SKKCIINNKLFE--HWLKIN	LRR+	veg

```
>DDB_G0274183  pv     INNLPHTVITKILYYL*PNLELETICLV-CKLWGCKLAPQVF-TYFTVK    LRR
>DDB_G0278939  pv     NIQLANILIIKILDYR*LYDFVNSIQIV-CKSWNLFIVNKLNLGKYLTK    βsheet      veg
>DDB_G0272482  pu     KSILNEILFHLNISNNYISKRIFTFTDLLDGNFR--SSPFKD--YFKSL    LRR/FNIP
>DDB_G0278029  pv     MALLPIYIQKEIIQIY*YNKILVNLSLV-CWSYHQTLSNYLY`FNFEPW    LRR+        veg
>DDB_G0273009  pv     VVLLPLYIQKYIIKIL*IKRLMLTLALV-SQDWFKTLSNNLT`VDFNYT    LRR+        veg
>DDB_G0273173  p      NSKLPLYIQKYIIKIL*IKELMLSLALV-NHDWFKTLSNNLT`VDFNYN    LRR+        veg
>DDB_G0292302  b      SNGLSDEYWILIFHFL--DFKTLVALSE-TCHLFYRLSNDR--FLWESL    Zinc finger
>DDB_G0271228  pvs    NKILQDVIDYEILKSL-----INYLLIS~INQYKINKFYLNRNEILNYI    LRR+        slug
>pkcA          bs     RLFNSSIIQLKILKYL-DTNTLLNCGLA-SRQLRYNLYNGIS`NFWIKL    ZF+Kinase   slug
>DDB_G0271418  pvs    LRSLINYLL=YKISKF*HRNEILNYILV-SKKWYNFISLTIS’EHWLKI
>DDB_G0293774  pvs    NCWRNEIILNKILFHT*HTTMKMTIDQY-ST-YKYKSYIEN--LIFVNY    LRR/FNIP
>DDB_G0268822  au     LPIDVLDLIFSQLSFI---DRCICAS-V-CSSWRNVLIMQPH-PLLENC    LRR         veg
>DDB_G0275359  p      EINQSAEKVIAFLLDV--PTVAKCYPFV-DSVVKVNANTYKW`RKVGSI    Meth x-ase
>DDB_G0286243  pm     NRVLSNILTIKILKYS*HEQFTETLSLV-CKSWNLFIIPKLNLSRYRFN
>DDB_G0276473  p      MSK-PNVLILGGVGFI---GRNLVQYLV~CNKIRVADKVLPA-TAFLGA    Epimerase
>DDB_G0271434  pvs    KIKIDNKIFQDLIDFEILRSIINYLLTS~SNQYKINKFYFNR`EILNYI    LRR+        slug
>DDB_G0268782  pu                MDKLFQSVF--NNIYISRLIY-SILYKKNEVVRFE-SLLNYT    LRR+        veg
>DDB_G0287541  b      EEESISANYNSYNKKT-ILNVVINFSMT-CKYFYYLFQDD--GFWYRTY    WD40
>DDB_G0292612  ps     NCSFKRILELSLLSKS---IFKKIQYQI~QKEIKFKPEINEF-LNYIYN                slug
>DDB_G0272877  pm     KQILPIDYITVDVKLFEDIPKAVQ=PKV-TVAVENNLKLYAS`DAMQSL
>DDB_G0281481  ps     IIGDISSFALVNKCWFESISNFLTVSID~TIDILRIFGNSIK`TKIEQL
>DDB_G0272060  p      MIKNNNNTYIEINGGG*LDKLSLNLRLV-SHEWNNYISIDNI-TNIFKI    LRR+
```

§ dictybAse.org
+ Domain determined by Swiss Model

‡Key
a   F-box annotated in Uniprot (December 2021)
b   F-box sequence predicted by BLASTp searches in dictyBase.org
p   F-box sequence predicted in Skp1 interactors using Geneious
t   F-box training set
v   Found in mAb 9E10 vegetative co-IP
s   Found in mAb 9E10 slug co-IP
u   Found in pAb UOK77 veg co-IP
m   Found in pAb UOK77 slug co-IP (14)

SR = substrate receptor-like

Loop between helix 1 & 2

```
*DDB_G0280575-pv    (NDEYSSCSGVANTKPQNPFFHNNCYSDQMTNGKDGQDKKQKQCLKNSSSIVNNKPIPASAEPVAATTNNSNNNNNNNQPIIVSQTSSTCV)
*DDB_G0276383-s     (L)
*DDB_G0268070-pv    (SDE)
*DDB_G0271408-pv    (LINKKNNNNNNNTSNQYKINKFYF)
*DDB_G0271404-p     (LISKKKKKNNNYNTSSQYKINKFYL)
*DDB_G0347826-pv    (LTSKKNKNKNNNLNNNTINIYKINKFYF)
*DDB_G0279513-pvs   (NKINS)
*DDB_G0289995-pvs   (VI)
*DDB_G0270322-pv    (YNHCLSMYEYIEKSKGSQFSGYK)
*DDB_G0288687-ps    (LVNKKLKKINKKTLLK)
*DDB_G0274183-pv    (IENKVDLEKSEIRFSGSPEGESASNNGSNNNGQLSMSGGSSGSDGGSPLGSSLLNSLNNSGSNSGNGIIVKKN)
*DDB_G0271202-pvsu  (NKNNNN)
*DDB_G0278029-pv    (CLHYFNVKKILNHKEKRR)
*DDB_G0273009-pv    (CRYID---KNNKSINCVIDDYRGNESQ)
*DDB_G0273173-pv    (CKHIDRNNKLIDSIRYMDIDYRDNEKL)
*DDB_G0278939-pv    (LKSLSHNE)
*DDB_G0293774-pv    (RLFNV)
*DDB_G0286243-m     (IKLSLLDNT)
*DDB_G0271418-pvs   (YL)
*DDB_G0272060-pv    (EIDFI)
```

Loop between helix 2 & 3

```
~DDB_G0271228-pv    (KNNK----TNYNNT)
~DDB_G0276473-ps    (EQKC)
~DDB_G0271434-pv    (KKNKKKNSTNNSNT)
~DDB_G0292612-pv    (SNQ)
~DDB_G0281481-ps    (LDPPKQRT)
```

Loop between helix 3 & C-terminus

```
`DDB_G0271408-pv    (NNIINN)
`DDB_G0271404-pv    (NNIINN)
`DDB_G0347826-pv    (NNIINN)
`DDB_G0289995-pvs   (NIFY)
`DDB_G0270322-pv    (NT)
`DDB_G0278029-pv    (YCIQ---D)
`DDB_G0273009-pv    (VS)
`DDB_G0273173-pv    (VS)
```

```
`pkcA-s             (MEKKTFS)
`DDB_G0275359-pv    (TMQE)
`DDB_G0271418-pvs   (NNIINNKSF)
`DDB_G0271434-pv    (NE)
`DDB_G0268782-pv    (DRE)
`DDB_G0272877-m     (ISVS)
`DDB_G0281481-ps    (NNN)
```

Extra residues in helix

```
=DDB_G0294633-ps    (NNNNNNNNNNNENDDKN)
=jcdH-vu            (MN)
=DDB_G0271418-pvs   (INKTNNNNNNNN)
=DDB_G0272877-m     (SS)
```

Fig. S4. Similarity of *Dictyostelium* F-box-like sequences. *A*, the similarity of F-box-like sequences found in the Skp1 interactome from this study, and an additional group obtained by BLASTp-based searches of the *D. discoideum* genome, are rendered as heat maps as implemented in Geneious (Fig. S3). The map to the lower left of the diagonal compares sequences to FbxwA, and the map to the upper right shows their similarity to the divergent F-box sequence of the FBP JcdI (Fbxo13). *B*, a graphical comparison of relative similarity of the F-box-like sequences to F-box sequences from FbxwA and JcdI. The sequences are ordered from most to least similar from top to bottom, and the lines connect the same sequence from each list.

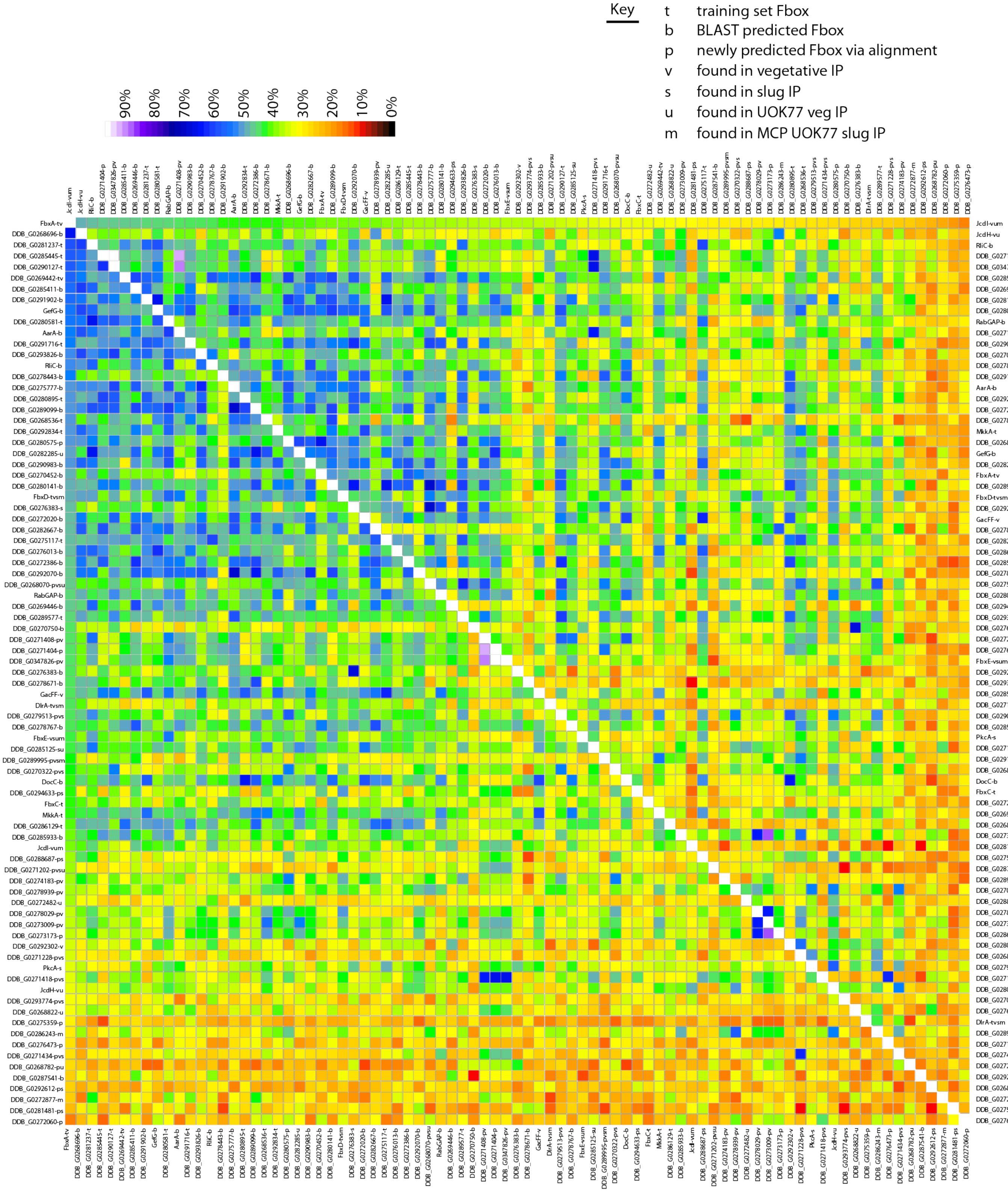

Fig S4B. F-box sequence relatedness, relative to FbxwA (left) or JcdI (right)

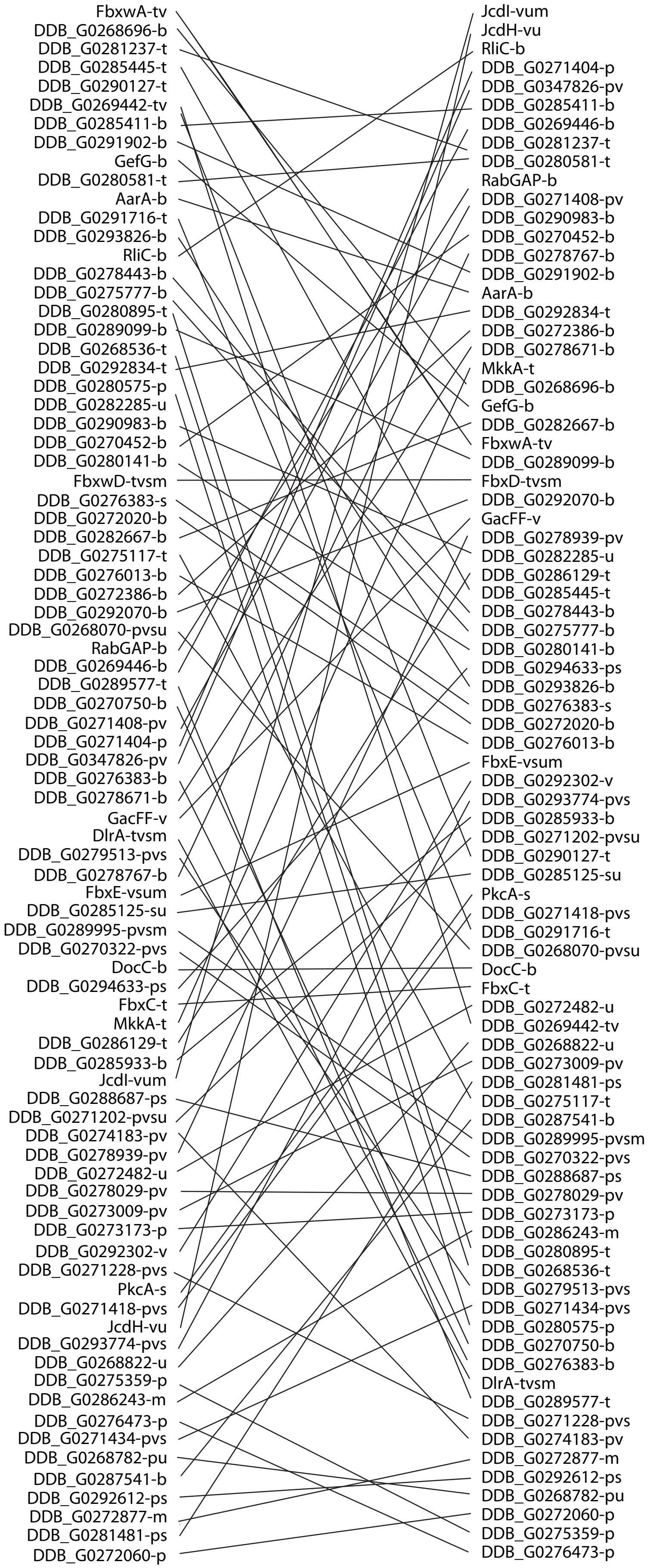

Fig. S5. Tagging the *fbxwD* gene locus. *A*, the 3-exon chromosomal locus of *fbxwD*. *B*, the DNA fragment encoding a C-terminal 3×FLAG tag and a floxed blasticidin S resistance (*bsr*) cassette, flanked by gene specific targeting sequences directing double cross-over homologous recombination. Primers used to amplify gene-specific DNA are shown in red. *C*, The expected edited gene, together with expected PCR amplicons, was verified by PCR. *D*, clone 6 from panel C was transiently transfected with Cre recombinase, resulting in removal of the floxed *bsr* cassette and leaving the FLAG tag coding region intact. Expected PCR product is diagrammed. *E,* confirmation of expected outcomes from panels C and D, based on agarose gel analyses of PCR experiments using the indicated primer pairs, in red.

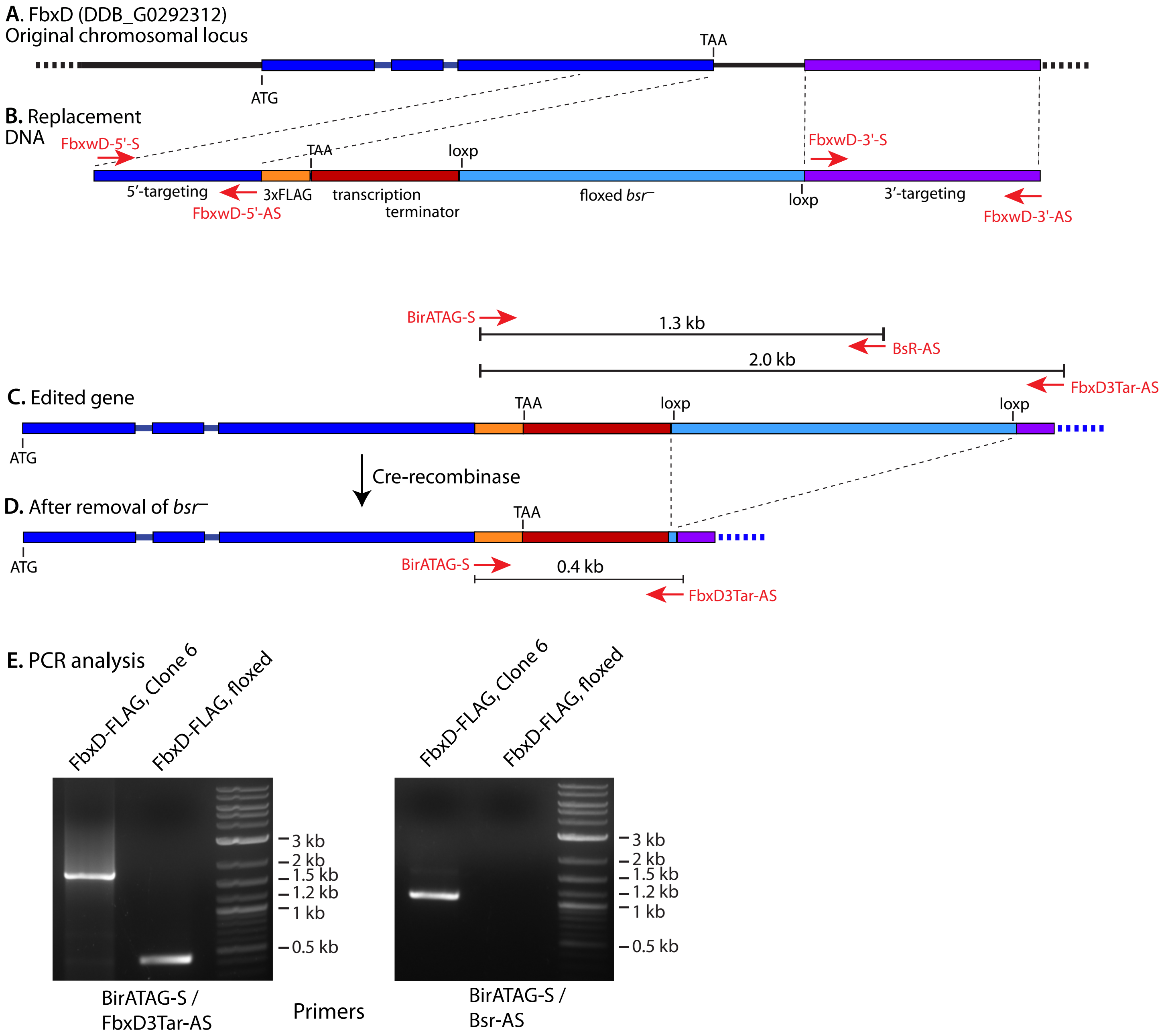

Fig. S6. Expression of FLAG-JcdI in *Dictyostelium*. *A*, the predicted jcdI transcript, derived from 2 exons. *B*, the expected full length coding sequence was generated PCR amplification of the separate exons from genomic DNA and ligation into pCR4-TOPO. *C*, The resulting full length coding sequence was cloned into the pV3D *Dictyostelium* expression vector, which controls expression under the semi-constitutive discoidin 1γ promoter behind an N-terminal His_6-$FLAG_3$-tag. The resulting plasmid was electroporated into *Dictyostelium* under selection with G418, which is expected to result in the chromosomal integration of tandem arrays of FLAG-JcdI and overexpression of the protein. Primers are in Table S1 and Fig. S8. See Methods for additional details.

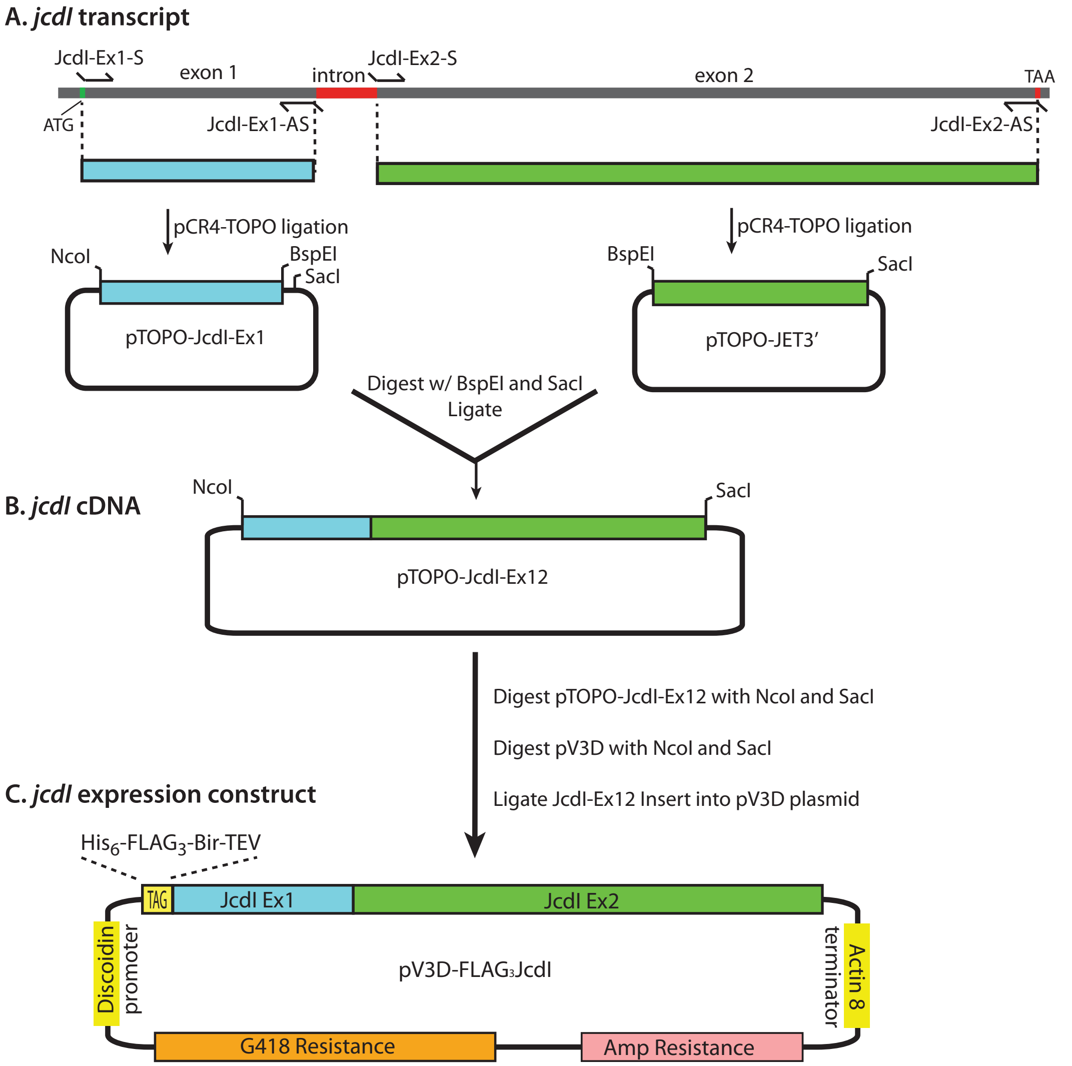

Fig. S7A. Alignment of sequences of JmjC domains of JcdI-related proteins. Sequences are listed in order of correspondence to the evolutionary tree shown in Fig. 8. To facilitate visualization of relatedness, residues are colored and highlighted as in Fig. S3. Positions of near perfect conservation within major groups are bolded, and those that distinguish major JmjD6-like groups are asterisked. Inserts deleted from the alignment are given at the bottom.

```
                                                       *                          *                           **
Polig2      PSPPLYTTP---VYF---QDDWLNWW---WDRKD---ESSDDYRFVYIG---PAGSWTPLHHDVFRSYSWSVNVCGQKEWIFFHPDEESKLK
Ainva3      PETAAYTTP---IFF---QDDWLNWW---WDQPT[e]PTRDDYRFVYLG---PAGSTTPLHHDVLLSYSWSINICGRKEWLLFPPTETWKLW
Cowcz6-J4   PGYNAYELP---VYF---SDDWLNGY---WDECG[d]NRRDDFRFCYIG---PAGSWTPVHFDVMSSFSWSANICGRKKWIFFPPEAREMLT
Badel3      PEYGAYTVP---DIF---ADDWLNGF---WAARK---DTEDDYRFCYMG---GPGTFTPLHADVYRSYSWSANITGIKHWIFFPPDQQPLLR
Spomb1      PADFPYSTP---DIF---ADDWLNAY---VIDCE-----SDDFRFAYLG---SHLTTTGLHTDVYASHSFSVNLCGVKCWLFIDPKDLQTIA
Dmela1-J4   PGYNFYKVP---KYF---ASDWLNEQ---LIQQG-----KDDYRFVYMG---PKNSWTSYHADVFGSFSWSTNIVGLKKWLIMPPGEELKLN
Dreri1-J4   PEHNIYKTP---IYF---SSDWLNEY---WDTIE-----VDDYRFVYMG---PKGSWTPFHADVFRSYSWSANICGRKKWLLYPPGQEDFLR
Mmusc1-J4   DLEDIFTLP---VYF---SSDWLNEF---WDVLN-----VDDYRFVYAG---PRGTWSPFHADIFRSFSWSVNICGKKKWLFFPPGEEEALR
Hsapi1-J4   PVEDVFTLP---VYF---SSDWLNEF---WDALD-----VDDYRFVYAG---PAGSWSPFHADIFRSFSWSVNVCGRKKWLLFPPGQEEALR
Acast3      PGKIAYTLP---LIF---SDDWMNLH---WDSRT---DIDDDYRFVYMG---PKGSWTPLHSDVFGSYSWSANVCGRKRWVFFPPEQ-GPLL
Athal4      PDYTAYQTP---PLF---SDDWLNVY---LDNYQ[a]ISCSDYRFVYMG---GKGSWTPLHADVFRSYSWSANVCGKKRWIFLPPPQ-SHLV
Acast1      KLLEDYEVP---PYF---PEDFFSLM---G-SER------PFYRWVVIG---GPRSGSPFHLDPFKTSAWNALLVGRKRWVIYPPNQVPPSG
Ngrub1      RLLDEYEIP---QLF---KEDFFEQC[f]SLDER------PLFRWLVVG---PARSGTQFHMDPYLTSAWNALLSGRKRWLFYPMSHVSEDL
Acast2      ALLEEYQVP---EYF---REDLFAAM---G-STR------PDYRWVIVG---PPRSGSAFHQDPNRTAAWNALISGRKRWIMFPPHVLPPGV
Acast4      AMLEDYSIP---KYF---TEDYFAYL---EEPER------PSFRWILVG---PTRSGATFHKDPNHTSAWNGLLWGLKKWLLYPPNVVPPGT
Dfasc2-H    EMLDMYSQE---KYF---DEDFFNVL---G-DKR------PSYRWLLAG---PKRSGATFHKDPNHTSAWNGVITGRKKWVMYPPHVVPPGV
Asubg2-H    SLLDDYSTD---RYF---KEDLFAVL---G-KSR------PSYRWLLAG---PARSGATFHKDPNHTSAWNAVVTGRKKWVMYPPHITPPGV
Dpurp2-H    ELIKEYSTE---HIF---KEDIFSVL---G-DKR------PSFRWLLAG---PKRSGASFHKDPNHTSAWNAVITGRKKWVMYPPHVVPPGV
Ddisc2-H    SLLEDYSAD---AYF---KEDLFNVL---G-DKR------PSFRWLLAG---PPRSGASFHKDPNHTSAWNAVITGRKKWIMYPPHVVPPGV
Bdend2      FLSADYVVP---KYF---SQDLFQVL---G-DNR------PDYRWLIIG---PARSGSTFHIDPNSTSAWNAVITGAKKWILYPPECIPPGV
Tcruz1      AIESLYSVP---EHF[f]CDDFFKVL---G-DAR------PKHRWIIAG---PRRSGSNFHVDPNYTNAWNANLAGRKRWILFPPGCTPAGV
Athal3      VLDSEYDVP---VYF---REDLFGVL---G-NER------PDYRWIIIG---PAGSGSSFHIDPNSTSAWNAVITGSKKWVLFPPDVVPPGV
Ainva1      QLAADYTVP---AYF---HDDLFPLL---GETER------PDYRWLIFG---PEGSGSSFHIDPNSTCAWNAVLRGRKKWIMFPPDVVPPGV
Psoja3      QLAADYTVP---EYF---QEDYFSLL---GEDKR------PDYRWLIIG---PKKSGSSFHIDPNATNAWNGVIRGSKKWIMFPPGQVPPGI
Polig4      ALAADYTVP---EYF---QEDFFSYL---G-GKR------PDYRWLIIG---PKKSGSSFHIDPNSTNAWNGVIRGSKKWIMFPPDIVPPGV
Tgond2      SLAEDYEVP---PYF[g]SRDLFACL---G-ERR------PNFRWLLVG---NCRSGSKWHVDPNQTSAWNAVVRGAKRWILLPPTVCPPGV
Csube2      ALASDYHVP---EQF---GEDLFGLL---GETGR------PDYRWLILG---PARSGSSFHVDPNATSAWNALVWGAKKWVMFPPGVVPPGV
Crein2      KLAADYEVP---EYF---AEDLFGVL---GEDAR------PHHRWLIMG---PARSGSSFHKDPNATSAWNAVIKGSKKWILFPPHVTPPGV
Dpurp1-I    SLLEQYKVP---KYF---PEDLFQYS---GPEER------PHFRWLVIG---PERSGASWHIDPAGTSAWNSLISGKKRWLMYPPAFTPYTV
Ddisc1-I    SLLDDYNIP---KFF---PEDLFKYN---GEEHR------PHFRWIVIG---PERSGASWHIDPAGTSAWNSLISGRKRWLMYPPNFTPYTV
Dfasc1-I    DMLSEYSVP---PYF---PEDLFACS---GEKER------PHFRWIVIG---PPRSGAPWHIDPAGTSAWNSLISGRKRWLMYPPQITPIGV
Asubg1-I    NMLKDYAVH[j]KFF---PEDLFQYQ---G-EKR------PHYRWIVIG---PPRSGAPWHIDPAGTSAWNSLVSGRKRWLMYPPSINPIGV
Ppall1-I    AMLNEYKVP[k]KFF---PEDLFQFQ---N-DKR------PHYRWIVIG---PPRSGAPWHIDPAGTSAWNSLVSGCKRWLMYPPSSTPIGV
Cowcz3      EMLHEYKVP---KMF[h]DHDYLAFF---G-NER------PDFRWIVIG---PPRSGASWHVDPTHTSAWNSLIAGRKRWALYPPGQIPPGV
Spunc2      DMLRDYEVP---SIF---AEDFLSVL---G-EER------PPFRWLVIG---PARSGASWHIDPLGTSAWNTLLSGRKRWALYPPDKLPPGV
Gmax1       SLLKDYCVP---HLF---QEDFFDIL---DTEKR------PSYRWLIIG---PERSGASWHVDPALTSAWNTLLCGRKRWALYPPGKVPLGV
Athal2      ELLKDYSVP---HLF---QEDWFEIL---DKESR------PPYRWLIVG---PERSGASWHVDPALTSAWNTLLCGRKRWALYPPGKVPLGV
Ppate2      EMLEEYSVP---PLF---SEDLLAVL---DKPVR------PPFRWLVAG---PARSGASWHVDPALTSAWNSLLSGRKRWALYPPGRVPPAV
Ppate1      DMLEEYSIP---PVF---SEDLLAVL---DKSVR------PPFRWLVAG---PARSGASWHVDPALTSAWNTLLSGRKRWALYPPGRVPPAV
Badel1      GIVDDYKVL---EYF---QRDFLKLL---PTESQ------PPYRWLIIG---PQRSGASWHTDPNGTSAWNTLISGRKRWALYPPACTPPGV
Ainva4      SLLSDYVIP---NVF---NEDLLAVL---PLEMR------PDFRWLVVG---PARSGASWHVDPAKTSAWNALLVGRKRWALYPPGRCPPGV
Psoja2      AMLEDYDVE[L]KVF---KEDFLSVI[b]PGSIR------PDFRWIVIG---PQRTGAPWHQDPARTSAWNSLVKGRKRWAIYPPDSPPPGV
Polig3      SLLGDYNVA[L]KFF---REDLLKTP---AVSTA[c]CIDPDFRWIVIG---PERSGAPWHTDPARTSAWNALLQGHKRWAIYPPGCPPPGI
Gthet1      SMAQDFSIP---TYF---QEDLFKYL---GEDDR------PPYRWVLVG---PKRSGSSIHIDPCGTSAWNSLLAGRKRWVLFPPGTPRSVI
Aanop1      AVVGAFRAP---TFC[i]RDDLFELV---GERRR------PPHRWLLVG---PRRSGTCAHVDPLGTSAWNTLLTGRKRWVLFEPGTSRHVA
Tther1      KMIEKYEVP---KYF---REDLFQYI---GERKR------PPYRWFLIG---PERSGTTVHIDPLQTSAWNTSLQGYKRWVLFDPNVPKSVV
Ptetr3      DIIGRFKVH---KYF---SEDLLALV---GEKHR------PPYRWFLVG[m]PQRSGTTVHIDPLMTSAWNTSLQGHKLWVLFPPDIPKCVV
Ptetr2      NMIGRYKQH---KYF---QDDFLSLV---GEKHR------PPYRWVLVG---PKRSGTTVHIDPLMTSAWNTSLQGHKLWVLFPPDIPKSIV
Ptetr1      DMVGRYKYH---KYF---QEDFFSVV---GEKHR------PPYRWFLVG---PKRSGTTVHIDPLMTSAWNTSLQGHKLWVLFPPDIPKSIV
Tgond1      GLLDDWTVP---EVF---PMDLHAIV---GEERR------PPHRWFCIG---PKRSGTTVHIDPLGTAAWNAVTHGVKRWALFPPAVPRHVV
Ptric1      RVLADYRVP---SYF---SDDLFQLV---SEARR------PPYRWFLVG---PERSGSTVHVDPLATSAWNTLMFGKKRWVLFPPQVPKQVV
Ptetr4      KMLNQYKIP---YLF---PQDYLAYL----KQRR------PQYRWILCG---PKQSGSMIHIDPYETSAWNCVVLGKKRWVMFPPSIDKNII
Psoja1      PLLDDYKVP---KYF---PEDLFSLV---GEDRR------PPYRWFLVG---PKRSGTTLHLDPLGTSAWNTLLVGRKRWVLFPPHLPKNLV
Polig1      PLLADYAVP---RYF---PEDLFSLV---GEDRR------PPYRWFLVG---PKRSGTCVHLDPLGTSAWNTLLVGRKRWVLFPPHVSKEIV
Ainva2      PLLSDYNVP---RYF---PDDLFSLV---GEDPR------PPYRWFLVG---PKRSGTCVHVDPLGTSAWNTLIVGRKRWVVFPPSVDKKTV
Otaur1      SLLEDFSIP---EYF---EEDLFKHV---GRKRR------PPYRWVVFG---PPRSGSSVHVDPLATSAWNALISGRKRWALYPPRSVTRAQ
Csube1      GLRREYEVP---HYF---QEDLMRLA---GDKRR------PPYRWLVMG---PGRSGSGLHIDPLATSAWNALVQGHKRWALFPPGTPRHVV
Crein1      AMRRDYEVP---AYF---REDLFGLV---GEGRR------PPYRWVVMG---PARSGSGLHIDPLGTSAWNTLLAGHKRWALFPPGTPRAHV
Badel2      NMLDDYVVP---HFF---ADDLFKHT---GERRR------PPYRWFVMG---SARSGTGIHIDPLGTSAWNTVIEGHKRWCLFPPWVSKETV
Pblak1      LLLGDYQVP---SYF---SDDLFRLA---RERRR------PPYRWLVMG---GARSGTGIHIDPLGTSAWNALLKGHKRWCLFPPGTPKKII
Ptric2      SLLDDYTVP---SCF---DSDLFASA-IPNEDDR------PPFRWLLIG---PARSGTGLHIDPVGTHAWVTLIEGCKRWILFPAGTDPEAI
Bdend1      HLIDDFELP---KYF---TDDLFRLV---GKRRR------PPYRWIVIG---PARSGTGIHIDPLGTSAWNALLQGHKRWVLFPPGAPKDII
Spunc1      KLLEDYEVP---KYF---RDDLFRLC---GERRR------PPYRWMVVG---PARSGTGIHVDPLGTSAWNAIVYGHKRWVLFPPGTPRDII
Cowcz4      QLLDDYTVP---KFF---RDDLFKHA---G-SQR------PPFRWFVMG---PKRSGTCIHIDPLSTSAWNTLIQGHKRWCLFPPHAPRELV
Celeg1      KLSEDYSVP---KFF---EDDLFHYA---DDKKR------PPHRWFVMG---PARSGTAIHIDPLGTSAWNSLLQGHKRWVLIPPIAPRDLV
Dreri2-J6   KLLEDYQVP---LFF---RDDLFQFA---GEKRR------PPYRWFVMG---PARSGTGIHIDPLGTSAWNALVQGHKRWCLFPTHTPRELI
Mmusc2-J6   KLLEDYKVP---KFF---TDDLFQYA---GEKRR------PPYRWFVMG---PPRSGTGIHIDPLGTSAWNALVQGHKRWCLFPTNTPRELI
Hsapi2-J6   KLLEDYKVP---KFF---TDDLFQYA---GEKRR------PPYRWFVMG---PPRSGTGIHIDPLGTSAWNALVQGHKRWCLFPTSTPRELI
Dmela2-J6   KLLDDYVVP---KYF---RDDLFQYC---GENRR------PPYRWFVMG---PARSGTGIHIDPLGTSAWNTLIRGHKRWCLFPTQTPKELL
Tadha1      RLLNNYEIP---EFF---QDDLFNYS---EEKRR------PPHRWFVLG---PARSGTGIHIDPLGTSAWNSLISGHKRWALFPTTTPKELL
Hvulg1      KLLDDYHPP---SFF---QDDLFKYA---GEKRR------PPYRWIVIG---PARSGTGIHIDPLGTSAWNALISGHKRWMMFPTETPKHLL
```

```
            ****          *      **                *    *    *                      *
Polig2      -DKFGRTVLPDI--TSK--DYDKDL--YPRAHE---------ATPLYVVQDSGEAIFVPSGWYHQVRNVRD---TISINHNWFNGFNI
Ainva3      -DRSGRCTATNA--TE----FDATQ--FPHLAT---------AHHVRVVQGVGEALFVPSGWYHQVQNLDR[u]TLSVNHNWFNAYSL
Cowcz6      -DAAGELLS-DV--RS----VDETR--FPNFRN---------APRIELFQEEGQLVFVPSQWYHQVINLTD---VISINHNWANGCNI
Badel3      SVKTGQPPY-LI--SEA--LADPAE--YPNAAK---------AVRIDVWQFPGETIFVPSGWWHQVINESH---TISINHNWANACNL
Spomb1      -SLYDDQQLPSW--ITK---DDLFR--GPLVNH--------RHLIKILFQYPGQTVFVPSGWYHQVLNIGT---TLSINHNWCNASCI
Dmela1-J4   -DRLGNLPF-SI--DEK--MLDEHN--VR----------------YYTINQRANEAVFVPSGWFHQVWNLTD---TISVNHNWFNGCNI
Dreri1-J4   -DCHGNLAY-DV--TAP-ILQDKGL--YAQFEEA--------CQPLEIIQEAGEIIFVPSGWHHQVYNLED---TISINHNWLNGCNL
Mmusc1-J4   -DCHGNLPY-DV--TST-ELLDTHL--YPKIQHH--------SLPIEVIQEPGEMVFVPSGWHHQVYNLDD---TISINHNWLNGCNL
Hsapi1-J4   -DRHGNLPY-DV--TSP-ALCDTHL--HPRNQLA--------GPPLEITQEAGEMVFVPSGWHHQVHNLDD---TISINHNWVNGFNL
Acast3      KDKFGNLMY-DI--EED--QKVSAS--ASAARG---------ARPISCVQEAGETIFVPSGWFHQVWNEED---TISINHNWANACNV
Athal4      YDRYMKNCVYDI--FE---EVNETK--FPGFKK---------TTWLECIQEPGEIIFVPSGWHHQVYNLED---TISINHNWLNAYNL
Acast1      VDVDEDEDTGEIDYTGEDPIV-WFLEHYPLIKNR--------QHPIECILEEGEIIYVPTNWWHMVFNLTE---TVAVTQNFCDSHNF
Ngrub1      [v]NAQPVPSYV--PCSEPIQ-WLTNEYYEALNQ--------GRRPWECVQYPGDLIFVPSTWWHMVLNLDD---TFAVTQNFCDSSNV
Acast2      ---EKDEHGNTL--PIADSVIGWYINYYEELQEE--------VDFVECIQYPGELIF---GWWHMVLNLDE---TIAVTQNFVSSQNL
Acast4      ---FPSEDEWEV--TTPISIVEWFYNFYQETDKP--------QRPIECLLRPGDMIFIPNGWWHTVLNLEE---SVAVTQNYVGRHNV
Dfasc2-H    ---HPSDDGLEV--TTPHSIVEWFINYYDTDVKS--------YKPLECILNPGELIFVPCGWWHCVLNMEE---SIAITHNFINSNNI
Asubg2-H    ---HPSDDGLEV--TTPHSIVEWFINYYERPDNN--------GYPLECVLEAGEMIYVPCGWWHCVLNLEE---SIAITHNFIDSHNI
Dpurp2-H    ---HPSDDGLEV--TAPSSIIEWFLNYYEKPNKK--------VKPLEGILNAGELIYVPCGWWHCVLNLEE---SIAVTHNFIDSQNI
Ddisc2-H    ---YPSDDGLEI--TAPSSIIEWFINFYEKPDND--------VKPLEGILHAGEMIFVPSGWYHSVLNLEE---SIAITHNFIDSNNL
Bdend2      ---FPSKDGSNV--TSPVSLAEWFMNYYQEIHSSAGA[n]CTPKPIECICRAGEMIFVPNGWWHCVMNLTD---SIAITQNFVS----
Tcruz1      ---FPAEDMSEV--TTPVSLSEWLLNYYDASVERWR------GVGYECICEPGDIMFIPCGWWHFVINLED---SVAITQNYVSESNL
Athal3      ---HPSPDGAEV--ACPVSIIEWFMNFYDDTKDW--------KKPIECICKAGEVMFVPNGWWHLVINLEE---SIAITQNYASRSNL
Ainva1      ---HPSVDGGEV--STPVSLMEWFVTFYPQIKSL--------VRHLEGVCEAGEMIFVPRGWWHIVLNLDE---SLAITQNYVSPSNV
Psoja3      ---HPSEDGSEV--SSPVSLMEWFVTFYKQVQKLPS------HLKPLEGICREGETMFVPHGWWHTVLNLDE---CIAMTQNYVSSGNV
Polig4      ---HPSEDGADV--SSPVSLMEWFVTFYPQVKKLPA-----HQRPLEGICREGEILFVPNGWWHIVLNIEE---SIAITQNFVCRGNV
Tgond2      ---FPSHDGGEV--TQPTALVEWLMNYYFDALHA--------IAPLEGSVREGELIFVPQGWWHCVLNEED[u]TIAVTQNFVSPVIL
Csube2      ---HASADGADV--ATPVSLVEWMLNFYDASQRM-------RVPPVEGVVRAGEVLFVPRGWWHFAINLEE---SIAVTQNYVSSAGL
Crein2      ---HPSPDGADV--ATPVSLAEWFVNFYPECAEM-------KVKPVEFVARPGELLFVPHGWWHCALNLTD---SCAITQNFVSAVGL
Dpurp1-I    ---DLEDFEEKIY-GSPPSLL-WLLEVYPYLPPD--------YRPIECIQEPGETIFVPGGWWHMVLNLEE---SIAVTQNFCDSQNF
Ddisc1-I    ---ESDEVVDKIY-GSPPSLL-WLLEVYPYLPPD--------YRPIECIQEPGETIFVPGGWWHMVLNMEQ---SIAVTQNFCNSQNF
Dfasc1-I    ---SMEDIDEKFY-GSPPSLL-WLLEVYPYLPPD--------QKPIECIQNPGETIFVPGGWWHMVLNLEE---SIAVTQNFCDSQNF
Asubg1-I    ---ELEDIVEKFY-GSPGSLL-WLLEVYPYLPPD--------QRPIECIQNPGETIFVPGGWWHMVLNLEE---SIAVTQNFCDSQNF
Ppall1-I    ---SMDDVDEKFY-GGPASLL-WLLEVYPYLPPD--------QRPIEVIQYPGETIFVPGGWWHMVLNLEE---SIAVTQNFCDSQNF
Cowcz3      -EAIFDDHGNIVDFNAPPSLL-WYLEVYPFLDPE--------EKPIECVQHPGETISVPAGWWHMVLNLDY---TCAVTQNYLNVNNL
Spunc2      ---HPSDL------SSPSTLY-WYLEVYPFLPPE--------LKPIEIVQEPGETIVVPAGWWHAVLNLDQ[t]NIAITQNWCGRWNL
Gmax1       -TVHVNEEDGDVNVETPSSLQ-WWLDFYPLLADE--------DKPIECTQLPGETIYVPSGWWHCVLNLET---TIAVTQNFVNSNNF
Athal2      -TVHVNEDDGDVSIDTPSSLQ-WWLDYYPLLADE--------DKPIECTLLPGETIYVPSGWWHCILNLEP---TVAVTQNFVNKENF
Ppate2      -VVHVDKSDGSVNFDGPTSLQ-WWLEVYPTLREE--------DKPFECTQLPGETISVPSGWWHCVLNIDD---SIAVTQNYVNSTNL
Ppate1      -VVHVDLDDGSVNFDGPTSLQ-WWLEVYPTLRDE--------DKPLECTQLPGETISVPSGWWHCVLNIDD---SVAVTQNYVNSSNL
Badel1      ---YIDESFNYEEDTTYTSLR-WYLEVYPHLAAS--------DRPVEVVQEAGETIFVPSGWWHMVLNLED---TVAVTQNFADRGNI
Ainva4      --TLLDEDTA---SPATSSLD-WFLHVYPTLSDA--------DRPFEVVQEPGDVISVPSGWWHVVLNLEF---TIAVTQNVVDSHNV
Psoja2      --NVGKNGEYRDSGLDMPSLM-WYLHVYPTLTPD--------QKPLEIIQEEGETIYVPNGWWHLVLNLDL---TIAVTQNFVDSHNA
Polig3      --KVGKNAAGREHALNMTSLS-WYLHVYPTLAPH--------ERPYEIVQGPGDVIYVPSGWWHLVLNLDE---TIAVTQNFVDSHNL
Gthet1      KPESWL------AQKRSEALD-WFLYHLDGMKQQLPA----HQQPVEVIMEAGETIFVPGGWWHTVLNLED---TIAVTQNFVSSNNF
Aanop1      KGSRLYD----PRVEDDEAIN-YFVDILPRIRAAYPE-------ARRIECIQEPGETIFVPGGWWHAVINLED---TIGVTQNFASRGNF
Tther1      KAKKFI-----PTGEDDDAIQ-YFCKMLPKLVQEEGR---ENLGIIEFIQGPGETVFVPGGWWHAVINVTD---TVAVTQNYMNSVNI
Ptetr3      KAKGLAAKRLIDPVDLDESID-YFMHALPKLIEQEGA---DNLKIIMGIQGPGDTIFVPGGWWHAVLNLDN---TIAITQNFMSSNNF
Ptetr2      KAKGLAAKKEIDFEVLDESID-YFLYALPKLIEKEGA---DNLKIVMCVQGPGDTIFVPGGWWHAVLNLDN---SVALTQNFMSINNF
Ptetr1      KAKGLAAKKEIDPEVLDESID-YFLYALPKLIEKEGA---DNLKIVMCVQGPGDTIFVPGGWWHAVLNLDN---SVALTQNFMSINNF
Tgond1      KAKHLLK----RG-EDDEAIM-WFDFLLPRIREK--------VPIYECLQKPGEVIYVPGGWWHAVLNLTD---CVACTQNFVSFSFL
Ptric1      KGRGLV-----RRDEDDEAIH-YFMFILPRIKRKAAS[o]KDFACYEFTQNAGETCFIPHGWWHAVLNLTH---TVGVTQNFCSERNF
Ptetr4      KGIKDI------LENINNPID-YFSIIVPLVKKHCDQ---QNIKYYDFIQSEHETVYVPNGWWHAVLNVED---SIAVTQNYVSDQNL
Psoja1      NGKKHV-----RGNEDDEAVN-YFMDLLPRLKRA--------LQCIEFMQYPGETVYIPGGWWHAVFNVDD---TVAVTQNYCSSQNF
Polig1      KGKSHIK----KG-EDDEAVN-YFMDILPRIKKA--------LQCIELMQYPGDTIFVPGGWWHAVFNVED---TVAVTQNYCSSQNF
Ainva2      KAKWHVL----PG-EDDEAGN-YFCDMLPRMVAE--------LEYMEFMQYPGDTVFIPGGWWHAVYNVDD---TIAVTQNYCSHANF
Otaur1      IKP-------RGIGLDGESVT-WFNKMYPRTRTEEWR[s]GLPPPIDVIQYPGEIMFVPDGWWHAVLNLDH---TMAVTQNFATTARF
Csube1      LP--------REKGLEREAVS-WFTVMYPRTQAPDWP----TARPINIIQGPGETVYVPGGWWHTVLNLDL---TIAVTHNYCSSATF
Crein1      LP--------KEQGVEREAVS-WFGKVWPRAQAPDWP----TARCVDLIQAPGETVFVPGGWWHAVLNLDD---TVAVTQNYVSTANF
Badel2      DP---------PGKPDHEAAT-WFSVVFPKLKEK--------LGMIEILQKPGETVFVPGGWWHHVVMLDL---SIAITQNFCSPINL
Pblak1      DPP--------MKPYDHEGVS-WFSTVFPKFKVRDGP[p]ERLGMVEVLQRPGETIFVPGGWPHVVMNLDT---TIAVTQNFCSPTNL
Ptric2      HM---------RDPQIPSAI--WFRDFYDQAMRD--------HADAVEVLQRPGETVFVPAGWPHLVLNLEL---SVAITHNFATEYPS
Bdend1      EP---------KSLQDHEAVT-WFTHVYPKLSDQHPN[q]QVFGMIDILQGPGETVFVPGGWSHVVMNIDF---TVAITQNFCSRTNI
Spunc1      EP---------AHVPDHEAAS-WFAHVYPSMEARTSD[r]ERLGMREIIQKPGETVFVPGGWHHVVINCDF---TIAVTQNFCSRTNL
Cowcz4      KPAI-------SG-MDSEAAC-WFSSVYPRTQSP--------LRPLELLQRPGETVFVPGGWHHVVLNLDT---TIAITQNFASCTNF
Celeg1      KPMAHE-----KGKHPDEGIT-WFQTVYKRVRSPSWP---KEYAPIECRQGPGETMFVPSGWWHVVINEEY---TIAVTHNYCSVENL
Dreri2-J6   KVTRDE-----GGNQQDEAIT-WFNVIYPRTQQSTWP---DEFRPLEILQRPGETVFVPGGWWHVVLNLDT---TIAVTQNFASTTNF
Mmusc2-J6   KVTREE-----GGNQQDEAIT-WFNVIYPRTQLPTWP---PEFKPLEILQKPGETVFVPGGWWHVVLNLDT---TIAITQNFASSTNF
Hsapi2-J6   KVTRDE-----GGNQQDEAIT-WFNVIYPRTQLP--------FKPLEILQKPGETVFVPGGWWHVVLNLDT---TIAITQNFASSTNF
Dmela2-J6   KVTSAM-----GGKQRDEAIT-WFSTIYPRTQLP--------YRPIEVLQGAGETVFVPGGWWHVVLNMDD---TIAVTQNFSSQTNF
Tadha1      KVSGKL-----GGNQRDEAVT-WFSIIYPKTQLSTWP---LQFKPLEILQKPGETVFVPGGWWHVVVNVDM---TIAVTQNFCSPTNF
Hvulg1      EVSKQD-----GQHQSGEGIQ-WFVKVYPKVKSPTWP---KEYAPLEIIQHPGETVFVPGGWWHVVLNLDQ---TVAVTQNFSSPTNF
```

* denotes positions that characteristically differ between JmjD6-like clades
[brackets] refer to residues removed from the alignment used for the tree generation

[a] MHEDRDSFQKYDQ
[b] ETPEEEEGNKVPTKSVKLAAGGKKIRKDKKKKRA
[c] PSTDGKKKKPG

[d] SLDQ
[e] T
[f] L
[g] SD
[h] V
[i] G
[j] D
[k] S
[l] DL
[m] Y
[n] SNQNTK
[o] LKHHEDY
[p] DDNRTLGE
[q] SPTGKTYA
[r] GKTLG
[s] RQ
[t] C
[u] D
[v] EEAIEEMKSAEQEMIQEKLQFEKQIKTKLLHEGKINKIYEDEFEIKD

Fig. 7B. Identifier list for JmjC-proteins in Fig. 8 and Fig. S7A.
Names are color coded according to the phylogram in Fig. 8 and the sequence names in Fig. 7A. Domain predictions are based on amino acid sequence homologies as illustrated in Fig. 8. +wgs refers to additional sequence information derived from the NCBI wgs (whole genome shotgun) database.

Name	Unique ID	Additional Domains	Species	Classification
Polig2	TMW68560.1	3,6	*P. oligandrum*	stramenopile/oomycete
Ainva3	XP_008878937.1	3,6	*A. invadans*	stramenopile/oomycete
Cowcz6-J4	XP_004349321.2	3,6	*C. owczarzaki*	pre-metazoan
Badel3	OZJ02352.1	3,6	*B. adelaidae*	fungi
Spomb1	NP_593806.1	3,6	*Schizosaccharomyces pombe*	yeast
Dmela1-J4	NP_609870.1	3,6	*D. melanogaster*	insect
Dreri1-J4	NP_001070096.1	3,6	*D. rerio*	fish
Mmusc1-J4	NP_848774.1	3,6	*Mus musculus*	mammal
Hsapi1-J4	NP_075383.2	3,6	*Homo sapiens*	mammal
Acast3	XP_004352486.1	3,6	*A. castellanii*	amoebozoan
Athal4	OAO92488.1	3,6	*A. thaliana*	higher plant
Acast1	XP_004341476.1	Fbox,1,3,4	*Acanthamoeba castellanii*	amoebozoan
Ngrub1	XP_002678667.1	Fbox,1,3,4	*Naegleria gruberi*	amoeba-flagellate
Acast2	XP_004347756.1	Fbox,1,3,4	*A. castellanii*	amoebozoan
Acast4	XP_004353539.1	H1,Fbox,1,3,4,H2	*A. castellanii*	amoebozoa
Dfasc2-H	XP_004354443.1	H1,Fbox,1,3,4,H2	*D. fasciculatum*	amoebozoa/cellular slime mold
Asubg2-H	XP_012752744.1	H1,Fbox,1,3,4,H2	*A. subglobosum*	amoebozoa/cellular slime mold
Dpurp2-H	XP_003290928.1	H1,Fbox,1,3,4,H2	*D. purpureum*	amoebozoa/cellular slime mold
Ddisc2-H	XP_641202.1	H1,Fbox,1,3,4,H2	*D. discoideum*	amoebozoa/cellular slime mold
Bdend2	XP_006674897.1+wgs	H1,Fbox,1,3,4,H2	*Batrachochytrium dendrobatidis*	fungi
Tcruz1	XP_821123.1	Fbox,1,3,4	*Trypanosoma cruzi*	kinetoplast
Athal3	OAO90355.1	H1,Fbox,1,3,4,H2	*A. thaliana*	higher plant
Ainva1	RHY25091.1	Fbox,1,3,4	*Aphanomyces invadans*	Stramenopile/oomycete
Psoja3	XP_009537045.1	H1,Fbox,1,3,4,H2	*P. sojae*	Stramenopile/oomycete
Polig4	TMW62047.1	H1,Fbox,1,3,4,H2	*P. oligandrum*	Stramenopile/oomycete
Tgond2	XP_018637735.1	H1,Fbox,1,3,4	*Toxoplasma gondii*	alveolate/apicomplexan
Csube2	XP_005649358.1+wgs	Fbox,1,3,4	*Coccomyxa subellipsoidea*	alga
Crein2	XP_042918101.1	H1,Fbox,1,3,4,H2	*Chlamydomonas reinhardtii*	alga (green)
Dpurp1-I	XP_003290864.1	Fbox,1,3,4,I1-5	*D. purpureum*	amoebozoa/cellular slime mold
Ddisc1-I	XP_646466.1	Fbox,1,3,4,I1-5	*Dictyostelium discoideum*	amoebozoa/cellular slime mold
Dfasc1-I	XP_004352022.1	Fbox,1,3,4,I1-5	*D. fasciculatum*	amoebozoa/cellular slime mold
Asubg1-I	XP_012750272.1	Fbox,1,3,4,I1-5	*Acytostelium subglobosum*	amoebozoa/cellular slime mold
Ppall1-I	XP_020436685.1	Fbox,1,3,4,I1-5	*Polysphondolium pallidum*	amoebozoa/cellular slime mold
Cowcz3	KJE92120.1	Fbox,1,3,4,I1-5	*Capsaspora owczarzaki*	pre-metazoan
Spunc2	XP_016606711.1	Fbox,1,3,4,I1-5	*Spizellomyces punctatus*	fungi
Gmax1	XP_003526572.1	Fbox,1,3,4,I1-5	*Glycine max*	higher plant
Athal2	OAP18381.1	Fbox,1,3,4,I1-5	*Arabidopsis thaliana*	higher plant
Ppate2	PNR51222.1	Fbox,1,3,4,I1-5	*P. patens*	moss
Ppate1	PNR45388.1	Fbox,1,3,4,I1-5	*Physcomitrella patens*	moss
Badel1	OZJ05318.1	Fbox,1,3,4,I1-5	*Bifiguratus adelaidae*	fungi
Ainva4	XP_008866134.1	Fbox,1,3,4,I1-5	*Aphanomyces invadans*	Stramenopile/oomycete
Psoja2	XP_009522873.1	Fbox,1,3,4,I1-5	*Phytophthora sojae*	Stramenopile/oomycete
Polig3	TMW64162.1	Fbox,1,3,4,I1-5	*Pythium oligandrum*	Stramenopile/oomycete
Gthet1	XP_005825990.1	2,3,5(half)	*Guillardia theta*	cryptomonad/flagellate alga
Aanop1	XP_009035976.1+wgs	2,3,5	*Aureococcus anophagefferens*	alga
Tther1	XP_012656085.1	2,3,5	*Tetrahymena thermophila*	ciliophora
Ptetr3	XP_001450283.1	2,3,5	*P. tetraurelia*	ciliophora
Ptetr2	XP_001441376.1	2,3,5	*P. tetraurelia*	ciliophora
Ptetr1	XP_001431059.1	2,3,5	*P. tetraurelia*	ciliophora
Tgond1	XP_002365269.1	2,3,5	*T. gondii*	alveolate/apicomplexan
Ptric1	XP_002181370.1+wgs	2,3,5	*Phaeodactylum tricornutum*	stramenopile/diatom
Ptetr4	XP_001439800.1	2,3,5	*Paramecium tetraurelia*	ciliophora
Psoja1	XP_009531545.1	2,3,5	*P. sojae*	Stramenopile/oomycete
Polig1	TMW69603.1	2,3,5	*Pythium oligandrum*	Stramenopile/oomycete
Ainva2	RHY28621.1	2,3,5	*Aphanomyces invadans*	Stramenopile/oomycete
Otaur1	XP_022840576.1	2,3,5	*Ostreococcus tauri*	alga
Csube1	XP_005643030.1+wgs	2,3,5	*Coccomyxa subellipsoidea*	alga
Crein1	XP_042921376.1	2,3,5	*C. reinhardtii*	alga (green)
Badel2	OZJ03333.1	2,3,5	*Bifigurartus adelaidae*	fungi

Pblak1	XP_018286137+wgs	2,3,5	*Phycomyces blakesleeanus*	fungi
Ptric2	XP_002180403.1+wgs	3,4	*Phaeodactylum tricornutum*	stramenopile/diatom
Bdend1	XP_006679927.1	2,3,5	*B. dendrobatidis*	fungi
Spunc1	XP_016607024.1	2,3,5	*Spizellomyces punctatus*	fungi
Cowcz4	XP_011270466.1	2,3,5	*C. owczarzaki*	pre-metazoan
Celeg1	NP_001379906.1	2,3,5	*Caenorhabditis elegans*	nematode
Dreri2-J6	NP_739567.3	2,3,5	*Danio rerio*	fish
Mmusc2-J6	NP_203971.2	2,3,5	*Mus musculus*	mammal
Hsapi2-J6	NP_055982.1	2,3,5	*Homo sapiens*	mammal
Dmela2-J6	NP_651026.1	2,3,5	*Drosophila melanogaster*	insect
Tadha1	XP_002107811.1	2,3,5	*Trichoplax adhaerens*	early metazoan
Hvulg1	XP_012566096.1	2,3,5	*Hydra vulgaris*	cnidarian

Fig. S8. Evolution of JmjD6-related sequences. A BLASTp search for sequences was performed to identify proteins whose JmjC domains were most closely related to that of *Dictyostelium* JcdI. *A,* the JmjC domains of the 71 most closely related were aligned (Fig. S7A), and their evolutionary relationships were examined using a maximum likelihood method implemented in IQ-tree. The tree with the highest log likelihood value is shown and is unrooted. The percentage of trees in which the associated taxa clustered together is shown at each branch. Branch lengths are measured by the number of substitutions per site. Red and green labels indicate proteins with JcdI-like or JcdH-like JmjC domains, violet labels are assigned to proteins most closely related to human JMJD6, and orange-labeled proteins are JMJD4-like. An alignment of the full-length sequences of select examples were searched for similarities, which are represented by boxes in the domain diagrams and referred to as homology domains. Only the JmjC and F-box domains have known functions. See Fig. S7B for origin of sequences, and Fig. 7D for a synopsis of this tree.

Fig. S9. JcdI nucleotide and amino acid sequences. The native (black) genomic sequence (DDB_G0270006 at dictyBase.org; Q55CL5 at UniProtKB) and corresponding synthetic cDNA sequence (blue) are shown. Translated amino acid sequence is above in black. Synthetic oligonucleotides are in violet or dark red. Restriction sites are underlined or in italics as indicated. Other features are as noted. Oligonucleotide sequences are listed in Table S1.

```
tgggtaacctggttgtggtggataaccttgtggtgggtaaccttgttgtggtggataacc Dictyostelium DNA
ttgtggtggataaccttgttgtggtgggtaaccttgttgtggtgggtatctaagaattat
ttaaaaattaaaaaaaaaaaaaaaaaaaaaaaaaaaaaaaaaataatgttaataaattaa
aatgataaattaatataaataaaaattaatataaataaacttacccttgttgtggataa
ccatgtggatattggtttggataacctgacatcctttatttgagtttaatttaaaattaa
aagaaagaaaaaaaaaaaaaaaaaaaaaaaaaaaaaaaaaaaggagagaaagataggatt
aaaaaaaaaaaaaaaaaaaaaaaaaatgaaatttaaagaaaatcgattagtttttatatt
ttataaagtagtattaaagtatgaaatttaacataaaaaagttctcacttacttgaaaat
aaaattgataatgaaaaaaaaaaaaaaaaaaaaaaaaaaaactttattttgaaaaaaata
tcaaaaaattaattttttttttattattatttttttttttattaattttttttttttttt
tacatttaaaaaatttgtgcatacatcataataatcggttggaacctaattatcataaaa
            5'-tgtgcatacatcataataatcggttggaa                 JcdI-KO-5'UpS
attaaagatatttttttcaaaaaaaaaaaaattaataaaatttgacccattttctcaggt
ggaacatttcaattttaaagaatttttttatttttttttttttatttattttttttaaata
ataaaaataataaaaataataaaagaatattatttttttttttacttattaattcaaaaaaa
ctatataattttaaacttcatttttatataaaaatttaaataaattgttttttttttttt

                                          M  V  V  L  K  N  T    7
taaaaaaaaaaaaaaaaaaaaaaataataaataaaataaaATGGTAGTTCTTAAAAACAC
                                GGATCCCATATGGTGGTGCTGAAAAATAC synthetic DNA (BamHI NdeI)
                                5'-GCGCGCATGGTAGTTCTTAAAAACA  JcdI-5'S (BssHII)
                            5'-TATCCATGGTATGGTAGTTCTTAAAAACAC JcdI-Ex1-S (NcoI)

 L  N  N  N  N  N  N  N  N  N  N  N  N  N  N  K  K  K  Q  F   27
ATTAAATAATAATAATAATAATAATAATAATAATAATAATAATAATAAAAAAAAACAATT
CCTGAATAACAATAATAATAACAACAACAACAACAATAACAATAACAAAAAGAAACAGTT
AT                                                           JcdI-Ex1-S (NcoI)
 Y  K  R  P  D  K  K  D  K  K  Q  E  I  I  N  S  K  G  V  K   47
TTATAAAAGACCAGATAAAAAAGATAAAAAACAAGAAATTATAAATTCAAAAGGTGTCAA
CTACAAACGTCCGGACAAAAAAGATAAAAAACAGGAAATCATTAATTCTAAAGGCGTGAA

 V  V  I  G  E  T  D  L  R  T  K  S  L  G  A  L  S  I  L  E   67 pred. F-box
AGTTGTAATTGGAGAAACAGATCTTAGAACTAAATCATTGGGTGCATTATCAATTTTAGA
AGTGGTTATTGGTGAAACCGATCTGCGTACGAAATCCCTAGGCGCCCTGAGCATTCTGGA

 D  Q  I  L  L  N  V  V  F  N  E  F  T  C  S  E  L  L  K  Y   87
GGATCAAATTTTATTGAACGTTGTTTTCAATGAATTCACATGTAGTGAACTATTGAAATA
AGATCAAATCCTGCTGAATGTCGTGTTTAACGAATTCACCTGCAGCGAACTGCTGAAATA
                     A  R           A                        F-box mutations
                     GCTAGA         GCA

 Q  C  V  S  P  A  F  Y  I  L  L  G  D  D  R  L  W  K  D  A  107
TCAATGTGTTAGTCCAGCATTTTATATTTTATTAGGTGATGATAGACTTTGGAAAGATGC
TCAGTGTGTGAGCCCGGCGTTTTACATCCTCCTAGGCGATGACCGTCTGTGGAAAGATGC

 F  L  R  E  I  K  G  R  K  E  F  V  K  Y  I  E  N  W  K  I  127
ATTCCTAAGAGAGATTAAAGGTAGAAAAGAATTTGTAAAATATATTGAAAATTGGAAAAT
CTTTCTGCGTGAAATCAAAGGTCGTAAAGAATTCGTTAAATACATCGAAAACTGGAAAAT

 S  A  L  S  Y  L  Y  P  N  S  F  N  K  F  K  K  P  Y  I  P  147
TAGTGCTTTATCATATCTTTATCCAAATTCTTTTAATAAATTTAAAAAACCATATATTCC
CAGCGCACTGAGCTACCTGTACCCGAACTCATTCAACAAATTCAAAAAACCGTACATCCC
                                                  GTATATAAGG
 L  H  F  P  D
ATTACATTTTCCAGgtaagttataacattaaaatattgattgaatgaatgaatgaattaa 152 intron
GCTGCATTTTCCGG
```

```
TAATGTAAAAGGCCTAAG-5'                                        JcdI-Ex1-AS (BspE1)

                            F  Y  S  H  E  V  Y  T  R  W  L 163
taaataaatctaattaattttctttagATTTCTATTCACATGAAGTTTATACGAGATGGT
                           ATTTCTATTCGCACGAAGTTTACACCCGTTGGC
              5'-CATTTTCCGGATTTCTATTCACATGA                  JcdI-Ex2-S (BspE1)

 R  R  H  M  K  V  K  D  Y  G  V  D  F  G  H  V  K  H  I  E 183
TAAGAAGACATATGAAGGTTAAAGACTATGGTGTTGATTTCGGACATGTTAAACATATTG
TGCGTCGTCACATGAAAGTCAAAGACTATGGCGTGGATTTCGGTCATGTTAAACACATTG

 S  D  E  L  T  V  E  E  F  Q  R  E  Y  E  I  P  S  I  P  V 203 Cupin 8 start
AGAGTGATGAGTTGACAGTTGAGGAGTTCCAAAGGGAGTATGAGATACCATCGATACCGG
AATCAGATGAACTGACCGTGGAAGAATTTCAACGTGAATACGAAATCCCGTCGATTCCGG

 I  F  K  N  A  Q  R  G  T  P  M  M  E  K  N  E  W  S  E  E 223
TTATATTTAAGAATGCACAGAGAGGTACACCAATGATGGAGAAGAATGAATGGTCAGAGG
TTATCTTCAAAAATGCGCAGCGTGGCACGCCGATGATGGAGAAAAACGAATGGAGCGAAG

 R  L  I  E  R  C  G  D  V  V  F  K  I  S  H  Q  D  N  K  R 243
AGAGATTGATAGAGAGATGTGGTGATGTTGTATTCAAGATATCACATCAGGATAATAAGC
AACGTCTGATCGAACGTTGCGGTGATGTTGTCTTTAAAATTAGCCACCAGGACAATAAAC

 I  Q  M  T  F  R  D  Y  C  Q  Y  M  K  T  Q  T  D  E  E  P 263
GTATTCAAATGACGTTTAGGGACTATTGTCAATATATGAAGACTCAGACTGATGAGGAGC
GTATCCAAATGACCTTCCGTGATTATTGTCAGTACATGAAAACCCAAACGGATGAAGAAC

 L  Y  V  F  D  Q  A  F  G  E  K  V  P  S  L  L  D  D  Y  N 283 start of JmjC
CATTGTATGTATTCGATCAGGCATTTGGCGAGAAAGTGCCATCACTATTGGACGATTATA JmjC domain
CGCTGTATGTGTTTGACCAGGCATTCGGCGAAAAAGTTCCGAGTCTGCTGGATGACTATA
                           CGCTCTTTCACGGTAGTCCTAGG-5'        JcdI-5'-AS (BamHI)
 I  P  K  F  F  P  E  D  L  F  K  Y  N  G  E  E  H  R  P  H 303
ATATACCTAAATTCTTTCCTGAAGATTTATTTAAATATAATGGCGAAGAGCACAGACCCC
ATATTCCGAAATTTTTCCCGGAAGACCTGTTTAAATACAACGGTGAAGAACATCGTCCGC

 F  R  W  I  V  I  G  P  E  R  S  G  A  S  W  H  I  D  P  A 323 H319A inactivation
ATTTCCGATGGATTGTAATCGGTCCAGAGAGAAGCGGTGCTTCGTGGCACATCGACCCTG
ACTTCCGTTGGATCGTGATTGGCCCGGAACGTAGTGGTGCTTCCTGGCATATTGATCCGG

 G  T  S  A  W  N  S  L  I  S  G  R  K  R  W  L  M  Y  P  P 343
CCGGCACGAGTGCCTGGAATTCGCTAATATCTGGCAGAAAGCGTTGGTTAATGTATCCAC
CAGGCACGAGTGCTTGGAATTCACTGATCTCGGGTCGTAAACGTTGGCTGATGTATCCGC

 N  F  T  P  Y  T  V  E  S  D  E  V  V  D  K  I  Y  G  S  P 363
CCAATTTCACACCATACACAGTAGAGTCGGACGAAGTAGTTGATAAAATCTACGGTTCAC
CGAACTTTACCCCGTACACGGTCGAATCCGACGAAGTGGTTGATAAAATTTATGGTAGCC

 P  S  L  L  W  L  L  E  V  Y  P  Y  L  P  P  D  Y  R  P  I 383
CTCCATCCCTACTTTGGCTATTAGAGGTTTACCCTTACCTACCACCAGACTATCGTCCAA
CGCCGAGCCTGCTGTGGCTGCTGGAAGTTTATCCGTACCTGCCGCCGGATTACCGTCCGA

 E  C  I  Q  E  P  G  E  T  I  F  V  P  G  G  W  W  H  M  V 403
TTGAATGTATTCAAGAACCTGGTGAAACTATATTCGTACCAGGTGGTTGGTGGCATATGG
TTGAATGCATCCAGGAACCGGGTGAAACCATCTTTGTTCCGGGCGGTTGGTGGCACATGG

 L  N  M  E  Q  S  I  A  V  T  Q  N  F  C  N  S  Q  N  F  D 423 end of Cupin
TTTTAAATATGGAACAATCGATTGCAGTAACTCAAAACTTTTGTAATTCTCAAAATTTCG
TCCTGAATATGGAACAGAGCATTGCAGTTACGCAAAACTTTTGCAATTCACAGAACTTCG
                                5'-CTGCAGTGTAATTCTCAAAATTTCG  JcdI-3'-S-Stop (PstI)

 E  V  C  S  D  L  A  N  D  Q  K  K  D  Y  D  D  F  K  K  H 443
```

```
ATGAAGTTTGTTCCGATTTAGCAAATGATCAAAAAAAAGATTATGATGATTTTAAAAAAC
ATGAAGTCTGTTCGGACCTGGCTAACGATCAGAAGAAAGACTACGATGACTTCAAAAAAC
ATGAAGT

  L   L   T   S   R   P   D   F   Q   N   K   F   N   Q   F   E   F   K   N   D 463
ATCTCTTAACTTCACGTCCTGATTTTCAAAATAAATTCAATCAATTTGAATTTAAAAATG
ATCTGCTGACCAGCCGTCCGGATTTTCAGAACAAATTCAACCAATTCGAGTTCAAAAACG

  Q   F   T   H   S   F   D   D   T   D   Y   W   D   L   I   I   K   S   I   L 483
ATCAATTCACTCATTCATTTGATGATACAGATTATTGGGATTTAATTATTAAATCAATTT
ACCAGTTCACCCACAGCTTCGATGACACGGACTATTGGGATCTGATTATCAGATCTATCC BglII

  N   N   S   N   L   K   F   N   E   K   P   I   I   I   N   N   N   N   N   N 503
TAAATAATTCAAATTTAAAATTTAATGAAAAACCAATTATAATTAATAATAATAATAATA
TGAACAATTCGAACCTGAAATTCAACGAAAAACCGATCATCATCAATAATAACAACAACA


  D   N   D   E   L   I   N   T   T   N   I   N   K   N   E   E   K   E   E   E 523
ATGATAATGATGAACTTATAAATACAACAAATATTAATAAAAATGAAGAAAAGGAAGAAG
ATGACAATGATGAACTGATCAACACCACGAACATCAACAAAAACGAAGAAAAAGAAGAAG

  E   L   K   K   K   E   E   N   R   K   K   Y   D   E   D   E   D   D   D   S 543
AAGAATTAAAAAAAAAAGAAGAAAATCGTAAAAAATATGATGAGGATGAGGATGATGATT
AAGAACTGAAGAAAAAAGAAGAAAACCGTAAAAAATACGACGAAGATGAAGATGACGATT

  D   L   K   V   K   S   K   Q   I   K   L   P   P   K   E   S   T   L   Y   L 563
CAGATTTAAAAGTAAAATCAAAACAAATTAAATTACCACCAAAAGAATCAACATTATATT
CGAACCTGAAAGTGAAATCGAAACAAATCAAACTGCCGCCGAAAGAAAGCACCCTGTACC

  E   M   P   N   S   G   Q   S   P   V   F   I   V   D   E   K   Y   V   I   K 583
TAGAAATGCCAAATTCAGGTCAAAGTCCAGTTTTCATTGTTGATGAAAAATATGTTATTA
TGGAAATGCCGAACAGTGGCCAGTCCCCGGTCTTCATCGTGGATGAAAAATACGTTATCA

  L   Y   C   S   E   L   G   G   E   K   S   W   S   T   E   L   F   L   Y   S 603
AATTATATTGTTCAGAATTGGGTGGTGAAAAGTCATGGTCAACAGAGTTATTCTTATATT
AACTGTACTGCAGTGAACTGGGCGGTGAAAAAAGCTGGAGCACCGAACTGTTTCTGTACT

  K   I   K   E   N   S   K   L   N   S   T   F   P   K   L   L   S   Y   G   N 623
CAAAGATTAAAGAGAATTCAAAATTAAATTCAACATTTCCAAAGTTATTATCATATGGTA
CCAAAATCAAAGAAAACTCAAAACTGAACTCGACGTTCCCGAAACTGCTGAGCTACGGCA

  I   K   D   L   L   G   D   K   L   Q   C   E   W   K   W   P   Y   I   V   T 643
ATATTAAAGATTTATTAGGTGATAAATTACAATGTGAATGGAAATGGCCGTACATTGTAA
ATATCAAAGACCTGCTGGGTGACAAACTGCAGTGTGAATGGAAATGGCCGTATATTGTGA

  E   Y   L   K   D   T   L   N   L   Q   D   V   Q   P   V   P   D   S   L   P 663
CAGAATATTTAAAAGATACTTTAAATTTACAAGATGTTCAACCTGTACCAGATTCATTAC
CCGAATACCTGAAAGATACGCTGAACCTGCAGGACGTTCAACCGGTCCCGGATAGCCTGC
                                    5'-GCGCGCTACCAGATTCATTAC J-ET-5'S

  Y   P   Y   P   S   P   P   E   E   D   E   N   D   D   E   N   D   E   D   D 683
CTTATCCATATCCATCACCACCTGAAGAAGATGAAAATGATGATGAGAATGACGAAGATG
CGTATCCGTACCCGAGCCCGCCGGAAGAAGACGAAAATGACGATGAAAACGATGAAGACG
CTTA

  E   E   E   D   I   K   V   L   D   D   N   L   V   D   F   L   V   E   K   V 703
ATGAAGAAGAAGATATTAAAGTATTAGATGACAATTTAGTTGATTTCTTAGTTGAAAAAG
ATGAAGAAGAAGATATCAAAGTCCTGGACGATAATCTGGTGGATTTTCTGGTGGAAAAAG
                              GACAATTTAGTTGATTTCTTAGTTGCAGCTG-5’ JcdI-3’-AS (PvuII)

  S   L   I   H   S   I   E   I   D   D   N   N   N   N   N   N   N   I   E   N 723
```

```
TATCATTAATTCATTCAATTGAAATTGATGATAATAATAATAATAATAATAATATTGAAA
TTAGCCTGATCCATAGCATCGAAATCGATGACAATAACAATAATAACAATAATATCGAAA
TATCATTAATTCATTCAATTGAAATTGATGATA-5’                          JcdI-KO3’Scr-AS

  N   E   K   Q   S   I   L   I   D   D   N   I   K   S   L   L   N   E   Y   K 743
ATAATGAAAAACAATCAATTTTAATTGATGATAATATTAAATCATTATTAAATGAATATA
ACAACGAAAAACAGAGTATCCTGATCGATGATAACATCAAATCCCTGCTGAACGAATACA

  K   D   K   W   L   P   W   K   N   Q   L   L   S   L   D   S   K   Y   I   S 763
AGAAAGATAAATGGTTACCTTGGAAAAATCAATTATTATCACTTGATTCAAAATATATTT
AAAAGGATAAATGGCTGCCGTGGAAAAATCAACTGCTGTCACTGGACTCGAAATACATTA

  N   H   W   N   W   N   G   L   P   P   H   L   R   S   Q   L   A   S   Y   L 783
CAAATCATTGGAATTGGAATGGTTTACCTCCACATCTTCGTTCACAATTGGCTAGTTATT
GCAACCATTGGAACTGGAATGGTCTGCCGCCGCACCTGCGTAGTCAGCTGGCATCCTATC

  P   I   D   K   T   E   L   I   D   Y   S   M   D   P   C   F   I   H   A   D 803
TACCAATTGATAAAACTGAATTAATAGATTATTCAATGGATCCATGTTTTATTCATGCAG
TGCCGATTGATAAAACCGAACTGATCGACTACTCAATGGACCCGTGCTTTATTCACGCCG

  L   T   D   E   N   V   L   G   I   E   S   T   S   E   I   I   V   L   E   K 823
ATTTAACTGATGAAAATGTTTTAGGTATTGAATCAACCTCTGAAATAATAGTATTAGAGA
ACCTGACCGATGAAAATGTCCTGGGTATCGAAAGTACGTCCGAAATCATCGTGCTCGAGA

  I   K   K   L   N   K   T   S   K   S   A   K   K   G   I   K   N   S   L   A 843
AAATTAAAAAATTAAATAAAACTTCAAAATCTGCTAAAAAAGGTATTAAGAATTCTTTAG
AAATCAAAAAGCTGAACAAAACCTCAAAATCGGCGAAAAAGGGCATTAAAAATAGCCTGG

  S   R   L   K   Q   L   N   Q   K   G   K   N   N   D   D   Q   D   C   K   L 863
CGTCAAGATTAAAACAGTTAAATCAAAAAGGCAAAAACAATGACGATCAAGATTGTAAAC
CCAGCCGTCTGAAACAGCTGAACCAAAAAGGTAAAAATAACGACGATCAGGATTGTAAAC

  V   E   V   K   K   T   I   K   I   W   D   P   K   Y   L   I   D   W   G   D 883
TTGTTGAAGTAAAGAAAACTATTAAAATTTGGGATCCAAAATATTTAATTGATTGGGGTG
TGCTCGAGGTGAAGAAAACCATCAAAATTTGGGACCCGAAATATCTGATTGACTGGGGCG

  S   K   I   G   D   R   W   Y   E   L   V   S   L   Y   I   S   V   F   A   L 903
ACTCAAAAATTGGTGATCGTTGGTATGAATTGGTTTCACTTTATATCTCGGTCTTCGCAT
ATAGCAAAATCGGTGATCGTTGGTATGAACTGGTTTCACTGTACATTTCGGTCTTTGCCC

  D   K   V   R   L   K   S   F   L   S   K   Y   L   L   P   N   S   N   K   E 923
TGGATAAAGTTAGATTAAAATCTTTCTTATCAAAATATCTTTTACCAAATTCAAACAAAG
TGGATAAAGTGCGTCTGAAAAGTTTCCTGTCCAAATATCTGCTGCCGAACAGTAACAAAG

  N   E   K   S   W   L   D   Y   Y   N   E   N   P   Q   N   F   I   K   R   A 943
AAAATGAAAAGAGTTGGTTAGATTATTATAATGAAAATCCTCAAAATTTCATTAAAAGAG
AAAACGAAAAATCCTGGCTGGATTACTACAACGAAAATCCGCAAAACTTCATCAAACGTG

  M   Q   Y   T   L   I   H   H   C   D   A   F   T   T   A   A   R   H   N   P 963
CTATGCAATATACTTTAATTCATCATTGTGATGCTTTTACAACTGCTGCAAGACATAATC
CAATGCAGTACACCCTGATCCATCACTGTGATGCTTTCACCACGGCGGCCCGTCATAACC

  L   L   R   N   Y   Q   T   I   D   E   L   A   N   S   I   W   N   L   D   V 983
CATTACTTAGAAATTATCAAACAATTGATGAATTAGCAAATTCAATTTGGAATTTAGATG
CGCTGCTGCGTAACTACCAAACGATTGACGAACTGGCGAATAGCATCTGGAACCTGGACG
                                 TTAAGTTAAACCTTAAATCTAC
                                             TTTAGATG
  *
TATAAaaataataaagaaaataataataataataataataataataataataataat
TGTGAACTAGT                                                   SpeI
ATATTCTCGAG-5’                                                JcdI-Ex2-AS (SacI)
```

TAGGATCAGGCTCA*AGATCT*-5’ J-ET-5'AS (*PvuII*)

atataaaaaagaaattaaataaataaaaagaaaaataaacacctttttttattttttgtat
taaattattagttttattatttatttgtattttattcatttttgcagaatctgctgcat
5'-*CTGCAG*AT J-ET-3'S (*PstI*)
cgcattgcaatctactggcatatcaacatcctggttcaattacttggtcatcttgtgaat
CGCATTGCAATCTACTG
cttgagttggtacctaatgatttgaattacaatttttcaaaattttccatctaattgtct
tagcaaaaatgacaattctcaaaaatagtattatttatttaatataaaagtaatacaatt
attgagaatagcatatagctaggacaattagacggaaattttttgagcaattgcaacccaa
agtgtcgtaaagcctcttttgttttcttgtgatttttttttttttttttttttttttt
ttttttttttttttttttgtaaaaagttatcttaccatttattgctatttgaaaatttta
aatgatttcaaaacagataaacataaaattttgatattctttaatccatattttaaaatt

Fig. S10. *Dictyostelium* JcdH nucleotide and amino acid sequences. The sequences correspond to DDB_G0280485 at dictyBase.org and Q54VA7 at UniProtKB, and confirmed by in-house studies. Primers are described and listed in Table S1. Other features including adjacent genes are listed at the right.

```
 continued reverse oriented fsjA gene-TTATACTAAATGATACCCTAAA fsjA (reverse compl.)
TTTAAATCACCACACGCAATGAATGGTACAATTAAACTATCTGTTTTTAAAAATTCATTA
TCATTTTCATTATCTATATTACAATTATTATTATTATTACTATTATTATTATTATTATTT
AAAATTTTATTATGATTTTCTAAAGCTGGATCAATAATTTTTGGATTATAATTTAAAGGT
GGTTGATAATTTCTACATAATATAAAATTTTCAAGACTTGATTCTCTACTACTACTTGGT
TTAACAAATGAAACATGTTCAAAAAATAATTTCATTTGTGAATACATTAAACTCATATCA
TCACCTTTAAACATTTTTGCAACAAATGTTCCACCAATCTTTAATGTATGTGTTGTAATA
TTTAATGCTGATAAAATTAATTGTGATTGTCCATAAAAATCGATATCATGTAAACCAGTC
ACTAATAAAAAAATTATATACATGTTAATATTTTATTATTATTATTTCCTTTTTTGTGAT
TTTTTTTTTTTTTTTTTTTTTTGGAAATTATTTTTAATATCTTACCATCAGGAGCACCAT
                                          5'-CATCAGGAGCACCAT JcdH-5'UTR-S

CTGAAATGATTAAATCAGCTAATGAACCATCAAAATGTGAAATGATTTGTTTTGAAGTTT
         5'-GCGCGCGCTAATGAACCATCAAAATGTGAAAT                 JcdH-5'-KO-S (BssHII)

CATATTTTGTAATATCACCTTTAATTTGAACGACACCTTTTAATGGTGCCATCTCTTGAA
GATCTACAGCAACGATTTTAACATCTGGATCTTTACCATCACCATAAATCCTTCTACTTA
ATACTTGACTCCAACTACCAGGTGCTGCACATAAATCTACAACACGTTTAACACCTTCAA
AAATTTGATATTCCTCATCAATTTGAATTAATTTAAATGCTGATCTTGCTCTCCATCCTT
                                               5'-CTCCATCCTT JcdH KO BSR Scr 5F

CTTCTTTAGCTTTTCTATAATAAATATCTCTTTTATCTTTTGATGATTTACCCATTATTT
CTTCTTTAGCTTTTCTATAATAAATATCTC

TTTATTTATTTTTTGGGAAAAAACTAATTTATATGATGGTTTATAAAAAAAAAAAAAAAA
AAAAAAATTTTTAAAAATAAATGAAATTTAAGTCTTCGTTTTTTTTTATCAAAACGAAAC
TAAAATGAAAAAAAATAAAAAATAAAAAAATAAAAATAAAAAAAAATATTTCCAAAATCT
TTTTTTTTTTTTTTTCATCCATAATTAATACAACAAAAAA

M  S  K  Y  N  E  E  N  L  N  E  D  V  E  L  Y  I  P  H  P     20
ATGTCAAAATATAATGAAGAAAACTTAAATGAAGATGTTGAATTATATATTCCACATCCA   60
                                    CAACTTAATATATAAGGTGTAGGT
F  G  V  K  P  K  G  N  F  Y  F  D  S  F  N  K  D  F           38
TTTGGTGTAAAACCAAAAGGAAATTTTTATTTTGATTCATTTAATAAAGATTTTgtaaat  120 intron
AAACCACGGATCC-5'                                                   JcdH-5'-KO-AS (BamHI)

atataaatttttttttttttattaatttttttattaatttattaattaatttataaaataa  180

         V  D  S  R  K  L  G  L  G  L  F  S  V  F  E  D  N     55 pred. F-box
ttattttagGTTGATAGTAGAAAATTAGGATTAGGATTATTTTCAGTATTTGAAGATAAT  240

F  I  I  D  S  I  L  D  L  L  S  A  E  E  L  T  K  T  M  N     75
TTTATAATTGATTCAATTTTAGATTTATTAAGTGCAGAAGAATTAACTAAAACAATGAAT  300
5'-ATAATTGATTCAATTTTAGATTTATTAAGTGCAGAAGAATTAAC                 JcdH-del-S1
                                                  5'-CAATGAAT  JcdH-del-S2

L  V  S  K  T  F  Y  I  Y  V  Q  E  E  E  Q  W  K  M  R  S     95
TTAGTTAGTAAAACATTTTATATTTATGTTCAAGAGGAGGAACAGTGGAAAATGAGATCA  360
TTAGTTAGTAAAACATTTTATATTTATGTTCAAGAGG

I  N  K  F  S  D  G  E  F  K  Y  Q  H  S  W  Q  Y  T  Y  K    105
ATTAATAAATTTTCAGATGGTGAATTTAAATATCAACATAGTTGGCAATATACATATAAA  420

S  N  C  D  K  S  F  S  K  E  P  I  P  I  Q  V  K  Y  F  Y    125
TCAAATTGTGATAAATCATTTTCAAAGGAACCAATACCAATCCAAGTGAAATATTTCTAT  480

S  D  Y  L  F  H  I  H  R  C  A  T  V  D  L  K  Q  F  E  H    145
```

```
TCCGATTATTTATTTCATATTCATAGATGTGCAACAGTTGATCTTAAACAATTTGAACAT  540

G  D  S  I  D  R  R  T  N  L  T  F  E  E  F  T  R  E  Y  L    165
GGTGATTCAATTGATAGACGTACCAATTTAACATTTGAAGAGTTTACAAGAGAATATTTA  600

I  P  N  K  P  V  I  I  S  D  A  C  K  D  W  A  A  S  K  N    185
ATACCAAATAAACCAGTGATCATATCTGATGCATGTAAAGATTGGGCAGCTTCAAAAAAT  660

W  T  R  E  T  L  A  E  K  C  G  D  V  K  L  Y  I  N  A  G    205
TGGACAAGGGAAACCTTGGCAGAGAAATGCGGTGATGTTAAACTTTACATTAATGCAGGT  720

V  F  M  N  V  K  D  F  F  Y  Y  S  E  H  C  K  E  E  M  P    225
GTATTTATGAATGTAAAAGATTTCTTTTACTATAGTGAGCATTGTAAAGAAGAAATGCCA  780

M  Y  L  F  D  H  Y  Y  G  E  K  V  P  S  L  L  E  D  Y  S    245 JmjC domain
ATGTATTTATTCGACCATTATTATGGTGAAAAAGTACCATCACTTTTGGAGGATTATTCA  840

A  D  A  Y  F  K  E  D  L  F  N  V  L  G  D  K  R  P  S  F    265
GCAGATGCTTATTTCAAAGAGGATTTATTCAATGTATTAGGTGATAAAAGACCATCATTT  900
 GTCTACGAATAAAGTTTCTCCTAAATAAGTTACATAA-5'                     JcdH-del-AS1

R  W  L  L  A  G  P  P  R  S  G  A  S  F  H  K  D  P  N  H    285
AGATGGTTATTAGCAGGTCCACCAAGATCAGGTGCTTCATTTCATAAAGATCCAAACCAT  960
                              GTCCACGAAGTAAAGTATTTCTAGGTTTG-5'  JcdH-del-AS2

T  S  A  W  N  A  V  I  T  G  R  K  K  W  I  M  Y  P  P  H    305
ACATCCGCTTGGAATGCTGTAATCACTGGTCGTAAGAAATGGATAATGTATCCACCACAT 1020
                                             5'-CTGCAGCCACCACAT JcdH-3'-KO'-S

V  V  P  P  G  V  Y  P  S  D  D  G  L  E  I  T  A  P  S  S    325
GTTGTACCACCAGGTGTTTACCCATCTGATGATGGTTTAGAAATTACTGCACCAAGTTCA 1080
GTTGTACCACCAGG

I  I  E  W  F  I  N  F  Y  E  K  P  D  N  D  H  D  E  K  D    345
ATAATTGAATGGTTTATTAACTTTTATGAAAAACCTGATAATGATCATGATGAAAAAGAC 1140

K  D  S  L  E  Q  Q  Q  Q  Q  K  E  N  D  K  K  N  T  R  F    365
AAAGATAGTTTAGAACAACAACAACAACAAAAAGAAAATGATAAAAAAAATACAAGATTT 1200

K  L  K  D  K  K  V  N  I  N  N  Q  Q  K  Q  N  N  N  N  E    385
AAATTAAAAGATAAAAAAGTTAATATTAATAATCAACAAAAACAAAATAATAACAATGAA 1260

N  D  N  E  I  E  T  Y  E  T  V  K  P  L  E  G  I  L  H  A    405 JmjC domain (cont'd)
AATGATAATGAAATTGAAACTTATGAAACAGTTAAACCATTAGAAGGTATTTTACATGCT 1320

G  E  M  I  F  V  P  S  G  W  Y  H  S  V  L  N  L  E  E  S    425
GGTGAAATGATATTTGTGCCATCTGGTTGGTATCATAGTGTTTTAAATTTAGAGGAATCA 1380

I  A  I  T  H  N  F  I  D  S  N  N  L  L  K  V  V  D  F  M    445
ATTGCAATCACTCATAATTTCATTGACTCAAATAATCTATTAAAAGTTGTTGATTTTATG 1440

A  T  K  K  K  K  D  L  Y  N  E  F  T  N  K  I  E  Q  A  Y    465
GCAACAAAGAAAAAGAAAGATTTATATAACGAATTTACAAATAAAATTGAACAAGCTTAT 1500

P  G  K  L  E  S  L  R  N  A  E  K  Q  K  Q  E  E  I  K  R    485
CCAGGTAAATTAGAGTCACTTAGAAATGCTGAAAAACAAAAACAAGAAGAAATTAAAAGA 1560

K  E  E  E  R  L  K  N  K  K  K  S  I  W  E  T  T  T  N  D    505
AAAGAAGAAGAAAGATTAAAAAATAAGAAAAAATCAATTTGGGAAACTACAACTAATGAT 1620
                                        5'-GGGAAACTACAACTAATGAT JcdH BsR 3 KO Scr
```

```
S   N   N   V   P   T   S   K   S   F   S   F   S   F   G   D   D   L   S   D    525
TCAAATAATGTACCAACTTCAAAATCATTTTCATTCTCTTTTGGTGATGATTTAAGTGAT 1680
TCAAATAATGTACCAAC         GTAAAAGTAAGAGAAAACCACTACTAAATTCCAGCTG-5’ JcdH-3’-KO-AS (PvuII)

N  *                                                         526
AATTAAAAAATTTTAAAAATTCAAAATAAATAAGTTCTTTTTTTAATAAAAATTTTATTT 1740

ATTTATTTATTTATTTATTTATTTATTTTCCTTTTTTTTCTTTTCTTTGTTTCTTTTTTG 1800 DDB_G0280487
ATTTCTTTTGGACAGCATGTGACCAAGATTTCTTTTGACTTGTTCTAAGTGAACGTGGAA 1860 (reverse compl.)
GTTTCCAACCTTTTTCAATTGCAGTTTCTGCGGCAGCAGTAAGTACGACTGGAATGATTT 1920
                              CGCCGTCGTCATTCATGCTGA-5’       JcdH-3'ext-AS

TACCTTTTTCTTTTCTCATTTTAATTCTTCTACCTTTTTCATCTTGATAAATTTTATTCT
CTGCATCAATTAATGTTAAACCATCTGCTGTAATTGTATCTTTTTTATTATTACTATTAA
ATTTATTTGTACTTGGTGATTCTTTTTTAAAGTTTGGTAATTTCTTTTTAACTTTTGATT
TATCATTAACTTCTTCTTCATCATCTTCATCATTATCATCATCATCATCACTATTATTAT
CATCATCATCAGATTCATCTTCATCTTCACCATCACTATTAAATGATAATGGTTTAACAG
TTGTTGTAATGATTTTATCTTCACTTGAATATTCTTTAATTTGAGTTTCTTGAATTGTTG
ATGGTTTGATTAAAACTTTAACTTTGTTACCTTTTTCATCGATTGTTTCTTCTTCTTTAA
TTTCTTCTTCTTCTTCTTCTTCATCATCATTTTCGATACCAAATTCATTTTCATGTTTTT
TTCTTGAATCTATAATTGATTGTTGAAATGCTTCTCTTTGTAATTTTACAAATTTCTTTT
GTTCTGCTTTTTCTCTTTTCTCTTTTTCTTCAATTTTCTTTTGTGCATATTCTCTTCTTG
CAACTTTTCTTCTATGAAAACCTGTTAAATAATCTTTTCTATCATGTTCATTATATGAAA
TCTCTTTAACTCTATCAATTGATGCTCCTATTGCTTTATAATTTGGTGCTTTAACTACTT
TTTTTTTATTTTGTTTAAATGGTGGTTTACTTGACATTTTTTTAAT
```

Fig. S11. Tagging the *jcdI* gene locus. *A*, the previously described tagging plasmid for *Dictyostelium culE* (14) was modified by replacement of its gene-specific 5'- and 3'-homology sequences with corresponding sequences from *jcdI*, as illustrated and described in detail in Methods. Oligonucleotides are described in Table S1 and Fig. S8. The linearized tagging DNA was electroporated into amoebae strains (Ax3 and *gnt1*$^{-}$) and transformants, expected to uniquely edit the *jcdI* locus as depicted, were selected in the presence of blasticidin S.
B, after selection, clones were screened by PCR with JET-OUT-S and JET-BSR-AS primers.
C, screening by Western blot analysis using anti-FLAG (mAb M2) yielded clones with a band consistent with the expected M_r value of 116,000, and the expressed protein is referred to as JcdI-FLAG. Dashed lines indicate where irrelevant lanes of the same gel were digitally removed.

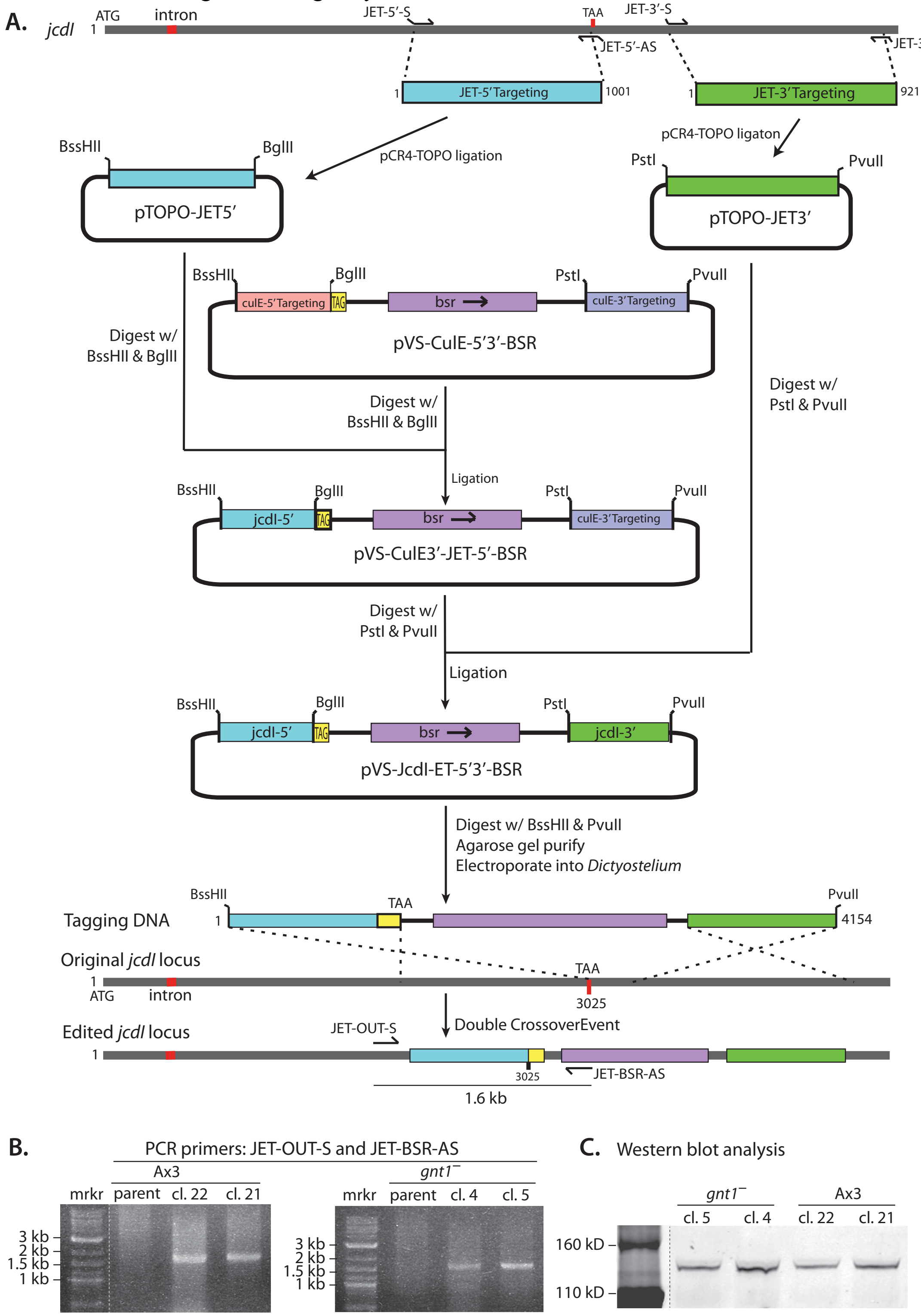

Fig. S12. Disruption of the *jcdI* and *jcdH* gene loci. Replacement DNAs were designed to integrate into their target loci via double cross-over homologous recombination, thereby replacing a region of the coding region with a floxed blasticidin S resistance cassette (*bsr*). PCR reactions with pairs of oligonucleotide primers (Table S1) residing either within the cassette or flanking the targeting DNA sequences were performed to assess the expected editing events. Expected products are shown at the left, and results are at the right.

A, strategy for *jcdI*. In addition, a replacement clone (cl. 7) was transiently transfected with a Cre-recombinase expressing vector to remove the floxed *bsr* cassette, enabling its reuse for replacing *jcdH*.

B, strategy for replacing *jcdH* in a *jcdI*⁻ strain (cl. BD3) from panel A.

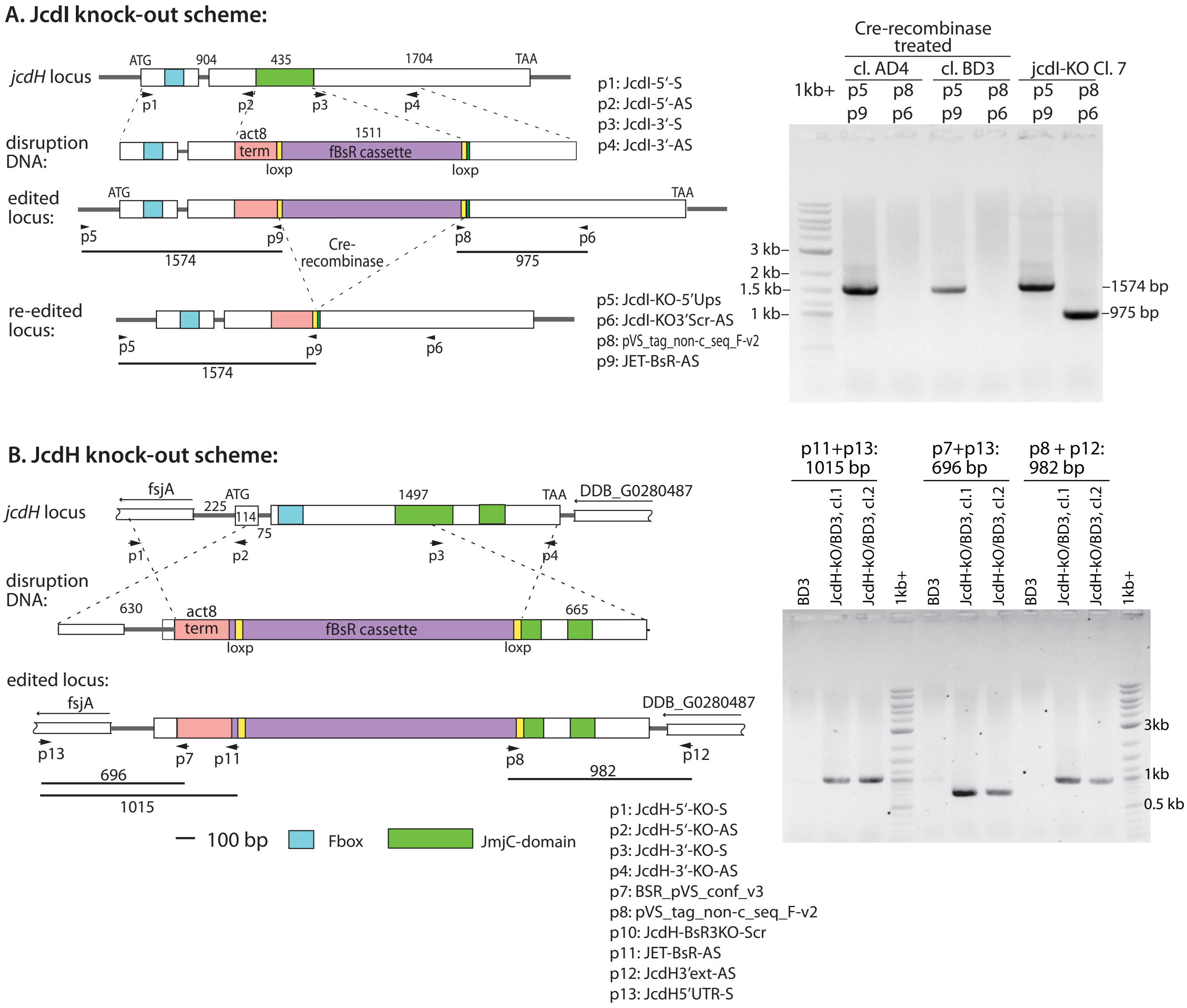

Supplement: Supporting information [file mmc1.pdf]
